# Supplementary material for: Conspecific and heterospecific grass litter effects on seedling emergence and growth in ragwort (Jacobaea vulgaris)
Source: PLoS One. 2021 Feb 2;16(2):e0246459. doi: 10.1371/journal.pone.0246459 (PMC7853490; doi:10.1371/journal.pone.0246459)
Supplement: S2 Table — (DOCX) [file pone.0246459.s002.docx]

**S2 Table. Results of a three-way ANOVA on the effects of litter type, litter amount and soil on ln biomass per plant.**

|  |  | **Ln Biomass per plant** | | | |
| --- | --- | --- | --- | --- | --- |
| **Source of variation** | df | MSQ | F | *P* | vc |
| **Litter type** | 2 | **0.024** | **72.685** | **≤ 0.0001** | **39.99** |
| **Litter amount** | 1 | **0.027** | **83.155** | **≤ 0.0001** | **22.87** |
| **Soil** | 1 | 0.000 | 0.851 | 0.359 | 0.23 |
| **Litter type x litter amount** | 2 | **0.008** | **24.878** | **≤ 0.0001** | **13.68** |
| **Litter type x soil** | 2 | 0.000 | 0.013 | 0.987 | 0.01 |
| **Litter amount x soil** | 1 | 0.000 | 0.056 | 0.814 | 0.02 |
| **Litter type x litter amount x soil** | 2 | 0.000 | 0.169 | 0.845 | 0.09 |
| **Error** | 84 | 0.000 |  |  | 23.11 |
