# Supplementary material for: Conspecific and heterospecific grass litter effects on seedling emergence and growth in ragwort (Jacobaea vulgaris)
Source: PLoS One. 2021 Feb 2;16(2):e0246459. doi: 10.1371/journal.pone.0246459 (PMC7853490; doi:10.1371/journal.pone.0246459)
Supplement: S1 Fig — In the box plots middle lines represent median, boxes represent the first and third quartiles, lower and upper bars represent the minimum and the maximum and points represent outliers (i.e. points above 1.5 SD). Grey: 200 g/m² litter, black: 400 g/m² litter. Lower case letters indicate significant interactions between treatments (TukeyHSD, P≤ 0.05). (DOCX) [file pone.0246459.s003.docx]

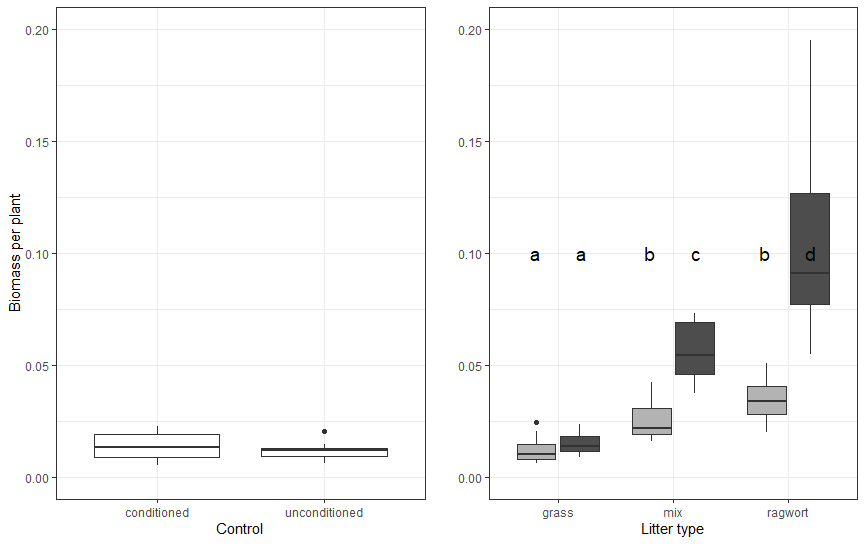


**S1 Fig. Biomass per plant - effects of the two-way interaction of litter type and litter amount.** In the box plots middle lines represent median, boxes represent the first and third quartiles, lower and upper bars represent the minimum and the maximum and points represent outliers (i.e. points above 1.5 SD). Grey: 200 g/m² litter, black: 400 g/m² litter. Lower case letters indicate significant interactions between treatments (TukeyHSD, P≤ 0.05).
