# Supplementary material for: Conspecific and heterospecific grass litter effects on seedling emergence and growth in ragwort (Jacobaea vulgaris)
Source: PLoS One. 2021 Feb 2;16(2):e0246459. doi: 10.1371/journal.pone.0246459 (PMC7853490; doi:10.1371/journal.pone.0246459)
Supplement: Attachment — Submitted filename: PONE-D-20-12615_reviewer.pdf [file pone.0246459.s004.pdf]

PLOS ONE

Conspecific and heterospecific litter effects on seedling emergence and establishment in ragwort (Jacobaea vulgaris Gaertn.)

--Manuscript Draft--

**Manuscript Number:**	PONE-D-20-12615
**Article Type:**	Research Article
**Full Title:**	Conspecific and heterospecific litter effects on seedling emergence and establishment in ragwort (Jacobaea vulgaris Gaertn.)
**Short Title:**	Litter effects on seedling emergence and establishment in ragwort (Jacobaea vulgaris Gaertn.)
**Corresponding Author:**	Henrike Möhler<br>Christian-Albrechts-Universitat zu Kiel<br>Kiel, GERMANY
**Keywords:**	Jacobaea vulgaris; mulching; litter; autotoxicity; ragwort; phytotoxicity
**Abstract:**	Jacobaea vulgaris Gaertn. or common ragwort is a widespread poisonous grassland weed that is subject to different regulation measures worldwide. Seedling emergence and establishment are the most crucial stages for most plants during their life cycle. Therefore, heterospecific or conspecific litter as well as soil-mediated effects may be of relevance for ragwort control. Our study examines the effects of conspecific and heterospecific litter as well as ragwort conditioned soil on seedling emergence and establishment.<br>We conducted pot experiments to estimate the influence of soil conditioning (with, without ragwort), litter type (grass, ragwort, grass-ragwort-mix) and amount (2 g/dm², 4 g/dm²) on J. vulgaris recruitment. As response parameters, we assessed seedling number, biomass, height and number of seedling leaves.<br>We found that, 2 g/dm² grass litter led to higher seedling numbers, while litter composed of J. vulgaris reduced seedling emergence. Litter amounts of 4 g/dm² had negative effects on the number of seedlings, regardless of the litter type. Significant effects of the soil conditioned by ragwort on seedling emergence were negligible. Results for biomass, plant height and leaf number showed opposing patterns to seedling numbers. Seedlings in pots treated with high litter amounts and seedlings in ragwort litter became heavier, grew higher and had more leaves.<br>The study confirms that the amount and composition of litter are strongly affecting seedling emergence and establishment of J. vulgaris. Moreover, while conspecific litter and high litter amounts negatively affected early seedling development in ragwort, those seedlings that survived accumulated more biomass and got taller than seedlings grown in heterospecific or less dense litter.
**Order of Authors:**	Henrike Möhler
	Tim Diekötter
	Geeltje Marie Bauer
	Tobias Wolfgang Donath
**Additional Information:**	
**Question**	**Response**
**Financial Disclosure**<br><br>Enter a financial disclosure statement that describes the sources of funding for the work included in this submission. Review the submission guidelines for detailed requirements. View published research articles from *PLOS ONE* for specific	This work was supported by the Stiftung Naturschutz Schleswig-Holstein (http://www.stiftungsland.de). We acknowledge financial support by Land Schleswig-Holstein within the funding programme Open Access Publikationsfonds (https://www.ub.uni-kiel.de/de/publizieren/open-access/uebernahme-von-open-access-publikationsgebuehren).<br>The funders had no role in study design, data collection and analysis, decision to publish, or preparation of the manuscript.

Powered by Editorial Manager® and ProduXion Manager® from Aries Systems Corporation

examples.<br><br>This statement is required for submission and **will appear in the published article** if the submission is accepted. Please make sure it is accurate.<br><br>**Unfunded studies**<br>Enter: *The author(s) received no specific funding for this work.*<br><br>**Funded studies**<br>Enter a statement with the following details:<br>• Initials of the authors who received each award<br>• Grant numbers awarded to each author<br>• The full name of each funder<br>• URL of each funder website<br>• Did the sponsors or funders play any role in the study design, data collection and analysis, decision to publish, or preparation of the manuscript?<br>• **NO** - Include this sentence at the end of your statement: *The funders had no role in study design, data collection and analysis, decision to publish, or preparation of the manuscript.*<br>• **YES** - Specify the role(s) played.<br><br>* typeset	
**Competing Interests**<br><br>Use the instructions below to enter a competing interest statement for this submission. On behalf of all authors, disclose any competing interests that could be perceived to bias this work—acknowledging all financial support and any other relevant financial or non-financial competing interests.<br><br>This statement **will appear in the published article** if the submission is accepted. Please make sure it is accurate. View published research articles from *PLOS ONE* for specific examples.	The authors have declared that no competing interests exist.

**NO authors have competing interests**<br><br>Enter: *The authors have declared that no competing interests exist.*<br><br>**Authors with competing interests**<br><br>Enter competing interest details beginning with this statement:<br><br>*I have read the journal's policy and the authors of this manuscript have the following competing interests: [insert competing interests here]*<br><br>* typeset	
**Ethics Statement**<br><br>Enter an ethics statement for this submission. This statement is required if the study involved:<br><br>• Human participants<br>• Human specimens or tissue<br>• Vertebrate animals or cephalopods<br>• Vertebrate embryos or tissues<br>• Field research<br><br>Write "N/A" if the submission does not require an ethics statement.<br><br>General guidance is provided below. Consult the submission guidelines for detailed instructions. **Make sure that all information entered here is included in the Methods section of the manuscript.**	N/A

**Format for specific study types**<br><br>**Human Subject Research (involving human participants and/or tissue)**<br>• Give the name of the institutional review board or ethics committee that approved the study<br>• Include the approval number and/or a statement indicating approval of this research<br>• Indicate the form of consent obtained (written/oral) or the reason that consent was not obtained (e.g. the data were analyzed anonymously)<br><br>**Animal Research (involving vertebrate animals, embryos or tissues)**<br>• Provide the name of the Institutional Animal Care and Use Committee (IACUC) or other relevant ethics board that reviewed the study protocol, and indicate whether they approved this research or granted a formal waiver of ethical approval<br>• Include an approval number if one was obtained<br>• If the study involved *non-human primates*, add *additional details* about animal welfare and steps taken to ameliorate suffering<br>• If anesthesia, euthanasia, or any kind of animal sacrifice is part of the study, include briefly which substances and/or methods were applied<br><br>**Field Research**<br><br>Include the following details if this study involves the collection of plant, animal, or other materials from a natural setting:<br>• Field permit number<br>• Name of the institution or relevant body that granted permission	
**Data Availability**<br><br>Authors are required to make all data underlying the findings described fully available, without restriction, and from the time of publication. PLOS allows rare exceptions to address legal and ethical concerns. See the PLOS Data Policy and FAQ for detailed information.	Yes - all data are fully available without restriction

A Data Availability Statement describing where the data can be found is required at submission. Your answers to this question constitute the Data Availability Statement and **will be published in the article**, if accepted.<br><br>**Important:** Stating 'data available on request from the author' is not sufficient. If your data are only available upon request, select 'No' for the first question and explain your exceptional situation in the text box.<br><br>Do the authors confirm that all data underlying the findings described in their manuscript are fully available without restriction?	
**Describe where the data may be found in full sentences. If you are copying our sample text, replace any instances of XXX with the appropriate details.**<br><br>• If the data are **held or will be held in a public repository**, include URLs, accession numbers or DOIs. If this information will only be available after acceptance, indicate this by ticking the box below. For example: *All XXX files are available from the XXX database (accession number(s) XXX, XXX.*).<br>• If the data are all contained **within the manuscript and/or Supporting Information files**, enter the following: *All relevant data are within the manuscript and its Supporting Information files.*<br>• If neither of these applies but you are able to provide **details of access elsewhere**, with or without limitations, please do so. For example:<br><br>*Data cannot be shared publicly because of [XXX]. Data are available from the XXX Institutional Data Access / Ethics Committee (contact via XXX) for researchers who meet the criteria for access to confidential data.*<br><br>*The data underlying the results presented in the study are available from (include the name of the third party*	All relevant data are within the manuscript and its Supporting Information files.

and contact information or URL).<br>• This text is appropriate if the data are owned by a third party and authors do not have permission to share the data.<br><br>* typeset	
Additional data availability information:	

Powered by Editorial Manager® and ProduXion Manager® from Aries Systems Corporation

Manuscript

Click here to download Manuscript Moehler_litter_effects_j_vulgaris_plos_twd.docx

Conspecific and heterospecific litter effects in ragwort (*Jacobaea vulgaris*) on seedling emergence and growth

Henrike Möhler[1] *, Tim Diekötter[1], Geeltje Bauer[1], Tobias W. Donath[1]

[1]*Department of Landscape Ecology, Institute for Natural Resource Conservation, Kiel University, Germany*

Henrike Möhler hmoehler@ecology.uni-kiel.de +49-431-880-1007

ORCiD 0000-0001-8070-3889

Tim Diekötter tdiekoetter@ecology.uni-kiel.de +49-431-880-1212

ORCiD 0000-0003-4838-793X

Geeltje M. Bauer geeltje.bauer@googlemail.com

Tobias W. Donath tdonath@ecology.uni-kiel.de +049-431-880 1198

ORCiD 0000-0003-1874-1096

*Corresponding author

hmoehler@ecology.uni-kiel.de (HM)

Conspecific and heterospecific litter effects on seedling emergence and establishment in ragwort (*Jacobaea vulgaris* Gaertn.)

Abstract

Jacobaea vulgaris Gaertn. or common ragwort is a widespread poisonous grassland weed that is subject to different regulation measures worldwide. Seedling emergence and establishment are the most crucial stages for most plants during their life cycle. Therefore, heterospecific or conspecific litter as well as soil-mediated effects may be of relevance for ragwort control. Our study examines the effects of conspecific and heterospecific litter as well as ragwort conditioned soil on seedling emergence and establishment.

We conducted pot experiments to estimate the influence of soil conditioning (with, without ragwort), litter type (grass, ragwort, grass-ragwort-mix) and amount (2 g/dm², 4 g/dm²) on *J. vulgaris* recruitment. As response parameters, we assessed seedling number, biomass, height and number of seedling leaves.

We found that, 2 g/dm² grass litter led to higher seedling numbers, while litter composed of *J. vulgaris* reduced seedling emergence. Litter amounts of 4 g/dm² had negative effects on the number of seedlings, regardless of the litter type. Significant effects of the soil conditioned by ragwort on seedling emergence were negligible. Results for biomass, plant height and leaf number showed opposing patterns to seedling numbers. Seedlings in pots treated with high litter amounts and seedlings in ragwort litter became heavier, grew higher and had more leaves.

The study confirms that the amount and composition of litter are strongly affecting seedling emergence and establishment of *J. vulgaris*. Moreover, while conspecific litter and high litter amounts negatively affected early seedling development in ragwort, those seedlings that survived accumulated more biomass and got taller than seedlings grown in heterospecific or less dense litter.

Keywords: Jacobaea vulgaris, mulching, litter, autotoxicity, ragwort, phytotoxicity

Introduction

Jacobaea vulgaris Gaertn. or common ragwort is a widespread poisonous grassland weed that is native in Europe, Asia and Siberia and invasive in North America, New Zealand and Australia. As the pyrrolizidine alkaloids of the plant pose a health risk to cattle (1), it is challenging farming when it occurs in high densities. Thus, control of common ragwort is prescribed in some countries and is as such included in their legislation (2). Consequently, several studies aimed at trying to find effective and efficient ways how to manage this grassland weed (2–5). Some of these studies come to the conclusion that specific cutting regimes are a promising approach (6). However, the toxicity of ragwort biomass precludes the use as hay or silage and a high number of long living seeds rules out composting the biomass. Consequently, the biomass is often disposed as special waste, which is costly (7). Yet, the occurrence of conspecific allelopathic effects (8, 9) suggests that leaving ragwort litter on the mown grassland could be an additional component in managing the abundance of ragwort. As a first step to evaluate the validity of this approach, we experimentally tested this management option.

Litter influences micro-environmental conditions, is important for nutrient cycling and can have prominent effects on seedling establishment and germination via mechanical and chemical effects (10, 11). Litter is affecting the physical environment by lowering light availability, changing the water and temperature regime and can act as physical barrier (11). Litter may also exert chemical effects by changing the availability of nutrients or releasing phytotoxins (9, 12, 13). Through these mechanisms litter potentially influences community organization via delaying and reducing or facilitating seedling emergence or seedling establishment (11, 14).

Albeit a meta-study on litter effects showed an overall negative effect of litter on seedlings (15), another meta-analysis demonstrated that litter can be also beneficial for seedling establishment especially under dry conditions (e.g. dry grasslands or dry periods) or at low to medium litter amounts (11). Positive effects were due to higher soil humidity and a lower amplitude of diurnal

temperature fluctuations beneath a low litter cover (12). In addition to the amount of litter, if seedling establishment is facilitated or hampered, also crucially depends on litter type (16). A thick litter layer may cause limited access to light for seedlings and poses a physical barrier for their growth. Grass litter, for example, might be less hampering than large-leafed forb litter due to a lower amount of phytotoxins and an easier to penetrate structure (13, 16). Under natural conditions, though, litter mostly is not monospecific, e.g. in grasslands it consists of a mixture of forb and grass litter. The effects of litter from plant communities are driven by the species-specific litter properties (17) and may either be purely additive as hypothesized in the mass-ratio-hypothesis (18) or non-additive as litter types may interact. In this context Gartner and Cardon (19) found that more than half of the litter mixtures they reviewed behaved in a non-additive way concerning factors like mass loss, nutrient transfer or decomposer composition.

It is intuitive to hypothesize that conspecific litter is facilitating the establishment of conspecific seeds. Yet, as intraspecific competition is assumed to be more intense, than interspecific competition (20) also inhibiting effects may be expected. While many studies have shown facilitating effects from adults on conspecific seedlings (21–23), particularly in weed species (24–26), there are also studies demonstrating the opposite (27, 28). Some did not find evidence of unidirectional conspecific facilitation (29). In line with the latter, studies on ragwort showed autotoxic potential of ragwort litter and soil from ragwort stands (30; 9). Also, a negative plant-soil-feedback has been demonstrated for ragwort, which is probably driven by differences in the soil community (31, 32). Under these circumstances, negative effects of ragwort litter and ragwort conditioned soil may either be additive, i.e. result in stronger inhibitory effects than they would do individually, or the combined effect is not additive, i.e. combined effects are lower or higher than the sum of both effect sizes. However, the influence of litter during seedling emergence might be weaker than during seedling establishment, since at the latter stage impacts of competition are of higher importance than direct litter effects (33). Thus, if litter

reduces seedling density through self-thinning surviving seedlings might be in advantage later on, as intraspecific competition is reduced.

The present study is to our knowledge the first to test the influence of the combined effects of litter type, amount and conditioned soil on seedling emergence and establishment in ragwort. To this end, we set up a common garden experiment, where we assessed the conspecific and heterospecific effects of litter on the number of emerged ragwort seedlings, their biomass, height, leaf number and specific leaf area. We posed the following questions:

I. Does litter type influence the emergence and establishment of ragwort seedlings?

Assuming conspecific negative effects, we expect lower numbers of ragwort seedlings to emerge from beneath conspecific litter compared to pure grass litter or a mixture of grass and ragwort litter. We expect congruent patterns to occur in seedling establishment after emergence.

II. Does litter amount influence seedling emergence and establishment of ragwort and does this depend on litter type?

We expect negative litter effects to increase with a higher litter cover and to be stronger with increasing litter amount. We also expect the size of litter effects to increase with the portion of ragwort litter in the litter cover. Thus, we expect an interaction of litter type and amount in that seedling's emergence and establishment is supposed to be lowest in high amounts of ragwort litter, higher in medium ragwort amounts and even higher in low amounts of grass litter.

III. Does ragwort-conditioned soil influence conspecific seedling emergence and establishment? If so, do these effects interact with the litter effects?

We hypothesized an inhibitory effect of ragwort-conditioned soil on its own emergence and establishment as negative plant-soil feedback for this species has been observed. We expect these effects to be additive for ragwort litter.

Material and methods

Study species

Jacobaea vulgaris syn. *Senecio jacobaea* or common ragwort is a herbaceous monocarpic plant from the Asteraceae family (34). It is a hemicryptophyte, forming a rosette in the first year and flowering in the second year (34). It prefers open patches and disturbed sites. Thus, it occurs on ruderal sites, along roadsides, pastures and grassland ecosystems in most temperate regions of the world. It is often regarded as a noxious weed due to its content of toxic pyrrolizidine alkaloids (2). Ragwort shows ecological characteristics of a pioneer species, in particular its numerous light seeds and fast growth enable ragwort to spread rapidly in open areas (1). Each plant produces several thousand seeds per year (1). Seeds are mainly dispersed by wind over a distance of usually less than 15 m (35). Seed survival (up to 20 years) and germination rates (about 80%) are high (36).

Experimental design

We studied the effects of *litter amount* (k = 2; 2 (low) vs. 4 (high) g/dm^2), *litter type* (k = 3; ragwort litter, grass litter, mix of ragwort and grass litter) and *soil conditioned* (k = 2; soil conditioned with ragwort vs. unconditioned soil) on seedling emergence and establishment. High litter densities refer to average regional productivity of grasslands in Northern Germany (37) and thus represent average litter quantities that ragwort seeds are exposed to at heavily ragwort-infested sites.

Seeds of *J. vulgaris* were hand-collected from at least 20 plant individuals on each of eight extensively managed pastures across Schleswig-Holstein from August to September 2015 and mixed for the experiment. These seeds were dry stored in darkness at room temperature until sowing on 21st April 2016. An initial viability test with a 1% tetrazolium chloride solution (38) on additional seed batches showed that most seeds (> 80%) were viable. Litter of *J. vulgaris* and conditioned and unconditioned soil was collected from rosettes on a ragwort-infested extensive cattle pasture near Preetz, Schleswig-Holstein in April 2016. We assumed soil affected by ragwort, i.e. conditioned, if at least four ragwort-plants were growing within 0.5 m^2. Unconditioned soil was taken from the center of an area (diameter 10 m) where no ragwort was growing. Litter was collected, cleaned and dried for two days at room temperature and for 24 hours at 40 °C. Litter levels of 2 g/dm^2 and 4 g/dm^2 were chosen, since Loydi et al. (11) found positive litter effects to occur at these litter amounts. As grass litter, we used dried plant material from a grassland mainly consisting of sweet grass (Poaceae) species.

We used a randomized experimental design. We prepared pots of 1 dm^3 volume (c. 10 × 10 × 10 cm) for each litter amount × litter type × soil conditioned (12 combinations) replicated eight times. To account for seedlings emerging from the soil taken from ragwort stands we additionally included 20 control pots, ten with ragwort conditioned soil and ten filled with unconditioned soil. Half of the pots was filled with a 6:1 mixture of commercial potting soil (Fruhstorfer Erde®, Type P, Industrie-Erdenwerke Archut GmbH, Germany) and ragwort-conditioned soil or unconditioned soil, respectively. We followed the protocol of van de Voorde et al. (39) concerning inoculation and mixing of soil, as it led to significant differences between conditioned and unconditioned soil in their experiment.

In late April 2016, we sowed 50 seeds in each experimental pot. Pots were placed randomly in a common garden in Kiel, Germany (N54° 20.81167 14, E10° 6.34518), surrounded by another

row of pots (to reduce desiccation) and protected by a light metal grid to avoid litter being blown away. Pots were kept constantly moist by regular watering.

Germination started mid of May 2016. Seedlings that emerged above the litter cover were counted weekly until the tenth week after the experiment started. On 30 June, we counted total number of leaves per plant and measured plant height of the highest plant in the pot. We determined specific leaf area for five pots per combination by cutting the youngest fully developed leaves of three plants. These were scanned and leaf area in mm^2 measured by the programme IMAGEJ (40). Afterwards leaves were dried at 60 °C and weighed to the closest milligram. The same was done with total aboveground biomass.

Data analysis

We tested differences in number of seedlings emerged and measures of seedling establishment represented by: total biomass per plant, plant height, leaf number and specific leaf area by calculating natural logarithmic response ratios (LnRR) to estimate the responses to the litter types and amounts and the soil type. LnRR were standardized with the mean of the control treatmen[illegible] our two control groups (unconditioned soil or no-litter treatments). Therefore, they were well suited to test for differences between the other factors. LnRR was calculated as proposed by Koorem et al. (41):

$$\text{LnRR} = \text{Ln (measured parameter value of the treated sample/mean value of the corresponding control)}$$

The effect of litter or soil was considered significant when the confidence interval did not overlap with zero (i.e. control and treatments are significantly different). To compare the LnRR among treatments, we used a factorial ANOVA to analyze the effect of litter type, litter amount and soil type, and their interactions. Graphical residual analysis revealed that the data met

assumptions of analysis of variance, i.e. normal distribution and variance homogeneity. We used the Tukey-HSD-Test as Post-hoc-Test. As a measure for the relative contribution of each factor and their interactions to the total variability of the response parameters of interest, we used the ratio of the sum of squares of the factor or interaction of interest to the total sum of squares (i.e. for all factors, their interactions and the error). In order to ensure readability, we refrain from prefixing the response variables with LnRR in the following text. All analyses were done using R version 3.6.1 (42).

Results

In general, our results showed significant main effects of litter type and litter amount on all response variables, i.e. seedling number, biomass per plant, plant height as well as on leaf number. In contrast, the factor soil, i.e. conditioned vs. unconditioned, influenced only plant height significantly. Our analyses revealed only one significant interaction between main factors, i.e. in case of biomass per plant we found a significant two-way *litter type* × *litter amount* interaction. Effects of the treatments on specific leaf area were not significant (results not shown).

Seedling emergence

While litter type and litter amount both affected seedling emergence significantly the amount of explained variance was more than twice as high for litter type than litter amount (37.88% vs. 17.01%; Table 1). Seedling number differed significantly between all three litter types but only grass and ragwort litter effects differed significantly from the control (95% CI does not include zero; Fig 1). In grass litter seedling emergence was one third higher (28 ± 1; mean ± SE) than in ragwort (18 ± 1). Interestingly, ragwort litter had a negative effect on seedling number across both litter amounts, while the opposite was true in case of pure grass litter. Across different

litter types an increased litter cover, i.e. 4 g/dm^2, led to a significantly reduced seedling number, both in comparison to the control and the low litter cover of 2 g/dm^2 (Fig 1).

Table 1. Results of a three-way ANOVA on the effects of litter type, litter amount and soil on LnRR of seedling number and measures of seedling establishment (biomass per plant, plant height and mean leaf number).

		LnRR Seedling number				LnRR Biomass per plant				LnRR Height				LnRR mean leaf number			
**Source of variation**	df	MSQ	F	*P*	vc	MSQ	F	*P*	vc	MSQ	F	*P*	vc	MSQ	F	*P*	vc
**Litter type**	2	**0.6003**	**37.578**	**≤ 0.0001**	**37.88**	**19.756**	**190.197**	**≤ 0.0001**	**62.42**	**9.844**	**113.713**	**≤ 0.0001**	**57.88**	**0.1173**	**8.206**	**≤ 0.0001**	**11.94**
**Litter amount**	1	**0.5393**	**33.755**	**≤ 0.0001**	**17.01**	**12.355**	**118.947**	**≤ 0.0001**	**19.52**	**2.459**	**28.400**	**≤ 0.0001**	**7.23**	**0.4330**	**30.296**	**≤ 0.0001**	**22.04**
**Soil**	1	0.0254	1.589	0.211	0.80	0.008	0.075	0.785	0.01	**3.947**	**45.599**	**≤ 0.0001**	**11.60**	0.0005	0.037	0.847	0.03
**Litter type x litter amount**	2	0.0211	1.321	0.272	1.33	**1.252**	**12.055**	**≤ 0.0001**	**3.96**	0.147	1.703	0.188	0.86	0.0305	2.137	0.124	3.10
**Litter type x soil**	2	0.0086	0.536	0.587	0.54	0.043	0.410	0.665	0.14	0.015	0.172	0.842	0.09	0.0057	0.398	0.673	0.58
**Litter amount x soil**	1	0.0004	0.027	0.871	0.01	0.004	0.042	0.839	0.01	0.055	0.637	0.427	0.16	0.0014	0.098	0.755	0.07
**Litter type x litter amount x soil**	2	0.0003	0.018	0.982	0.02	0.049	0.475	0.623	0.15	0.116	1.341	0.267	0.68	0.0109	0.761	0.471	1.11
**Error**	84	0.0160			42.40	0.104			13.80	0.087			21.49	0.0143			61.13

d.f. = degrees of freedom; MS = mean sum of squares; vc (%) = relative contribution of individual factors and their interactions to total variation.

Significant effects ($P < 0.05$) are given in bold.

Fig 1. LnRR of seedling number: effects of litter type, i.e. grass, mix, ragwort (left) and different litter amounts, i.e. 2 g/dm² litter and 4 g/dm² (right). Shown are the means ± 95% CI. Letters indicate significant differences between treatments (TukeyHSD, $P \leq 0.05$). Effects of treatments were considered significant (i.e. different from the controls) when 95% CI did not overlap with zero.

Seedling establishment

Effects of litter type and amount on biomass per plant were also significant but diametric to the results for seedling emergence (Fig 2). More than half of the variance within biomass per plant was explained by litter type, whereas litter amount only explained around one fifth of the variance (Table 1). The interaction between litter type and amount was also significant, but rather weak as it only accounted for around 4% of the explained variance. The interaction was quantitative in nature (Fig 2) as only the size but not the sign of the effect changed with litter type or litter amount, respectively. This means that the pattern of low litter amounts leading to higher seedling mass occurred in all litter types but was especially pronounced in ragwort litter compared to mixed litter and grass litter albeit seedling biomass was not significantly different from the control in the grass litter treatment.

While biomass per plant differed significantly between all three litter types, only mixed and ragwort litter treatment led to significantly more biomass per plant than in the control (95% CI does not include zero; Fig 2). Highest biomass amounts occurred in the treatment with high ragwort litter amounts.

Fig 2. LnRR of biomass per plant - effects of the two-way interaction of litter type and litter amount. Shown are the means ± 95% CI. Grey: 2 g/dm² litter, black: 4 g/dm² litter. Letters

indicate significant differences between treatments (TukeyHSD, $P \leq 0.05$). Effects of treatments were considered significant (i.e. different from the controls) when 95% CI did not overlap with zero.

Seedling height was significantly influenced by litter type and amount (Fig 3). The pattern is the same as for biomass as such that all litter types differed and seedlings in mixed and ragwort litter grew highest. More than half of the variance was explained by litter type (Table 1). Litter amount had a much smaller but also significant effect: higher litter amounts led to taller seedlings. Seedling height is the only response variable that was significantly influenced by soil type. Ragwort conditioned soil led to significantly lower height of seedlings. There was no interaction between the different factors. In all treatments, seedling height was different from the control. Only seedling height in grass litter was significantly lower compared to seedlings growing in the control pots. All other seedlings grew bigger.

Fig 3. LnRR of seedling height - effects of litter type, i.e. grass, mix, ragwort (left), different litter amounts, i.e. 2 g/dm² litter, 4 g/dm² litter (middle) and soil, i.e. ragwort conditioned, unconditioned (right). Shown are the means ± 95% CI. Letters indicate significant differences between treatments (TukeyHSD, $P \leq 0.05$). Effects of treatments were considered significant (i.e. different from the controls) when 95% CI did not overlap with zero.

Mean leaf number was significantly affected by litter type and amount (Fig 4). The explained variance for litter amount was twice as high as in litter type. Thus, leaf number is the only measured variable that seems to be stronger influenced from litter amount than litter type. Leaf number of plants in grass litter was significantly lower than in the control treatment, whereas leaf number in ragwort was higher, albeit not significantly, and leaf number of seedlings growing in mixed litter was comparable to the control. Higher litter amounts led to significantly higher number of leaves compared to the control treatment, while the opposite was true for the

low litter cover. Effects of the treatments on specific leaf area were not significant (results not shown).

Fig 4. Mean (± 95% CI) LnRR of mean leaf number under different litter types: grass, mix, ragwort (left) and different litter amounts: 2 g/dm^2 litter, 4 g/dm^2 litter (right). Shown are the means ± 95% CI. Letters indicate significant differences between treatments (TukeyHSD, P≤ 0.05). Effects of treatments were considered significant (i.e. different from the controls) when 95% CI did not overlap with zero.

Discussion

Our experimental results suggest that litter type as well as litter amount do influence seedling emergence and establishment. It is interesting to note that although ragwort litter reduced the number of conspecific emerging seeds, seedlings were actually performing better in ragwort litter, i.e. successful ragwort seedlings developed higher biomass, height and leaf numbers.

Litter type

While both, litter type and litter amount, influenced seedling emergence significantly, litter type accounted for a much higher portion of the variance than litter amount. In contrast, other studies reported that litter amount was more relevant than litter type (43, 44). This highlights the species specific nature of litter effects (13) and is a hint that ragwort litter is indeed autotoxic and negatively influences emergence of conspecific seedlings.

Seedling emergence was highest in grass litter, lowest in ragwort litter and intermediate in the litter mixture. The positive effect of grass litter might be due to physical litter effects like preserving a relatively constant temperature and soil moisture (45). In our experiment, the same amount of grass litter was covering a larger proportion of the soil surface than ragwort or mixed litter (high amount: grass 90 ± 3% (mean ± SE), mixed 85 ± 4% and ragwort 67 ± 8%, low amount: grass: 85 ± 3%, mixed 70 ± 9%, ragwort 37 ± 5%). In accordance to the study of

Eckstein and Donath (45), which revealed a rise in soil moisture when litter cover increased, we assume that seedlings experienced more favourable water availability in the grass litter. Laboratory experiments of van der Meijden et al. (46) demonstrated that seed germination of ragwort increased with higher soil humidity. We also observed that ragwort litter was decaying faster than grass litter (personal observation). This is in line with general findings that litter of forb species as ragwort, tends to decay faster than grass litter (47). Schuster et al. (48) found that the mass of ragwort litter remaining after 30 days was only half of the amount of grass litter. Since the protective properties of a litter cover against desiccation and temperature extremes increases with litter amount present, seedlings emerging from beneath faster decaying ragwort litter might have experienced less protection than those seedlings emerging from beneath grass litter (12, 45). Thus, the negative effect of ragwort litter on ragwort seedlings seems to be a result of fewer physical benefits seedlings experienced from conspecific litter compared to grass litter.

In general, the amelioration of the physical environment by litter, rather than its chemical composition, seems to be the main factor boosting seedling performance (17). However, the size of chemical autotoxic effects and therefore their relative importance in relation to physical and mechanical or other biotic effects are species-specific (49). Several studies show the occurrence of autotoxicity of ragwort (30; 9), which suggests that chemical effects are an additional mechanism for lower seedling emergence from beneath ragwort litter. Inline, van de Voorde (30) reported decreasing germination with increasing concentrations of ragwort extract. As ragwort produces many seeds with high germination potential and low dispersal ability (36, 1, 35) autotoxic effects may avoid intraspecific competition in ragwort and thus may lead to lower seedling numbers but increased seedling vitality.

Mixed litter resulted in fewer emerged seedlings than in grass litter, but more emerged seedlings compared to ragwort litter, which suggests that effects of grass and ragwort litter simply added

up to a neutral effect. This is supporting the mass-ratio hypothesis by Grime (18), i.e. mixed litter effects did not deviate positively nor negatively from the expected combined effects. In our study, the effect of mixed litter was not different from the control. Thus, from a management point of view, leaving litter on ragwort infested sites does not reduce or facilitate ragwort establishment.

Opposing to the findings for seedling emergence, seeds in ragwort litter built up more biomass, grew higher and tended to have more leaves than seedlings in grass litter. Biomass is the only variable where we found a significant interaction between litter type and litter amount. This interaction was quantitative and weak compared with the effects of litter type and amount alone (Table 1).

Seedlings emerging from beneath high amounts of ragwort litter built up the highest biomass. This is also diametrical to the findings of van de Voorde et al. (30), who reported lower biomass for seedlings in ragwort influenced environments. These conflicting results could be due to a different methodology. While van de Voorde et al. (30) harvested seedling biomass after 19 days, seedlings in our study were harvested about 50 days later. While van de Voorde's observation is supported by our personal impression, i.e. seedlings looked smaller and thinner early during development, they started to grow bigger after the ragwort litter had decomposed. Therefore, we might have observed a simple effect of density-dependence and intraspecific competition, which is further supported by the fact that lower seedling numbers from beneath grass litter resulted in seedlings of higher biomass. Already Loydi et al. (43) pointed out, that while litter plays a major role for seedling emergence for instance via phytotoxins, competition is more important for seedling development later during seedling establishment. However, indirect positive effects of reduced seedling emergence in boosting seedling biomass could be especially pronounced in situations where, like in the present experiment, intraspecific competition prevails and may overlain by different environmental constrains under field conditions (11, 50).

Another explanation for superior performance of emerged seedlings in ragwort litter might be an improved availability of nutrients (10), as ragwort litter decomposed quite fast. This is supported by our observation that even though the number of seedlings in the control pot and in the ragwort litter pot with 2 g/dm² was about the same and thus competition seedlings experienced was comparable, biomass of seedlings growing in ragwort litter pots was still higher.

Regarding leaf number, only seedlings in ragwort litter had significantly more leaves than plants in grass or mixed litter. This again might be linked to lower intraspecific competition as there were lower seedling numbers in the ragwort pots or by less physical hampering compared to grass litter (51).

Litter amount

We can affirm that ragwort emergence is influenced by litter amount, albeit less strongly than by litter type. Seedling emergence was lowest from beneath high litter amounts and higher from beneath low litter amounts, although not significantly different from the control. Positive effects could be due to physical effects such as constant moisture and temperature regime discussed above. Still, when litter cover is too high negative effects prevail over positive effects. Accordingly, compared to the control treatment, seedling emergence was lowest from beneath 4 g of litter and not reduced from beneath 2 g of litter cover. In case of ragwort, the reduced seedling emergence from beneath 4 g of litter is very likely linked to the relatively small seed mass of ragwort (0.22 mg; (52)). The smaller the seeds are, the less resources a seed can spent to successfully penetrate through a litter cover (53). In addition, small seeded species, like ragwort, tend to depend on higher light levels for germination than large seeded species (54, 55). Since photosynthetic active radiation (PAR) decreases with increasing litter cover (45) not only germination will be lower, but seedlings germinated will also lack light resources for successful penetration of the litter cover. This is also in accordance with van der Meijden and

van der Waals-Kooi (46), who found that emergence in ragwort significantly decreased when seeds were placed beneath a sand cover of more than 4 mm. Furthermore, Facelli et al. (56) showed that ruderal species, such as ragwort, are potentially negatively impacted by high amounts of litter. Therefore, ragwort seedlings might be reduced by a mulching regime on productive sites were litter amounts are medium to high.

The effects of litter amount on the establishment of seedlings was quite the opposite of the effects on seedling emergence. Thus, seedlings in dense litter got heavier (at least in mixed and ragwort litter), grew higher, and tended to have more leaves than seedlings in lower amounts of litter. This might be again a direct effect of lowered seedling densities in the first place and thus less competition. Another reason for smaller seedlings in lower amounts of litter could be due to the missing investment of seedlings in higher growth when they already reached light (51). Siebenkäs et al. (57) compared seedling growth for seedlings in the same developmental stage as seedlings in our experiment and found that plants tend to grow higher, when there is more shade. Thus, seedlings may grow higher in higher litter amounts to avoid its shade and in order to reach light. Another explanation for heavier and higher seedlings with more leaves could be higher nutrient content related to higher amounts of litter (33).

Conditioned vs. unconditioned soil

We expected potentially most inhibitory effects of ragwort litter on itself on sites that are infested with ragwort for a longer time. However, effects on soil on seedling emergence and establishment were very weak (Table 1). In fact, the only significant effect of conditioned soil occurred on seedling height, i.e. seedlings in unconditioned soil grew bigger than in conditioned soil. In general, this finding is in line with findings of van de Voorde (30), who also reported autotoxic effects of ragwort on its seedling development. However, on average the observed difference in plant height between ragwort seedlings growing in conditioned versus those

growing in unconditioned soil was only 2 mm (less than 4% of the average height of about 54.5 mm). Therefore, we expect that this difference is too small to be of ecological relevance. In other studies, looking at several seedling cohorts, those cohorts usually differed several centimeters in height (58–60).

Conclusion

We found both negative and positive effects of ragwort litter on conspecific seedling emergence and establishment. While negative effects can be most likely linked to autotoxic chemical and physical ragwort litter effects in the stage of seedling emergence, those seedlings that emerged successfully may have profited from a lower intraspecific competition and may had more resources to invest than seedlings that emerged from beneath grass or mixed litter or they profited from higher nutrient amounts in ragwort litter. Due to the ambivalent nature of our results, these findings cannot easily be translated into management recommendations for ragwort-infested sites. However, the effects of mixed litter on ragwort recruitment did not differ from the control treatment. Therefore, we expect that leaving litter containing ragwort biomass on the grassland will have no regulating influence on recruitment of ragwort seedlings.

References

1. Wardle DA. The ecology of ragwort (Senecio jacobaea L.): A Review. New Zealand Journal of Ecology 1987:67–76.

2. Leiss KA. Management practices for control of ragwort species. Phytochem Rev 2011; 10(1):153–63.

3. McEvoy PB, Cox C, Coombs E. Successful biological control of ragwort, Senecio jacobaea, by introduced insects in Oregon. Ecological Applications 1991; 1(4):430–42.

4. Suter M, Lüscher A. Measures for the control of Senecio aquaticus in managed grassland. Weed Research 2011; 51(6):601–11.

5. Jacobs J, Sing S. Ecology and Management of Tansy Ragwort (Senecio jacobaea L.). NRCS Montana Technical Notes-Invasive Species-MT-24 2009.

6. Siegrist-Maag S, Lüscher A, Suter M. Reaktion von Jakobs-Kreuzkraut auf Schnitt. Agrarforschung (Switzerland) 2008; 15(7):338–43.

7. Huckauf A, Rabe I, Aboling S, Böhling J, Böttner E, Ehlers B et al. Umgang mit dem Jakobs-Kreuzkraut: Meiden - dulden - bekämpfen. 4. überarbeitete Auflage. Flintbek: Landesamt für Landwirtschaft, Umwelt und ländliche Räume des Landes Schleswig-Holstein (LLUR); 2017. (Schriftenreihe LLUR SH. Natur; vol 25). Available from: URL: https://www.stiftungsland.de/fileadmin/pdf/JKK/senecio.pdf.

8. Wang R, Yang X, Song Y, Zhang M, Linhu, Su Y et al. Allelopathic potential of Tephrosia vogelii Hook. f.: Laboratory and field evaluation. Allelopathy Journal 2011; 28(1):53–62. Available from: URL: https://www.researchgate.net/profile/Ruilong_Wang/publication/287677172_Allelopathic_potential_of_Tephrosia_vogelii_Hook_f_Laboratory_and_field_evaluation/links/58735d0308ae8fce492390aa/Allelopathic-potential-of-Tephrosia-vogelii-Hook-f-Laboratory-and-field-evaluation.pdf.

9. Möhler H, Diekötter T, Herrmann JD, Donath TW. Allelopathic vs. autotoxic potential of a grassland weed—evidence from a seed germination experiment. Plant Ecology & Diversity 2018; 11(4):539–49.

10. Facelli JM, Pickett STA. Plant litter: Its dynamics and effects on plant community structure. The Botanical Review 1991; 57(1):1–32. Available from: URL: https://link.springer.com/content/pdf/10.1007%2FBF02858763.pdf.

11. Loydi A, Eckstein RL, Otte A, Donath TW, Gómez-Aparicio L. Effects of litter on seedling establishment in natural and semi-natural grasslands: A meta-analysis. J Ecol 2013; 101(2):454–64.

12. Loydi A, Lohse K, Otte A, Donath TW, Eckstein RL. Distribution and effects of tree leaf litter on vegetation composition and biomass in a forest–grassland ecotone. Journal of Plant Ecology 2014; 7(3):264–75.

13. Bonanomi G, Sicurezza MG, Caporaso S, Esposito A, Mazzoleni S. Phytotoxicity dynamics of decaying plant materials. New Phytol 2006; 169(3):571–8.

14. van der Putten, Wim H., Bardgett RD, Bever JD, Bezemer TM, Casper BB, Fukami T et al. Plant-soil feedbacks: The past, the present and future challenges. J Ecol 2013; 101(2):265–76.

15. Xiong S, Nilsson C. The effects of plant litter on vegetation: A meta-analysis. Journal of Ecology 1999; (87):984–94.

16. Donath TW, Eckstein RL. Grass and oak litter exert different effects on seedling emergence of herbaceous perennials from grasslands and woodlands. J Ecology 2008; 96(2):272–80.

17. Quested H, Eriksson O. Litter species composition influences the performance of seedlings of grassland herbs. Functional Ecology 2006; (20):522–32.

18. Grime JP. Benefits of plant diversity to ecosystems: immediate, filter and founder effects. J Ecol 1998; (86):902–10.

19. Gartner TB, Cardon ZG. Decomposition dynamics in mixed-species leaf litter. Oikos 2004; (104):230–46.

20. Antonovics J, Levin DA. The Ecological and Genetic Consequences of Density-Dependent Regulation in Plants. Ann. Rev. Ecol. Syst. 1980; (11):411–52.

21. Pleasants J M, Jurik T W. Dispersion of seedlings of the prairie compass plant, Silphium laciniatum (Asteraceae). American Journal of Botany 1992; (79):133–7.

22. Roll J, Mitchell RJ, Cabin RJ, Marshall DL. Reproductive Success Increases with Local Density of Conspecifics in a Desert Mustard (Lesquerella fendleri). Conservation Biology 1997; (11):738–46.

23. Montesinos D, García-Fayos P, Mateu I. Conflicting selective forces underlying seed dispersal in the endangered plant Silene diclinis. Int. J. Plant Sci. 2006; 167(1):103–10.

24. McEvoy PB. Seedling dispersion and the persistence of ragwort Senecio jacobaea (Compositae) in a grassland dominated by perennial species. Oikos 1984; Vol. 42(No. 2):138–43.

25. Wardle DA, Ahmed M, Nicholson KS. Allelopathic influence of nodding thistle (Carduus nutans L.) seeds on germination and radicle growth of pasture plants. New Zealand Journal of Agricultural Research 1991; 34(2):185–91.

26. Reinhart KO, Maestre FT, Callaway RM. Facilitation and inhibition of seedlings of an invasive tree (Acer platanoides) by different tree species in a mountain ecosystem. Biol Invasions 2006; (8):231–40.

27. Singh HP, Batish DR, Kohli RK. Autotoxicity: Concept, organisms, and ecological significance. Crit Rev Plant Sci 1999; (18):757–72.

28. Suzuki RO, Kudoh H, Kachi N. Spatial and temporal variations in mortality of the biennial plant, Lysimachia rubida: effects of intraspecific competition and environmental heterogeneity. J Ecology 2003; (91):114–25.

29. Hovstad KA, Ohlson M. Conspecific versus heterospecific litter effects on seedling establishment. Plant Ecol 2009; 204(204):33–42. Available from: URL: https://link.springer.com/content/pdf/10.1007%2Fs11258-008-9563-2.pdf.

30. van de Voorde, Tess F.J., Ruijten M, van der Putten, Wim H., Bezemer TM. Can the negative plant–soil feedback of Jacobaea vulgaris be explained by autotoxicity? Basic and Applied Ecology 2012; 13(6):533–41.

31. Bezemer TM, Harvey JA, Kowalchuk GA, Kopershoek H, van der Putten, Wim H. Interplay between Senecio jacobaea and Plant, Soil, and Aboveground Insect Community Composition. Ecology 2006:2002–13.

32. van de Voorde, Tess F. J., van der Putten, Wim H., Martijn Bezemer T. Intra- and interspecific plant-soil interactions, soil legacies and priority effects during old-field succession. Journal of Ecology 2011; 99(4):945–53.

33. Loydi, A. Donath, T. W. Eckstein, R. L. Otte, A. Non-native species litter reduces germination and growth of resident forbs and grasses: allelopathic, osmotic or mechanical effects? 2014 .

34. Harper JL, Wood WA. Biological Flora of the British Isles: Senecio jacobaea. Journal of Ecology 1957; 45(2):617–37.

35. McEvoy PB, Cox CS. Wind Dispersal Distances in Dimorphic Achenes of Ragwort, Senecio Jacobaea. Ecology 1987; 68(6):2006–15.

36. Crawley N. The Establishment of Seedlings from Primary and Regrowth Seeds of Ragwort (Senecio Jacobaea). Journal of Ecology 1985; 73(1):255–61.

37. Smit HJ, Metzger MJ, Ewert F. Spatial distribution of grassland productivity and land use in Europe. Agricultural Systems 2008; (98):208–19.

38. ISTA. Chapter 6: Biochemical test for viability: the topographical tetrazolium test. Int. rules seed test. 2015; 2015(1):6.

39. van de Voorde, Tess F. J., van der Putten, Wim H., Bezemer TM. The importance of plant-soil interactions, soil nutrients, and plant life history traits for the temporal dynamics of Jacobaea vulgaris in a chronosequence of old-fields. Oikos 2012; 121(8):1251–62.

40. Schindelin J, Rueden C T, Hiner M C, Eliceiri KW. The ImageJ ecosystem: An open platform for biomedical image analysis. Molecular Reproduction and Development 2015; (82):518–29.

41. Koorem K, Price JN, Moora M. Species-specific effects of woody litter on seedling emergence and growth of herbaceous plants. PLoS One 2011; 6(10):e26505. Available from: URL: http://journals.plos.org/plosone/article/file?id=10.1371/journal.pone.0026505&type=printable.

42. R: A language and environment for statistical computing. Vienna, Austria: R Foundation for; 2019. Available from: URL: https://www.R-project.org/.

43. Loydi A, Donath TW, Otte A, Eckstein RL. Negative and positive interactions among plants: Effects of competitors and litter on seedling emergence and growth of forest and grassland species. Plant Biol J 2015; 17(3):667–75.

44. Myster RW. Species-specific effects of grass litter mass and type on emergence of three tall grass prairie species. Ecoscience 2006; (13):95–9.

45. Eckstein RL, Donath TW. Interactions between litter and water availability affect seedling emergence in four familial pairs of floodplain species. Journal of Ecology 2005; 93(4):807–16.

46. van der Meijden E, van der Waals-Kooi, R. E. The Population Ecology of Senecio Jacobaea in a Sand Dune System: I. Reproductive Strategy and the Biennial Habit. Journal of Ecology 1979; 67(1):131–53.

47. Shaw RM, Harte J. Control of litter decomposition in a subalpine meadow–sagebrush steppe ecotone under climate change. Ecological Applications 2001; 11(4):1206–23.

48. Schuster MJ, Kreyling J, Berwaers S, Walter J, Boeck HJ de, Dengler J et al. Drought inhibits synergistic interactions of native and exotic litter mixtures during decomposition in temperate grasslands. Plant Soil 2017; (415):257–68.

49. Smith-Ramesh LM, Reynolds HL. The next frontier of plant–soil feedback research: unraveling context dependence across biotic and abiotic gradients. Journal of Vegetation Science 2017; (28):484–94.

50. Cavieres LA, Peñaloza A. Facilitation and interference at the intraspecific level: Recruitment of Kageneckia angustifolia D. Don (Rosaceae) in the montane sclerophyllous woodland of central Chile. Perspectives in Plant Ecology, Evolution and Systematics 2012; 14(1):13–9. Available from: URL:

https://ac.els-cdn.com/S1433831911000540/1-s2.0-S1433831911000540-main.pdf?_tid=504eb97b-fcf2-4e74-a32d-3bd51b9a3455&acdnat=1524728836_3de92c92b53c85c032201f4918134413.

51. Olson BE, Wallander RT. Effects of invasive forb litter on seed germination, seedling growth and survival. Basic and Applied Ecology 2002; 3(4):309–17. Available from: URL: https://ac.els-cdn.com/S1439179104700882/1-s2.0-S1439179104700882-main.pdf?_tid=1fcfe502-3db1-4ea1-8218-75099da6c252&acdnat=1524583667_44dede2fb9ae6a6adab43a7e509ac07d.

52. Fenner M. Relationships between seed weight, ash content and seedling growth in twenty-four species of Compositae. New Phytol 1983; (95):697–706.

53. Donath TW, Eckstein RL. Effects of bryophytes and grass litter on seedling emergence vary by vertical seed position and seed size. Plant Ecol 2010; (207):257–68.

54. Milberg P, Andersson L, Thompson K. Large-seeded spices are less dependent on light for germination than small-seeded ones. SSR 2000; 10(01):99. Available from: URL: https://www.cambridge.org/core/services/aop-cambridge-core/content/view/D6EC82DEDAAFE10F20C1D3A9A2328879/S0960258500000118a.pdf/largeseeded_spices_are_less_dependent_on_light_for_germination_than_smallseeded_ones.pdf.

55. Baskin CC, Baskin JM. Seeds: Ecology, Biogeography, and Evolution of Dormacy and Germination. Lexington: Academic Press; 2014.

56. Facelli JM, Montero CM, Leon R. Effect of different disturbance regimen on seminatural grasslands from the subhumid pampa. Flora - Morphology, Distribution, Functional Ecology of Plants 1988; (180):241–9.

57. Siebenkäs A, Schumacher J, Roscher C. Phenotypic plasticity to light and nutrient availability alters functional trait ranking across eight perennial grassland species. AoB Plants 2015; 7. Available from: URL: https://watermark.silverchair.com/plv029.pdf?token=AQECAHi208BE49Ooan9kkhW_Ercy7Dm3ZL_9Cf3qfKAc485ysgAAAbswggG3BgkqhkiG9w0BBwagggGoMIIBpAIBADCCAZ0GCSqGSIb3DQEHATAeBglghkgBZQMEAS4wEQQM2jFuXu_BEIq63rbbAgEQgIIBblqXOQs0WLQr398Ioj6Lq5NBvIL6i6Ov2dHtQb8B1no6uKgq0lmSDgTGVLdI-AbNlb8D2pjghvdZ_4wEcOGMPWB3xDsWcXnI2zlKInkXUvpipk7ODNpsUX_VY-4IJ5ln87uH2NPi53vrRCcPBxH70FmTAk8l-q0qrdE_5F9jHTgSdnjMjSikXysP1wvigdxiUm0QMjXUnueHzXeIE3GNyIjygSStnxLrn43gbYuyEdbQ1O65PrawU5L7-IfUgpu1SGXyfTGzW66A-ACjo2Uvg2GR0FmGvJ0ovOTjnTa7NymiQuF1QAlJ9YStjNdgCFklZbvpY9XK9wgXmrOwR2RI9qbqIHS_g8ZHLIKJ2TNggHoSonNyoJZLdYadJrUy7OeRNTHAwHUImj4dIiQ3rBmER-Wz8qCABafRrSbGO5mFJI9sf93cNIThTy7lA5AMU4bmKMIEKl4vEa7EY0mopASvev4VQSg-1yt0uOyhjLP1Lg.

58. Seiwa K. Effects of seed size and emergence time on tree seedling establishment: importance of developmental constraints. Oecologia 2000; (123):208–15.

59. Pausas JG, Ouadah N, Ferran A, Gimeno T, Vallejo R. Fire severity and seedling establishment in Pinus halepensis woodlands, eastern Iberian Peninsula. Plant Ecol 2003; (169):205–13.

60. Quintana J, Cruz A, Fernández-González F, Moreno JM. Time of germination and establishment success after fire of three obligate seeders in a Mediterranean shrubland of central Spain. Journal of Biogeography 2004; (31):241–9.

Supporting Information

S1 Table. Raw data for seedling emergence and seedling establishment according to different treatments.

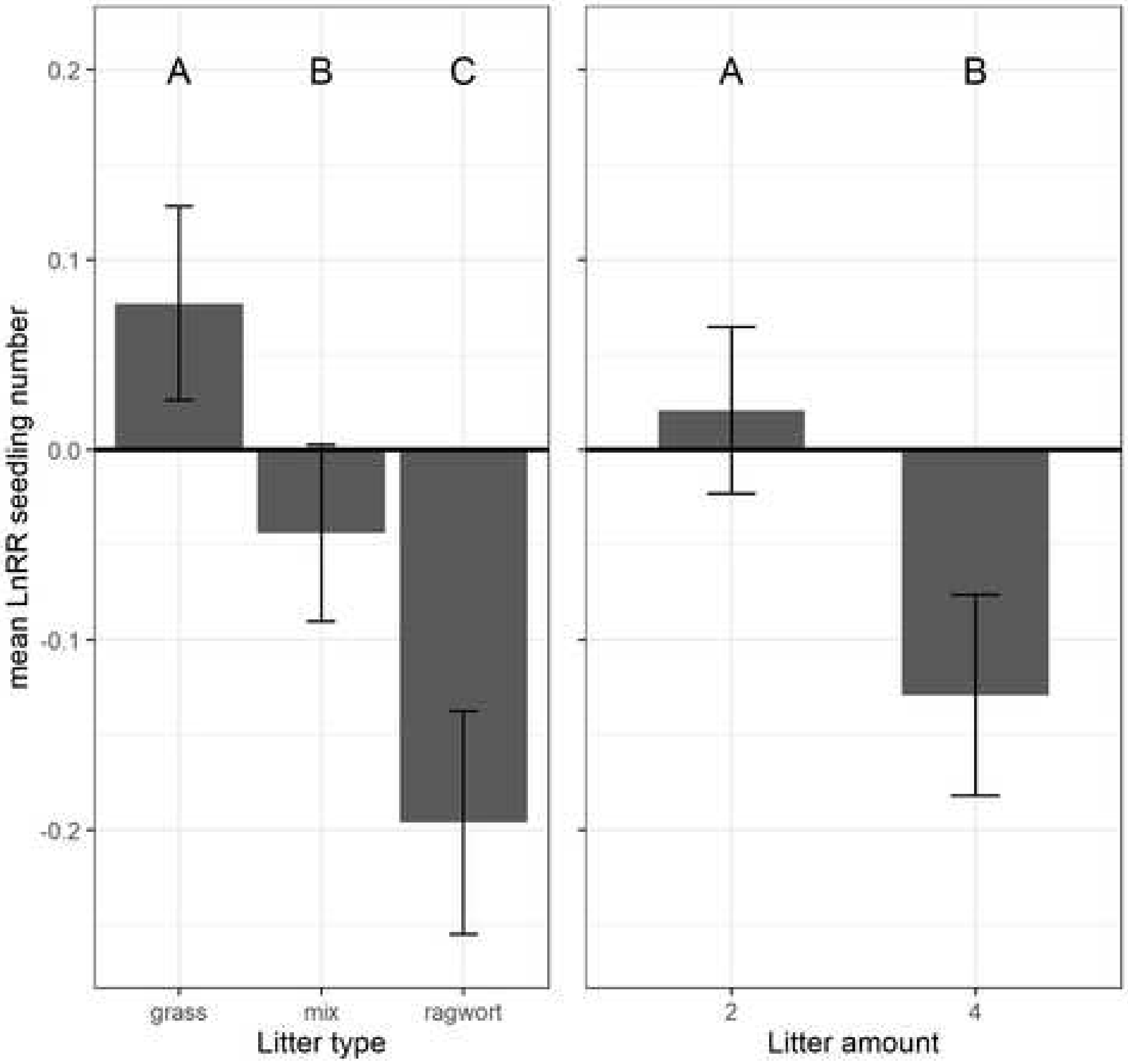
A
B
C
A
B
0.2
0.1
0.0
-0.1
-0.2
mean LnRR seedling number
grass
mix
ragwort
Litter type
2
4
Litter amount

Figure

Click here to download Figure fig2.tiff

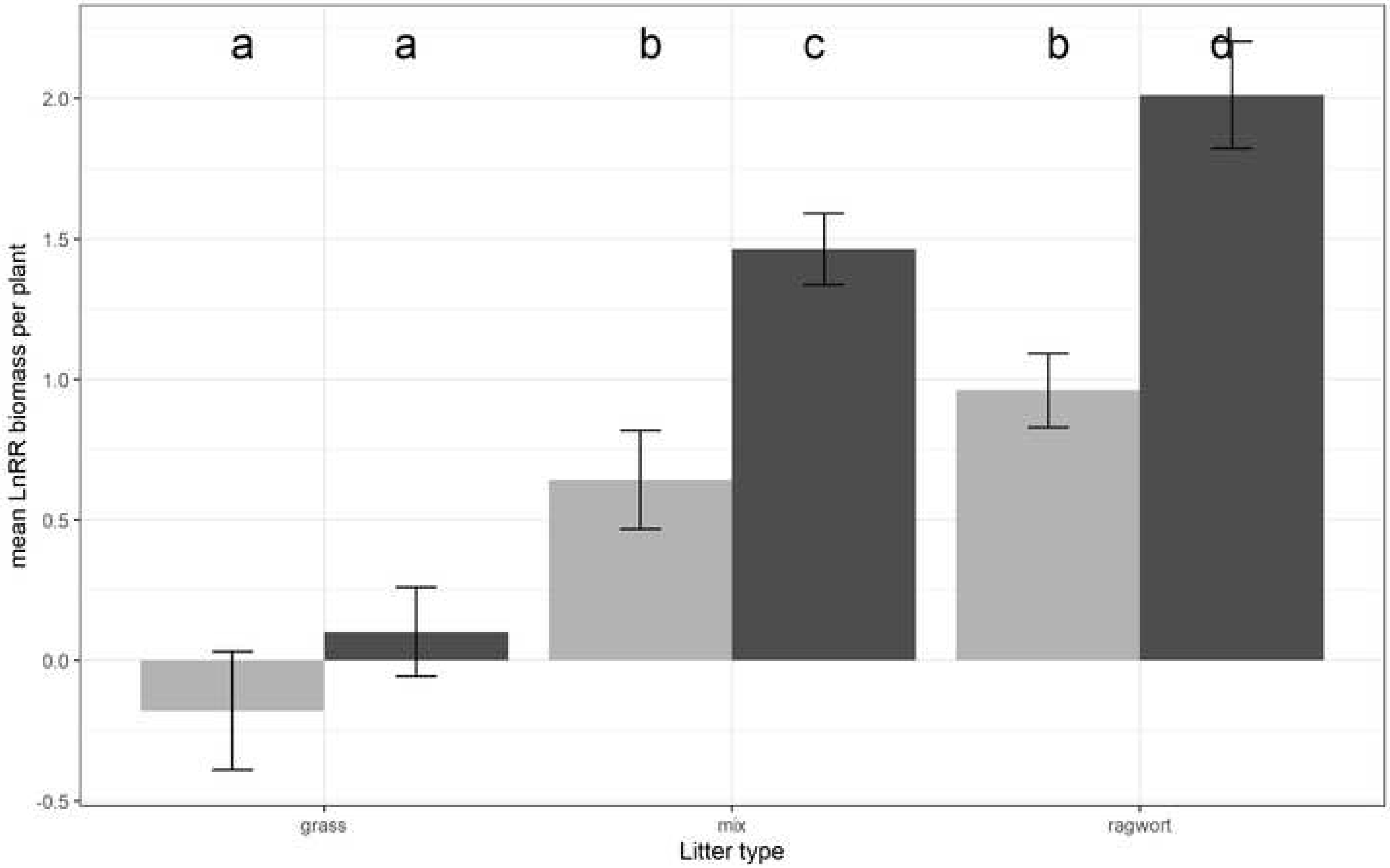

Click here to download Figure fig3.tiff

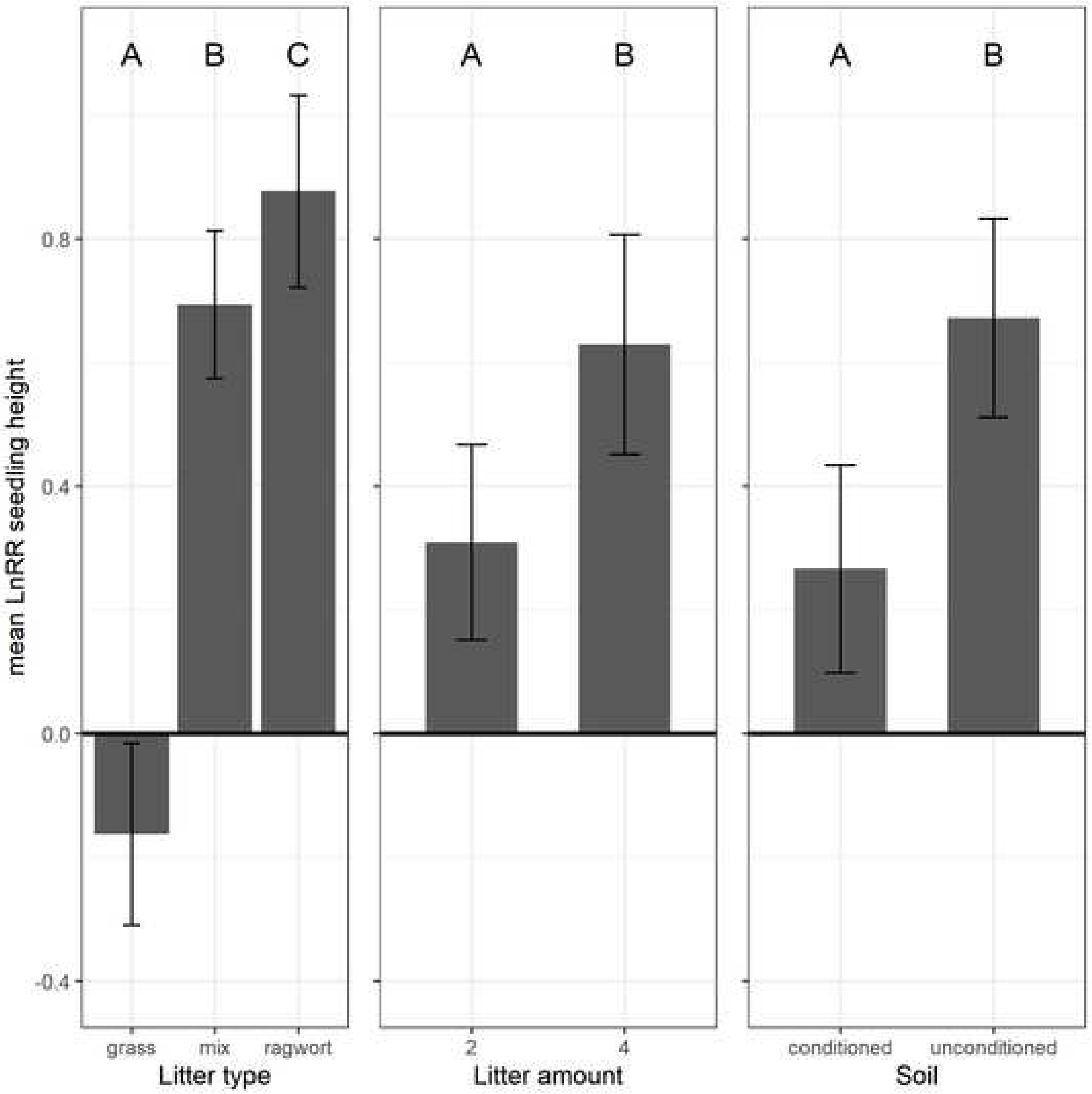

 Click here to download Figure fig4.tiff

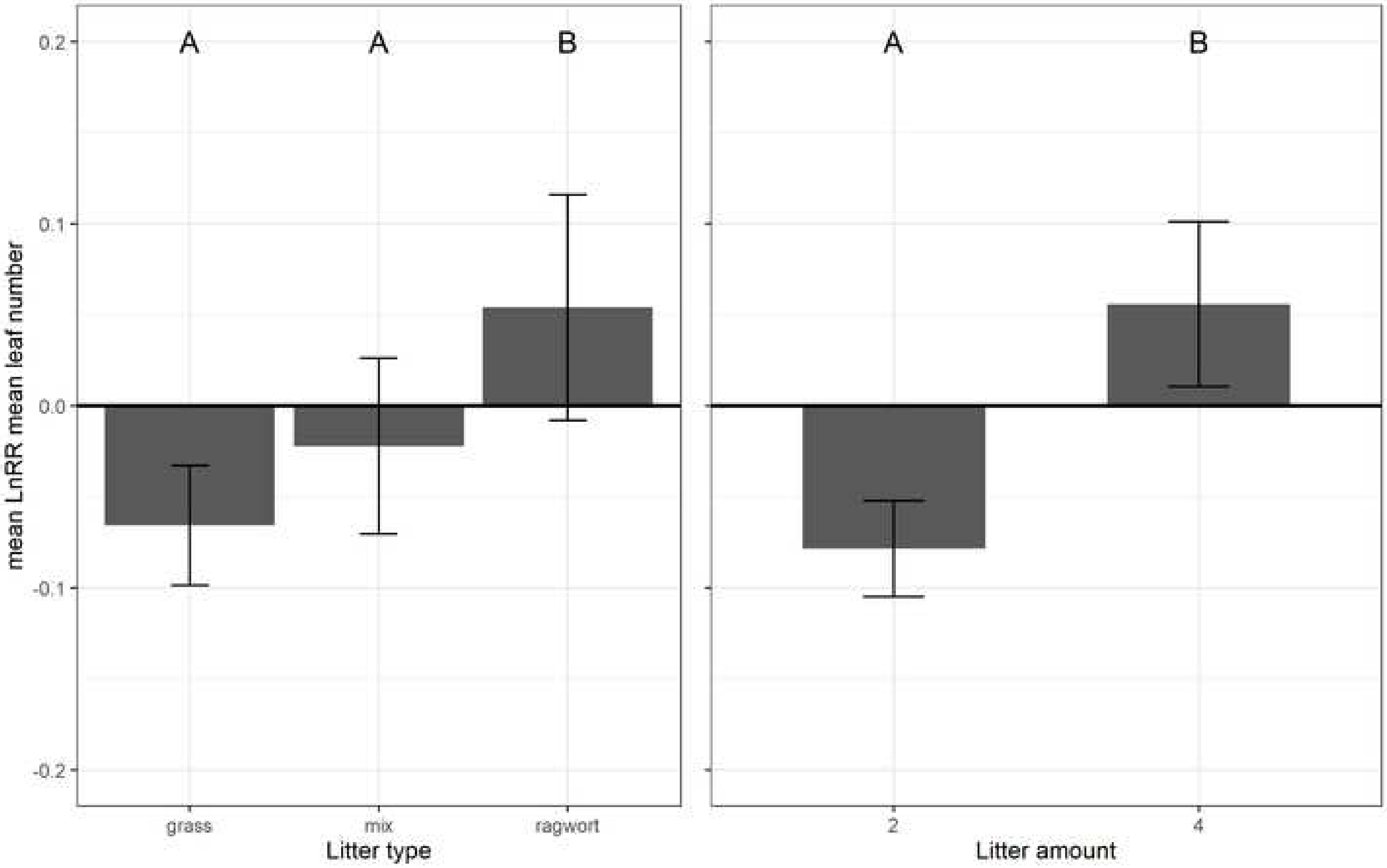

Click here to access/download
Supporting Information
tableS1.csv
