# Supplementary material for: Conspecific and heterospecific grass litter effects on seedling emergence and growth in ragwort (Jacobaea vulgaris)
Source: PLoS One. 2021 Feb 2;16(2):e0246459. doi: 10.1371/journal.pone.0246459 (PMC7853490; doi:10.1371/journal.pone.0246459)
Supplement: Attachment — Submitted filename: PONE-D-20-12615_R1_reviewer 1.pdf [file pone.0246459.s005.pdf]

PLOS ONE

Conspecific and heterospecific litter effects on seedling emergence and growth in ragwort (Jacobaea vulgaris)

--Manuscript Draft--

**Manuscript Number:**	PONE-D-20-12615R1
**Article Type:**	Research Article
**Full Title:**	Conspecific and heterospecific litter effects on seedling emergence and growth in ragwort (Jacobaea vulgaris)
**Short Title:**	Litter effects on seedling emergence and growth in ragwort (Jacobaea vulgaris)
**Corresponding Author:**	Henrike Möhler<br>Christian-Albrechts-Universitat zu Kiel<br>Kiel, GERMANY
**Keywords:**	Senecio jacobaea; mulching; litter; autotoxicity; ragwort; phytotoxicity
**Abstract:**	Jacobaea vulgaris Gaertn. or common ragwort is a widespread noxious grassland weed that is subject to different regulation measures worldwide. Seedling emergence and growth are the most crucial stages for most plants during their life cycle. Therefore, heterospecific or conspecific litter as well as soil-mediated effects may be of relevance for ragwort control. Our study examines the effects of conspecific and heterospecific litter as well as ragwort conditioned soil on seedling emergence and growth.<br>We conducted pot experiments to estimate the influence of soil conditioning (with, without ragwort), litter type (grass, ragwort, grass-ragwort-mix) and amount (200 g/m², 400 g/m²) on J. vulgaris recruitment. As response parameters, we assessed seedling number, biomass, height and number of seedling leaves.<br>We found that 200 g/m² grass litter led to higher seedling numbers, while litter composed of J. vulgaris reduced seedling emergence. Litter amounts of 400 g/m² had negative effects on the number of seedlings regardless of the litter type. Results for biomass, plant height and leaf number showed opposing patterns to seedling numbers. Seedlings in pots treated with high litter amounts and seedlings in ragwort litter became heavier, grew higher and had more leaves. Significant effects of the soil conditioned by ragwort on seedling emergence and growth were negligible.<br>The study confirms that the amount and composition of litter strongly affect seedling emergence and growth of J. vulgaris. Moreover, while conspecific litter and high litter amounts negatively affected early seedling development in ragwort, those seedlings that survived accumulated more biomass and got taller than seedlings grown in heterospecific or less dense litter. Therefore, ragwort litter has negative effects in ragwort germination, but positive effects in ragwort growth.
**Order of Authors:**	Henrike Möhler
	Tim Diekötter
	Geeltje Marie Bauer
	Tobias Wolfgang Donath
**Response to Reviewers:**	Dear Editor and Reviewers,<br>We thank you very much for your time, efforts and helpful comments and critiques. Based on both reviews we created an updated and improved manuscript.<br>The major changes we made were first, reanalyzing all the data with real observed data instead of derived Log response ratios. Second, adding a comparison between data for “mono” and mixed litter types. We also tried to give a more synthetic picture of the results and their context in findings of others in the discussion, which we hope is now shorter and clearer.<br>Unfortunately, we must disagree on some points, listed below. Further down, we would like to directly address these points, with your critique stated repeated (following R1/R2) and our answer (following A). Minor points were track changed directly in the manuscript.<br>In any case, we thank you for taking our manuscript into consideration anew and thank you again for your helpful critique and comments.<br>Yours sincerely

Powered by Editorial Manager® and ProduXion Manager® from Aries Systems Corporation

Henrike Möhler

____

Reviewer 1:

R1: The authors missed an opportunity to synthesize these observations by considering the per pot biomass of ragwort. The larger per-plant biomass of under ragwort litter is not inconsistent with a negative effect of litter on germination and early-plant growth. I expect that the total biomass – the sum of individual plants' biomass per pot – was lower in ragwort litter pots. That would offer evidence that ragwort self-limits itself and perhaps would offer a clearer management recommendation. “Self limitation” could be a broader concept that this study tests, if the authors choose. More importantly, the conclusion could change, as this finding would suggest a specific strategy for managers hoping to limit ragwort.

A: We added information on per pot biomass throughout the manuscript. However, as the results of per plant and per pot biomass do not differ in their general pattern (see figure below or figure added in supporting information), we cannot support reviewer 1's guess, that biomass was lower in ragwort litter pots and thus do not further discuss self-limitation. However, we did discuss self-thinning in the manuscript (line 324, 363, 415ff).

R1: The Discussion should be reorganized to be clearer and shorter. The division into headings based on the main treatments – litter type, litter quantity, and soil conditioning – prevented a synthetic consideration of the comparisons. I suggest that the Discussion more clearly compare the relative effects of each at the start of the Discussion so that the reader clearly gains a synthetic picture.

A: We reorganized the discussion accordingly and refrained from the division in headings based on the main treatments.

R1: Throughout your headings and text, you should phrase your measurement of ragwort “biomass” or “growth”, rather than “establishment.

A: We followed this advice.

R1: Line 125 – Cite the taxonomic authority as you did in the title. In fact, you can remove it from the title and just have it here.

A: We followed this advice.

R1: Line 138 – State that the mixture was 50:50.

A: We followed this advice.

R1: Lines 172-175 – Was biomass harvested after ~6 weeks, not 10? Why?

A: We corrected the wording. It was indeed 10 rather than 6 weeks.

R1: Lines 178-184 – I would like to see more comparison of these control treatments. Did they differ? Is there evidence that the conditioned soil had more seedlings? What is the justification for using the MEAN of these two treatments as your LnRR.

A: Control treatments did not differ unless for height. We added graphs of how the controls behaved for each parameter discussed.

R1: Results – Please emphasize more the direction of the effect primarily, and the “significance” secondarily. The reader only late in most paragraphs learns whether the effect of a treatment decreased ragwort, yet that is the key biological question.

A: We agree and followed this advice.

R1: Why are the figures set up so differently? I would like to see more consistency.

A: We followed this advice.

R1: Line 310-313 - Doesn't the (mostly) absence of an effect of soil conditioning refute that likelihood of allelochemical effect?

A: Allelochemical effects can act on very short time scales and not translate in soil effects. Thus, absence of soil conditioning and presence of autotoxicty are not contradictory.

R1: Abstract needs synthesis.

A: We followed this advice.

R1: Line 104-121 Hypotheses are clear. Nice job.

A: Thanks for this comment.

____

Reviewer 2:

R2: Perhaps my largest concern with the manuscript is that it is over-eager to draw conclusions outside the scope of the design. There is a fair amount of attention given to litter leachates as a possible inhibitory factor, but this wasn't tested and although likely, is not necessarily the sole explanation for the observed results.

A: We do not quite understand this claim. Maybe we might have not described the

design clear enough, thus we rephrased and added information on this in the manuscript now. We exposed seedlings of ragwort to ragwort litter and left the litter for decay under outdoor conditions. Thus, seeds were exposed to litter leachates that would also occur under natural conditions. It is certainly true that we did not only assess the chemical effects induced by leachate but also physical and mechanical litter effects. Therefore, we also discuss these in detail in the discussion section.
R2: More importantly, the manuscript concludes that leaving ragwort litter in the field has no net effect on ragwort establishment, but this is not adequately assessed by the presented analyses. To better understand this, the authors should evaluate whether grass-ragwort mixtures had an effect that deviated from the expected value based on those two litters in monoculture. Based on Fig 3, it appears that ragwort litter (which is different than green biomass – something that must be kept in mind) disproportionately stimulates germinant growth. If so, it would mean that incorporation of ragwort litter into mixtures with grass litter would result in more favorable conditions for germinants and that ragwort litter could facilitate a positive feedback loop favoring more ragwort. Managers should then seek to limit ragwort litter as much as possible (contrary to the current conclusion).
A: We added analysis on whether effects were purely additive or not. Indeed, concerning the two variables height and biomass per pot effects deviated positively, e.g. litter mix facilitated higher growth and led to higher biomass per pot. However, there was an only additive effect in biomass per plant and mean number of leaves. Furthermore, even though seedling vigor was positively influenced, seedling emergence was not. Thus, deriving a net effect favoring more ragwort cannot be concluded. Because of these ambivalent results we conclude that leaving or removing litter on the grassland will not substantially influence ragwort density. We clarified this in our conclusions.

R2: The analyses are structured around response ratios. Most of the results and discussion focus on differences in observed values and not on deviation from control values. So, there is an inherent disconnect between how data were analyzed and presented, and how they are discussed. This is confusing, and not particularly robust for most comparisons because it ignores variance of control units.
A: Log response ratios are a nice and valid approach to handle hanging control groups (in the current study the control pots (no litter) acted at the same time as the control for grass litter, grass-senecio-mix and pure senecio litter), but they come at the expense of some drawbacks; one (i.e. ignoring variance of control units) was highlighted by you and since we follow your argument we decided to rearrange the analyses. We changed the analyses and worked with the original to data make it easier to assess direct changes in factors like seedling number, biomass, height, and mean leaf number. In this approach, we did not include the controls in the complete ANOVA-model. We were interested in the differential effects of the three litter types; and not in the general differences of no litter cover vs. litter cover present (therefore, we did also not refer to a control litter treatment in our research question/expectations section).
Still we included a control in the experimental set up for informational reasons and tried to include this data in our original analyses via the log response approach – at the cost of the above-mentioned drawback. Based on the aim of our study and our research questions we could have also just skipped the controls completely from the experiment/manuscript but since they might be some merit reporting also in the updated manuscript we both included the controls in the graphs as well as doing an analyses control vs. litter treatments and analyzed for soil effects between the control treatments.
R2: When attempting to discern if litter mixtures behaved additively, the response ratio analyses make sense (although no such analysis is actually presented here – a missed opportunity since mixed litter studies are also uncommon).
A: We added analysis on whether effects were additive or not. To this end, we used contrasts to specifically test whether the effect of mixed litter on response variables was mid-way between that of grass and ragwort alone (additive effect of pure type litters in the mixture).
R2: In all other cases, using a simpler factorial design would be more accurate and more easily interpreted since it A) preserves variance of the controls, and B) directly reflects observed values. The structure of the analyses should be reconsidered.
A: We adjusted or analyses to observed values to make it easier to see direct changes in factors like seedling number, biomass, height, and leaf number. Controls are displayed next to the treatments we are interested in.

Powered by Editorial Manager® and ProduXion Manager® from Aries Systems Corporation

R2: The results are vague. It is not sufficient to only state that levels differed. Instead, the direction and magnitude of the change should also be presented in text (e.g. seedlings grew X% taller under ragwort litter than under grass litter). The results could also be clarified by tying statements of significance to specific tests in Table 1. As is, the table is presented in passing and it is unclear exactly which tests correspond to any given statement of significance.
A: We added information on direction and magnitude of change in the text and tied them to Table1.

R2: The introduction lacks detail and does not adequately establish the mechanisms tested. More detail is needed to address the specifics of how mechanisms are hypothesized to function. Stating only that litter facilitated a change in abiotic conditions is insufficient. Instead, it must be made clear how conditions changed (resource, direction, magnitude) and what that effect occurred.
A: We added information on the mechanisms.

R2: The introduction does not sufficiently support the hypotheses. Most of the introduction focuses on the question of how litter presence affects seedling establishment (vaguely described). However, the rationale behind testing litter amount and soil conditioning, as well as testing multiple species, is unclear. Each of these contrasts must be explicitly described in the introduction.

A: We cannot confirm this critique as we explicitly described effects of litter mix (line 90ff), litter amount (line 90ff) and conditioned soil (line: 102ff). However, we tried to better carve out those points.
R2 31. provide units in square meters
A: done

R2 48. “poisonous” requires some context – it is toxic to animals if consumed. This is important since “poisonous” is also used to describe things like Toxicodendron
A: We rephrased and avoided the word “poisonous”.

R2 51. you mean that it is challenging to raise cattle if ragwort is abundant?
A: We rephrased.

R2 56. citation required
A: Citation added.

R2: 58. If the concern is that cattle will consume the biomass (either in the form of hay or as living plants), wouldn’t leaving litter in place also be risky to cattle?
A: This is true. Thus, we would recommend farmers to remove cattle from the pasture when ragwort is cut and for few weeks later. Thereafter, ragwort litter is decomposed and not poisonous anymore.
R2:64. here and throughout, be more specific. “changing” is too vague and does not illustrate the mechanism.
A: We added information on mechanisms.

R2:66. The mechanisms have not been sufficiently described. More detail is needed in how and why litter affects microclimate and nutrient availability, either within the context of ragwort specifically or herbaceous weeds in general.
A: We added information on mechanisms.
R2: 68. paragraph needs a topic sentence and clearer structure. We jump from addressing seedling establishment directly, to abiotic impacts of litter, to litter mixing effects. Break these concepts up into separate paragraphs and provide clear transition between each.
A: We reworked the whole paragraph.
R2: 83. Is it intuitive? What about allee effectS?
A: Allee effects occur when populations reach a critical minimum size. As ragwort populations in our research area are very vivid and ragwort as a ruderal species is adapted to start new populations out of few seeds, we do not consider allee effects as an important for ragwort here.
R2:84. assumed by whom?

A: Reference is given.

R2:88. Is it just this one study or were there more?

A: There were more studies. Now cited.
R2: 89. Incomplete sentence – what did they show about litter and soil?

A: Sentence rephrased.
R2: 90. What is the feedback? Is it intra-specific or inter-specific? Provide detail.
A: Intraspecific was added

R2: 90. “differences” is too vague
A: Mechanism was added

R2: 91. is “probably driven by differences in the community” your speculation or that of the cited works?
A: speculation by cited work
R2: 92. Negative effects on which response?
A: Ragwort performance (biomass). Detail was added

R2: 95. the introduction should establish which factors are important to seedling establishment and seedling emergence as well as highlight differences between the two. Currently no support for this hypothesis is presented.
A: In line 74-84 we shortly discuss factors for seedling establishment and emergence. Furthermore, the support for this hypothesis is shown by the cited reference (Loydi et al. 2014).

R2: 103. In general, this section should not be necessary. It should be enough to state the hypotheses or questions without going back and explaining each. At the point that the hypotheses are presented, the reader should already be familiar enough with the mechanisms to understand the hypotheses without explanation. The current format of presenting a question and then detailing the predicted response is confusing and seems to be used instead of adequate background information beforehand. Furthermore, there are no citations presented within these explanations, so it is unclear what is hypothesis and what is already known.
A: We disagree; the comment of Reviewer 1 supports this by explicitly praising our clear hypothesis. The current format allows us to directly formulate our research hypothesis, which is derived from all the introduction written before the hypothesis are phrased. Thus, we have a clear section that sums up our assumptions based on all the information gathered in the introduction. Therefore, no further citations are included in this section. The current format allows us to build a frame for the study and is a clear guide to the reader, which questions he or she can expect to be answered.
R2:107. What do you mean by “congruent” – that there will be no difference between litter types?
A: We replaced congruent with similar.
R2: 114. Confusing. Break into multiple sentences.

A: We followed this advice.

R2: 124. This information would be more useful in the Introduction since the paper focuses on ragwort specifically. Also, additional information on the ecological effects of ragwort would be helpful.
A: We included all information necessary in the introduction already. This section allows us to explicitly portrait the studied species. “Information on ecological effects” of ragwort is rather vague. We could describe ragworts role as important habitat for insects or its positive soil feedback for other plants, but this would not be an important information for the current study. Thus, we refrained from it.
R2: 144. Schleswig-Holstein, Germany
A: We followed this advice.

149. Provide GPS coordinates
A: We followed this advice.

R2: 151. The soil is still conditioned, just not by ragwort. It would be more accurate to describe it as "grass-conditioned" rather than "unconditioned", assuming that the dominant species where soil was collected were grasses.

A: It is true that “unconditioned” soil as we phrase it, is truly conditioned by other plants than ragwort (including grasses and other forbs). However, as ragwort and effects of its litter are in the focus of our study and not “grasses and other forbs”, we find it more convenient to refer to ragwort conditioned soil as “conditioned” and not ragwort conditioned soil as “unconditioned”. We clarified this definition in our method-section.

R2: 153. That is a relatively low-intensity drying treatment. Did you verify that litter had reached constant mass by that point?
A: To dry litter at low temperatures allowed us to preserve all possible influential chemicals within the litter, which are part of the complex of litter effects. Still, we had the same concern you stated in earlier litter experiments; as a consequence we tried to assess how much more additional weight litter loses when dried at higher temperatures (110°C) some years ago; obviously the additional loss varied with the litter type but in most cases this loss was well below 5% and in no case above this value (not published personal observation).

R2: 154. How do these litter treatments correspond to natural conditions?
A: This has been explained in the manuscript (line 158).

R2: 155. This is a weakness of the experimental design that the authors must address. The study focuses on specific litter effects, and while ragwort is maintained as a specific treatment, the grass litter treatment is poorly controlled. The exact composition of the grass litter treatment is unclear. Ideally, this would have been a single grass species, but instead appears to be a presumably variable mixture of different species. This severely limits the authors’ ability to detect litter mixing effects since the baseline additive condition would vary with the composition of the grass litter. This is true for all litter experiments to some extent since litter quality is variable even within a single plant, but by including multiple grass species as a single treatment, this error is enhanced.
A: In fact, using a litter mixture as comparison is a strength rather than weakness as it mirrors natural conditions we wanted to mimic. For our research question we wanted a comparison of litter without ragwort, litter of pure ragwort and a mix of the two. We were not interested in pure grass effects as this does not lead to an answer of the evaluation if ragwort litter can act as establishment barrier for ragwort. As we found clear effects of grass litter vs. ragwort litter despite using the rather uncontrolled grass treatment, actually supports the clearance and strength of the patterns we found.
R2: 158. Where are data from these pots presented, or how were they considered in the analyses? If litter type and amount affects germination, wouldn’t you also need to expose control pots to the same conditions to accurately account for seedbank under the considered conditions?
A: Control pots for accounting for seedlings from the seed bank where used to control for the probability of extra-emerging seeds from the seed bank. As no seedlings emerged no corrections of seedling numbers were needed.
R2:165. When was litter applied?
A: At setup of the experiment. Information was added.
R2: 214. This is a 56% increase, not a 33% increase. This is also somewhat confusing because your analyses were using LnRR and not on the actual observed data. So, when we look at Fig 1, we do not see a 56% increase, we only see that ragwort litter suppressed emergence and grass stimulated emergence.
A: We corrected this.

R2: 215. “interestingly” is not needed
A: We deleted this.

R2: 216. Unclear. What do you mean by “opposite?” Where is this shown? Was there a species by amount interaction – Fig 1 shows no effect of 2 g/dm2 of litter.
A: We rephrased this part.

R2: 217. There is an over-reliance on “i.e.” throughout the manuscript. Be concise.
A: We corrected this.

R2:218. Also worth noting that 2 g/dm2 had no impact.
A: Changed according to new analysis.

Powered by Editorial Manager® and ProduXion Manager® from Aries Systems Corporation

R2: 219. MSQ is not necessary since you have provided F statistics
A: This information might seem superfluous on first sight. Still, it helps the reader to follow the statistical analyses, which is not the case when only the F-value is presented. Therefore, we would like to keep the MSQ in the table.

R2: 236. all interactions are quantitative – overly wordy. Erroneous bold.
A: Interactions can either be quantitative (seize of the effect differs) or qualitative (sign/direction of the effect changes). We want to stress that the direction (sign of the effect: low litter amounts leading to higher seedling mass) is the same we for all litter types. Thus, we want to keep this point.

R2: 238. Fig 2 does not support this since grass litter error bars overlap and do not differ from 0 (controls).
A: This is true and already discussed in the very same sentence of the original manuscript. But the whole paragraph has been reworked.
R2: 241. This appears to be redundant with line 239.
A: The whole paragraph has been reworked.

R2: 255. new paragraph
A: The whole paragraph has been reworked.

R2: 259. Quantify change. This is an issue throughout the results.
A: The whole results part has been reworked according to this critique.

R2: 289. But you also only tested 2 levels of litter. How well these levels correspond to real world conditons as well as the conditions tested by those other studies is an important consideration.
A: True, but this does not change the validity of the statement. How levels correspond to natural conditions was already described earlier (line 155-159).

R2: 290. You did not test for autotoxicity and cannot directly support this claim. There are other factors that could explain the presented trends.
A: We do not quite understand this claim. We tested for autotoxicity, since we compared germination and establishment of seeds of a species within its litter and control litter. Other mechanisms than autotoxicity are explicitly referred to in the discussion including a change in light, temperature, and water regime (line 331ff, 387ff, 429ff).

R2: 296. Given that litter did not consistently cover the soil surface, does that mean that using mass per area is actually a fair method for comparing litter types? Type is confounded by cover (even if mass per area is constant).
A: Type will always be confounded by cover. Nevertheless, using mass is still a valid approach. But since we were aware of these difference in cover caused by the same amount of different litter, we discussed this (line 333ff).
R2 304. which grass litter? Be specific.
A: Alopecurus pratensis was added.

R2: 308. But ragwort had lower germination than controls, which had no litter at all. So, even though ragwort decayed faster than grass litter, it should still have a neutral-positive impact relative to controls.
A: This is true. Therefore, we assume that litter leachate from ragwort also negatively influences germination and added this point subsequently.

R2: 322. Not exactly. Even if grasses and ragwort interacted, we would generally expect an intermediate response. What matters is if that response differs from the average of grasses and ragwort (assuming a 1:1 mixture grass:ragwort). The available data could easily test this.
A: We adjusted our analysis and added information on whether effects were additive or not. To this end, we used contrasts to specifically test whether the effect of mixed litter on response variables was mid-way between that of grass and ragwort alone (additive effect of pure type litters in the mixture).

R2: 326. That depends on the cover of ragwort (and on whether there was a mixing effect). If there is a perfect mixture of grasses and ragwort, your results suggest a

	neutral effect. If ragwort is abundant, then there would be a negative impact of leaving litter on ragwort germination.<br>A: This is true. But even if germination is hampered, seedling establishment is not. Thus, the net effect is not clear. We stated this in our conclusions.<br>R2: Also, is ragwort litter commonly removed by burning or raking?<br>A: No, as this is very laborious, ragwort litter is usually left on the grasslands.<br>R2: It is important to note that litter has different physical and chemical properties than green biomass, so this isn't exactly comparable to land manager mowing and leaving the trimmings in field.<br>A: This depends on the management. As we work with an authority, that wants to reduce insect damage by mowing, a bar-mower instead of a mulcher is used. This machine is comparable to a scythe. Therefore, the resulting "real world" litter is comparable to our litter. Furthermore, we expect the basic patterns for green biomass and litter to be comparable and can be used as a first approach to find an answer to topic.<br><br>R2: 341. If the biomass result is primarily a consequence of competition, is it fair to ascribe it to the litter type in the first place?<br>A: Yes, since litter type does influence seedling density in the first place.<br>R2: 349. Yes, but it takes time for these nutrients to be converted into forms usable by plants<br>A: We added this point.<br><br>R2: 414. See comments above – this is not a robust conclusion.<br>A: We tried to put our conclusions in perspective to make them more robust.<br><br>R2: Fig 1. Increase axis label size. Why are groups using upper case letters here and lower case letters in Fig 2? Unify if not intentional.<br>A: Lower case letters were used when there is an interaction like in Fig. 2. Upper cased letters are used for main effects. We added this information in the Figure descriptions.<br><br>R2: Fig 2. Add legend to figure. Increase axis label size.<br>A: Explanations are given in the figure caption.<br><br>R2: Fig 3. Increase axis label size.<br>A: We increased labels.<br>R2: Figs 1,3,4 – It would be helpful to have all of these presented in the format of Fig 3 with type, amount, and soil presented in Fig 1 and 4 as well. Even though soil was not significant in figs 1 and 4, it would provide consistency across a somewhat complex set of analyses and would make comparisons easier for the reader.<br>A: Figures are presented in the same format when no interaction of litter type and amount occurred. Soil is always included in the control displayed in all figures.
Additional Information:	
Question	Response
**Financial Disclosure**<br><br>Enter a financial disclosure statement that describes the sources of funding for the work included in this submission. Review the submission guidelines for detailed requirements. View published research articles from *PLOS ONE* for specific examples.<br><br>This statement is required for submission and **will appear in the published article** if the submission is accepted. Please make sure it is accurate.	This work was supported by the Stiftung Naturschutz Schleswig-Holstein (http://www.stiftungsland.de). We acknowledge financial support by Land Schleswig-Holstein within the funding programme Open Access Publikationsfonds (https://www.ub.uni-kiel.de/de/publizieren/open-access/uebernahme-von-open-access-publikationsgebuehren).<br>The funders had no role in study design, data collection and analysis, decision to publish, or preparation of the manuscript.

Powered by Editorial Manager® and ProduXion Manager® from Aries Systems Corporation

**Unfunded studies**<br>Enter: *The author(s) received no specific funding for this work.*<br><br>**Funded studies**<br>Enter a statement with the following details:<br>• Initials of the authors who received each award<br>• Grant numbers awarded to each author<br>• The full name of each funder<br>• URL of each funder website<br>• Did the sponsors or funders play any role in the study design, data collection and analysis, decision to publish, or preparation of the manuscript?<br>• **NO** - Include this sentence at the end of your statement: *The funders had no role in study design, data collection and analysis, decision to publish, or preparation of the manuscript.*<br>• **YES** - Specify the role(s) played.<br><br>* typeset	
**Competing Interests**<br><br>Use the instructions below to enter a competing interest statement for this submission. On behalf of all authors, disclose any competing interests that could be perceived to bias this work—acknowledging all financial support and any other relevant financial or non-financial competing interests.<br><br>This statement **will appear in the published article** if the submission is accepted. Please make sure it is accurate. View published research articles from *PLOS ONE* for specific examples.	The authors have declared that no competing interests exist.

**NO authors have competing interests**<br><br>Enter: *The authors have declared that no competing interests exist.*<br><br>**Authors with competing interests**<br><br>Enter competing interest details beginning with this statement:<br><br>*I have read the journal's policy and the authors of this manuscript have the following competing interests: [insert competing interests here]*<br><br>* typeset	
**Ethics Statement**<br><br>Enter an ethics statement for this submission. This statement is required if the study involved:<br><br>• Human participants<br>• Human specimens or tissue<br>• Vertebrate animals or cephalopods<br>• Vertebrate embryos or tissues<br>• Field research<br><br>Write "N/A" if the submission does not require an ethics statement.<br><br>General guidance is provided below. Consult the submission guidelines for detailed instructions. **Make sure that all information entered here is included in the Methods section of the manuscript.**	N/A

Powered by Editorial Manager® and ProduXion Manager® from Aries Systems Corporation

**Format for specific study types**<br><br>**Human Subject Research (involving human participants and/or tissue)**<br>• Give the name of the institutional review board or ethics committee that approved the study<br>• Include the approval number and/or a statement indicating approval of this research<br>• Indicate the form of consent obtained (written/oral) or the reason that consent was not obtained (e.g. the data were analyzed anonymously)<br><br>**Animal Research (involving vertebrate animals, embryos or tissues)**<br>• Provide the name of the Institutional Animal Care and Use Committee (IACUC) or other relevant ethics board that reviewed the study protocol, and indicate whether they approved this research or granted a formal waiver of ethical approval<br>• Include an approval number if one was obtained<br>• If the study involved *non-human primates*, add *additional details* about animal welfare and steps taken to ameliorate suffering<br>• If anesthesia, euthanasia, or any kind of animal sacrifice is part of the study, include briefly which substances and/or methods were applied<br><br>**Field Research**<br><br>Include the following details if this study involves the collection of plant, animal, or other materials from a natural setting:<br>• Field permit number<br>• Name of the institution or relevant body that granted permission	
**Data Availability**<br><br>Authors are required to make all data underlying the findings described fully available, without restriction, and from the time of publication. PLOS allows rare exceptions to address legal and ethical concerns. See the PLOS Data Policy and FAQ for detailed information.	Yes - all data are fully available without restriction

A Data Availability Statement describing where the data can be found is required at submission. Your answers to this question constitute the Data Availability Statement and **will be published in the article**, if accepted.<br><br>**Important:** Stating 'data available on request from the author' is not sufficient. If your data are only available upon request, select 'No' for the first question and explain your exceptional situation in the text box.<br><br>Do the authors confirm that all data underlying the findings described in their manuscript are fully available without restriction?	
**Describe where the data may be found in full sentences. If you are copying our sample text, replace any instances of XXX with the appropriate details.**<br><br>• If the data are **held or will be held in a public repository**, include URLs, accession numbers or DOIs. If this information will only be available after acceptance, indicate this by ticking the box below. For example: *All XXX files are available from the XXX database (accession number(s) XXX, XXX.*).<br>• If the data are all contained **within the manuscript and/or Supporting Information files**, enter the following: *All relevant data are within the manuscript and its Supporting Information files.*<br>• If neither of these applies but you are able to provide **details of access elsewhere**, with or without limitations, please do so. For example:<br><br>*Data cannot be shared publicly because of [XXX]. Data are available from the XXX Institutional Data Access / Ethics Committee (contact via XXX) for researchers who meet the criteria for access to confidential data.*<br><br>*The data underlying the results presented in the study are available from (include the name of the third party*	All relevant data are within the manuscript and its Supporting Information files.

and contact information or URL).<br>• This text is appropriate if the data are owned by a third party and authors do not have permission to share the data.<br><br>* typeset	
Additional data availability information:	

Powered by Editorial Manager® and ProduXion Manager® from Aries Systems Corporation

Manuscript Click here to access/download;Manuscript;Manuscript_Moehler_litter_effect

Conspecific and heterospecific litter effects on seedling emergence and growth in ragwort (Jacobaea vulgaris)

Henrike Möhler[1] *, Tim Diekötter[1], Geeltje Bauer[1], Tobias W. Donath[1]

[1]*Department of Landscape Ecology, Institute for Natural Resource Conservation, Kiel University, Germany*

Henrike Möhler hmoehler@ecology.uni-kiel.de +49-431-880-1007

ORCiD 0000-0001-8070-3889

Tim Diekötter tdiekoetter@ecology.uni-kiel.de +49-431-880-1212

ORCiD 0000-0003-4838-793X

Geeltje M. Bauer geeltje.bauer@googlemail.com

Tobias W. Donath tdonath@ecology.uni-kiel.de +049-431-880 1198

ORCiD 0000-0003-1874-1096

*Corresponding author

hmoehler@ecology.uni-kiel.de

Conspecific and heterospecific litter effects on seedling emergence and growth in ragwort (*Jacobaea vulgaris*)

Abstract

Jacobaea vulgaris Gaertn. or common ragwort is a widespread noxious grassland weed that is subject to different regulation measures worldwide. Seedling emergence and growth are the most crucial stages for most plants during their life cycle. Therefore, heterospecific or conspecific litter as well as soil-mediated effects may be of relevance for ragwort control. Our study examines the effects of conspecific and heterospecific litter as well as ragwort conditioned soil on seedling emergence and growth.

We conducted pot experiments to estimate the influence of soil conditioning (with, without ragwort), litter type (grass, ragwort, grass-ragwort-mix) and amount (200 g/m², 400 g/m²) on *J. vulgaris* recruitment. As response parameters, we assessed seedling number, biomass, height and number of seedling leaves.

We found that 200 g/m² grass litter led to higher seedling numbers, while litter composed of *J. vulgaris* reduced seedling emergence. Litter amounts of 400 g/m² had negative effects on the number of seedlings regardless of the litter type. Results for biomass, plant height and leaf number showed opposing patterns to seedling numbers. Seedlings in pots treated with high litter amounts and seedlings in ragwort litter became heavier, grew higher and had more leaves. Significant effects of the soil conditioned by ragwort on seedling emergence and growth were negligible.

The study confirms that the amount and composition of litter strongly affect seedling emergence and growth of *J. vulgaris*. Moreover, while conspecific litter and high litter amounts negatively affected early seedling development in ragwort, those seedlings that survived accumulated more biomass and got taller than seedlings grown in heterospecific or less dense litter. Therefore,

ragwort litter has negative effects in ragwort germination, but positive effects in ragwort growth.

Keywords: *Senecio jacobaea*, mulching, litter, autotoxicity, ragwort, phytotoxicity

Introduction

Jacobaea vulgaris Gaertn. or common ragwort is a widespread noxious grassland weed that is native to Europe, Asia, and Siberia and invasive in North America, New Zealand, and Australia. Since the pyrrolizidine alkaloids of the plant pose a health risk to cattle that consume this plant (1), its occurrence in high densities is a challenge for grassland management. Consequently, control of common ragwort is prescribed in the UK: ‘‘Weeds Act 1959’’ and ‘‘Ragwort Control Act 2003’’, Ireland: ‘‘Noxious Weed Act 1936’’, New Zealand: ‘‘Noxious Plant Act 1978’’, Australia: ‘‘Weed Management Act 1999’’, and Friesland, province of the Netherlands ‘‘Verordening Jakobskruiskruid 2007’’(2). Therefore, several studies have aimed to find effective and efficient ways to manage this grassland weed (2–5). Some of these studies came to the conclusion that specific cutting regimes are a promising approach (6). However, the toxicity of ragwort biomass precludes its use as hay or silage and the high number of long-living seeds rules out composting ragwort biomass (7, 8). Consequently, the biomass is often disposed of as special waste, which is costly (9). Yet, the occurrence of conspecific allelopathic effects (10, 11) suggests that leaving ragwort litter on mown grassland could be an additional component in managing ragwort abundance. As a first step to evaluate the validity of this approach, we experimentally tested this management option.

Litter influences micro-environmental conditions, is important for nutrient cycling and can have prominent effects on seedling germination and growth via mechanical and chemical effects (12, 13). Litter has been shown to have both positive and negative effects on seedling germination and establishment (13, 14). Positive effects were due to higher soil humidity and a lower amplitude of diurnal temperature fluctuations beneath a low litter cover (15). Still the size of litter effects on seedling germination and establishment do also depend on the amount of litter present. If litter layer is too high, it reduces the amount of light that reaches the seeds, which reaching seeds, where seeds of ragwort depend on a certain light amount to germinate (16). In

addition, thick litter layers that preserve a relative high humidity can facilitate the spread of mould that may cause decay of seeds (17). Van der Mejden and van der Waals-Koi showed that ragwort seedlings need light for germination (16). If not enough light is reaching the seeds, dormancy will be induced. On the other side they also demonstrated that germination was facilitated under light sand cover compared to no cover. They found that seeds need a certain moisture to germinate. Short periods of frost or drought can induce dormancy even if favourable conditions are restored again. After germination seedlings can be hampered in their growth or even die if litter amounts are too high. Thus Van der Meijden and van der Waals-Koi found significantly less seedlings in pots with high vegetation cover (16). Litter may also exert chemical effects by nutrient immobilization, depletion of oxygen in the soil or toxicity of carbon dioxide produced by decomposers or by releasing phytotoxins (11, 15, 18). Through these mechanisms litter potentially influences community organization via delaying and reducing or facilitating seedling emergence or seedling growth (13, 19).

Litter type is another important factor that can influence whether seed germination and seedling establishment is facilitated or hampered (20). It changes conditions for germination and growth as chemical as well as physical structure depend on the characteristics of species (20). Grass litter, for example, might be less hampering than large-leafed forb litter due to a lower amount of phytotoxins and an easier to penetrate structure (18, 20). Under natural conditions, however, litter is not usually monospecific, e.g. in grasslands it consists of a mixture of forb and grass litter. The effects of litter from plant communities are driven by species-specific litter properties (21) and may either be purely additive as hypothesized in the mass-ratio-hypothesis (22) or non-additive, as litter types may interact. In this context, Gartner and Cardon (23) found that more than half of the litter mixtures they reviewed behaved in a non-additive way concerning factors such as mass loss, nutrient transfer or decomposer composition.

It is intuitive to hypothesize that conspecific litter facilitates the establishment of conspecific seeds. Yet, as intraspecific competition was found to be more intense than interspecific competition (24, 25), inhibiting effects may also be expected. While many studies have shown facilitating effects of adult plants on conspecific seedlings (26–28), particularly in weed species (29–31), there are also studies demonstrating the opposite (32, 33). Some authors did not find evidence of unidirectional conspecific facilitation (34, 35). In line with the latter, studies on ragwort showed the negative autotoxic potential of ragwort litter and soil close from ragwort plants (36; 11). Also, Bezemer et al. 2006 and Van de Voorde et al. 2011 found a negative intraspecific plant-soil-feedback for ragwort, which they assumed is probably driven by pathogens that accumulate in the soil community (37, 38). Under these circumstances, negative effects of ragwort litter and ragwort conditioned soil on ragwort performance (e.g. lower biomass) may either be additive, i.e. result in stronger inhibitory effects than they would do individually, or the combined effect is not additive, i.e. combined effects are lower or higher than the sum of both effect sizes. However, the influence of litter during seedling emergence might be stronger than during seedling growth, since at the latter stage impacts of competition are of higher importance than direct litter effects (39). Thus, if litter reduces seedling density through self-thinning, surviving seedlings might have an advantage later on, as intraspecific competition would be reduced (40).

The present study is to our knowledge the first to test the influence of the combined effects of litter type, amount and soil conditioning on seedling emergence and growth in ragwort. To this end, we set up a common garden experiment, where we assessed the conspecific and heterospecific effects of litter on the number of emerged ragwort seedlings, their biomass, height, leaf number and specific leaf area. We posed the following questions:

I. Does litter type influence the emergence and growth of ragwort seedlings?

Assuming conspecific negative effects, we expect lower numbers of ragwort seedlings to emerge from beneath conspecific litter compared to pure grass litter or a mixture of grass and ragwort litter. We expect similar patterns to occur in seedling growth after emergence.

II. Does litter amount influence seedling emergence and growth of ragwort and does this depend on litter type?

We expect negative litter effects to increase with a higher litter cover and to be stronger with increasing litter amount. We also expect the size of litter effects to increase with the portion of ragwort litter in the litter cover. Thus, we expect the interaction of litter type and amount on seedling emergence and growth to be lowest in high amounts of ragwort litter. Seedling performance is hypothesized to be higher under medium ragwort amounts and highest under low amounts of grass litter.

III. Does ragwort-conditioned soil influence conspecific seedling emergence and growth? If so, do these effects interact with the litter effects?

We hypothesized an inhibitory effect of ragwort-conditioned soil on its own emergence and growth as negative plant-soil feedback for this species has been observed. We expect these effects to be additive for ragwort litter.

Material and methods

Study species

Jacobaea vulgaris L. syn. *Senecio jacobaea* Gaertn. or common ragwort is a herbaceous monocarpic plant from the Asteraceae family (41). It is a hemicryptophyte, forming a rosette in the first year and flowering in the second year (41). It prefers open patches and disturbed sites. Thus, it occurs on ruderal sites, along roadsides, pastures and grassland ecosystems in most

temperate regions of the world. It is often regarded as a noxious weed due to its content of toxic pyrrolizidine alkaloids (2). Ragwort shows ecological characteristics of a pioneer species, in particular its numerous light seeds and fast growth enable ragwort to spread rapidly in open areas (1). Each plant produces several thousand seeds per year (1). Seeds are mainly dispersed by wind over a distance of usually less than 15 m (42). Seed survival (up to 20 years) and germination rates (about 80%) are high (43).

Experimental design

We studied the effects of *litter amount* ($k = 2$; 2 (low) vs. 4 (high) g/dm^2), *litter type* ($k = 3$; ragwort litter, grass litter, 1:1 mix of ragwort and grass litter) and *conditioned soil* ($k = 2$; soil conditioned with ragwort vs. unconditioned soil that is soil **not** conditioned by ragwort) on seedling emergence and growth. The litter amounts applied correspond to the range of average productivity of extensively managed grasslands in Northern Germany (44). Loydi et al. (11) found positive litter effects to occur at these litter amounts. Thus, the litter amounts applied represent litter quantities that ragwort seeds are exposed to naturally and that seem to affect germination.

Seeds of *J. vulgaris* were hand-collected from at least 20 plant individuals on each of eight extensively managed pastures across Schleswig-Holstein from August to September 2015 and mixed for the experiment. These seeds were dry stored in darkness at room temperature until sowing on 21st April 2016. An initial viability test with a 1% tetrazolium chloride solution (45) on additional seed batches showed that most seeds (> 80%) were viable. *J. vulgaris* litter and conditioned and unconditioned soil was collected from rosettes on a ragwort-infested extensive cattle pasture near Preetz, Germany (54.227175, 10.242809) in April 2016. We assumed soil to be affected by ragwort, i.e. conditioned, if at least four ragwort-plants were growing within 0.5 m^2. We refer to unconditioned soil when soil was taken from the center of an area (diameter 10

m) where no ragwort was growing. Litter was collected, cleaned and dried for two days at room temperature and for 24 hours at 40 °C to constant weight. This way we ensured that chemicals potentially affecting germination were not changed. For grass litter, we used dried plant material from a grassland mainly consisting of sweet grass (Poaceae) species.

We set up a completely randomized experimental design. We prepared pots of 1 dm^3 volume (c. 10 × 10 × 10 cm) for each litter amount × litter type × soil conditioned (12 combinations) replicated eight times. To assess for seedlings emerging from the soil taken from ragwort stands we additionally included 20 control pots, ten with ragwort conditioned soil and ten filled with unconditioned soil. In none of these pots ragwort seedlings emerged. Half of the pots were filled with a 6:1 mixture of commercial potting soil (Fruhstorfer Erde®, Type P, Industrie-Erdenwerke Archut GmbH, Germany) and ragwort-conditioned soil or unconditioned soil, respectively. We followed the protocol of van de Voorde et al. (46) concerning inoculation and mixing of soil, as it led to significant differences between conditioned and unconditioned soil in their experiment.

In late April 2016, we sowed 50 seeds in each experimental pot and added litter on top of it. Pots were placed randomly in a common garden in Kiel, Germany (54.346883, 10.106829), surrounded by another row of pots (to reduce desiccation) and protected by a light metal grid to avoid litter being blown away. Pots were kept constantly moist by regular watering.

Germination started in mid May 2016. Seedlings that emerged above the litter cover were counted weekly until the tenth week after the experiment started. After the tenth week, we counted the total number of leaves per plant and measured the height of the highest plant in the pot. We determined specific leaf area for five pots per combination by cutting the youngest fully developed leaves of three plants. These were scanned and leaf area in mm^2 measured by the programme IMAGEJ (47). Afterwards leaves were dried at 60 °C and weighed to the closest milligram. The same was done with total aboveground biomass.

Data analysis

We tested for differences in number of emerged seedlings and measurements of seedling growth represented by: total biomass per pot and per plant, plant height, leaf number and specific leaf area. We used a three-factorial ANOVA to analyze the effect of litter type, litter amount and soil type, and their interactions. Residuals were examined visually to assess validity of assumptions for analysis of variance, i.e. normal distribution and variance homogeneity. Data for biomass per pot and biomass per plant had to be ln transformed to meet assumptions of analysis of variance. Subsequent to significant ANOVA results, post-hoc Tukey-HSD test was employed to test for individual differences. In addition, we used contrasts to specifically test whether the effect of mixed litter on response variables was mid-way between that of grass and ragwort alone (additive effect of pure type litters in the mixture). Since not general litter effects but specific effects of the different litter types on seedling emergence and growth of ragwort were the focus in our study, we did not include the controls in our model but included them for visual comparison in the figures. In addition, we analyzed the controls for soil effects as well as for differences to the litter types across litter amount using contrasts. As a measure for the relative contribution of each factor and their interactions to the total variability of the response parameters of interest, we used the ratio of the sum of squares of the factor or interaction of interest to the total sum of squares (i.e. for all factors, their interactions and error). All analyses were done using R version 3.6.1 (48).

Results

In general, our results showed significant main effects of litter type and litter amount on all response variables: seedling number, biomass per plant and pot, plant height and mean leaf number. In contrast, the soil factor (conditioned vs. unconditioned) was not significant for any response variables under the litter treatments. Only the case of the separate analysis of controls

soil revealed a significant effect on plant height. Our analyses revealed two significant interactions between main factors. In the case of biomass per plant and pot and seedling height we found a significant two-way *litter type* × *litter amount* interaction. Effects of the treatments on specific leaf area were not significant (results not shown).

Seedling emergence

We found that while both, litter type and litter amount, affected seedling emergence significantly, the amount of explained variance was more than twice as high for litter type than litter amount (38.22 % vs. 16.58 %; Table 1). Number of emerged seedlings differed significantly between all three litter types (Fig. 1). In addition, contrast analyses of the control against litter types revealed that the litter mixture had no significant effect on seedling number compared to the control ($p = 0.30$), while in the control seedling number differed significantly from grass and ragwort litter ($p = 0.016$ and $p \leq 0.0001$, respectively). From beneath grass litter, seedling emergence was significantly raised by about 50% compared to ragwort litter (18.1 ± 1.0 vs. 27.6 ± 0.8), while emergence from beneath mixed litter was intermediate (22.8 ± 0.8) (Fig. 1). Litter effects were additive, i.e. the effect size of mixed grass-ragwort litter did not deviate significantly from the expected mean effect of pure grass and ragwort litter ($p = 0.99$). Across litter types higher litter amounts of 400 g/m², significantly reduced seedling numbers by about one fifth, compared to low litter cover of 200 g/m² (24.2 ± 1.0 vs. 20± 1) (Fig 1).

Table 1. Results of a three-way ANOVA on the effects of litter type, litter amount and soil on seedling number and measures of seedling growth (biomass per plant, plant height and mean leaf number).

		Seedling number				Ln Biomass per pot				Height				Mean leaf number			
**Source of variation**	df	MSQ	F	*P*	vc	MSQ	F	*P*	vc	MSQ	F	*P*	vc	MSQ	F	*P*	vc
**Litter type**	2	**726.8**	**38.107**	**≤ 0.0001**	**38.22**	**10.824**	**95.940**	**≤ 0.0001**	**55.23**	**234.63**	98.618	**≤ 0.0001**	**56.51**	**1.602**	**7.868**	**≤ 0.0001**	**11.64**
**Litter amount**	1	**630.4**	**33.053**	**≤ 0.0001**	**16.58**	**12.355**	**5.415**	**≤ 0.0001**	**13.82**	**105.84**	44.486	**≤ 0.0001**	**12.74**	**5.505**	**27.037**	**≤ 0.0001**	**20.02**
**Soil**	1	42.7	2.237	0.138	1.12	0.008	0.072	0.427	0.18	0.07	0.030	0.864	0.01	0.108	0.532	0.468	0.40
**Litter type x litter amount**	2	52.3	1.371	0.259	1.38	**1.252**	**1.127**	**≤ 0.0001**	**5.75**	**21.84**	**9.181**	**≤ 0.0001**	**5.26**	0.542	2.663	0.076	3.94
**Litter type x soil**	2	21.3	0.558	0.575	0.56	0.043	0.125	0.333	0.64	0.14	0.059	0.943	0.04	0.104	0.512	0.601	0.75
**Litter amount x soil**	1	1.0	0.055	0.816	0.03	0.004	0.001	0.924	0.00	3.15	1.326	0.253	0.40	0.012	0.059	0.809	0.04
**Litter type x litter amount x soil**	2	0.1	0.1	0.004	0.00	0.049	0.038	0.336	0.19	3.99	1.679	0.192	0.96	0.139	0.684	0.507	1.01
**Error**	84	1602.0			42.12	0.104			24.18	2.38			24.07	0.204			62.18

d.f. = degrees of freedom; MS = mean sum of squares; vc (%) = relative contribution of individual factors and their interactions to total variation.

Significant effects (*P* < 0.05) are given in bold.

Fig 1. Seedling number: effects of soil conditioning on the control, litter type (grass, mix, ragwort (left)) and different litter amounts (200 g/m² litter and 400 g/m² (right)). In the box plots middle lines represent median, boxes represent the first and third quartiles, lower and upper bars represent the minimum and the maximum and points represent outliers (i.e. points above 1.5 SD). Upper case letters indicate significant main effects between treatments (TukeyHSD, $P \leq 0.05$).

Seedling growth

More than half of the variance in biomass per pot was explained by litter type, whereas litter amount only explained around one tenth of the variance (Table 1). The significant interaction, which accounted only for 6% of the variance, was caused by increased biomass with increasing litter amount when litter contained ragwort compared to no effect of amount in grass litter (Table 1; Fig. 2). Across litter amounts biomass per pot was more than doubled for seedlings that grew in ragwort litter compared to those in grass litter (1.1 ± 0.1 g vs. 0.4 ± 0.1 g) (Fig. 2). Interestingly, seedling biomass per pot in the litter mixture (0.9 ± 0.1 g) lay between that of seedlings growing in pure grass or ragwort litter but in contrast to seedling emergence, the litter type effects were not additive, i.e. seedling biomass per pot in the mixed litter treatment lay significantly above the mean of seedling biomass in grass and ragwort litter ($p = 0.001$, Fig. 2). Across litter types, biomass per plant and per pot increased with increasing litter amount applied (0.56 ± 0.03 g in low litter vs. 1.00 ± 0.08 g in high litter). Contrast analyses of the control against the three litter types revealed no significant difference of biomass per pot between the control and grass litter ($p = 0.40$) but significant differences in biomass between control and litter mixture as well as ragwort litter (both $p \leq 0.0001$). Seedling biomass per plant showed almost the same response patterns but the litter effect was additive ($p = 0.88$; Supporting information Fig S1).

Fig 2. Biomass per pot - effects of soil conditioning on the control and the two-way interaction of litter type and litter amount. In the box plots middle lines represent median, boxes represent the first and third quartiles, lower and upper bars represent the minimum and the maximum and points represent outliers (i.e. points above 1.5 SD). Grey: 200 g/m^2 litter, black: 400 g/m^2 litter. Lower case letters indicate significant interactions between treatments (TukeyHSD, $P \leq 0.05$).

Seedling height was largest for seedlings growing in high amounts of ragwort litter (Fig. 3). Across amounts seedlings in ragwort litter grew almost three times as tall as in grass litter (8.1 ± 2.8 cm vs. 2.8 ± 0.9 cm) and about one third taller compared to the litter mixture (6.5 ± 0.3 cm). Plant height was significantly influenced by the main factors litter type and litter amount as well by the interaction of these factors (Fig 3). More than half of the variance was explained by litter type (Table 1). Again litter type effects were not additive, i.e. seedling height per pot in the mixed litter treatment lay significantly above the mean of seedling height in grass and ragwort litter ($p = 0.001$, Fig. 3). Contrast analyses of the control against the three litter types revealed no significant difference of height between the control and grass litter ($p = 0.39$) but significant differences between control and litter mixture as well as ragwort litter (both $p \leq 0.0001$). In contrast, litter amount had a much smaller, still significant effect, i.e. higher litter amounts led to taller seedlings (Fig. 3). Seedling height is the only response variable where the control was significantly influenced by soil type, i.e. ragwort conditioned soil led to significantly taller seedlings (3.9 ± 0.5 cm vs. 2.6 ± 0.3 cm).

Fig 3. Seedling height - effects of soil conditioning on the control and the two-way interaction of litter type and litter amount. In the box plots middle lines represent median, boxes represent the first and third quartiles, lower and upper bars represent the minimum and the maximum and points represent outliers (i.e. points above 1.5 SD). Grey: 200 g/m² litter, black: 400 g/m² litter. Lower case letters indicate significant interactions between treatments (TukeyHSD, $P \leq 0.05$).

The mean leaf number of plants growing in ragwort litter was highest compared to in plants growing in grass litter and the combination of both litter types (ragwort 3.59 ± 0.12, grass 3.15 ± 0.05, mixture 3.31 ± 0.08). Analyses of variance revealed that mean leaf number was significantly affected by litter type and amount but not their interaction (Table 1; Fig 4). The variance explained by litter amount was twice as high as that explained by litter type (Table 1). Thus, mean leaf number is the only measured variable that seems to be stronger influenced from litter amount than litter type. Effects of litter type were additive, i.e. the leaf number in the litter mixture did not differ significantly from the expected mean effect of pure grass and ragwort litter ($p = 0.49$, Fig. 4). Plants growing in high litter amounts (400 g/m²) had significantly more leaves per plant than plants growing in the low litter amounts (200 g/m²). Contrast analyses revealed no significant difference in leaf number between the plants growing in the control and in any of the three litter types ($p > 0.05$).

Fig 4. Mean leaf number under soil conditioning in the control, different litter types (grass, mix, ragwort (left)) and different litter amounts (200 g/m² litter, 400 g/m² litter (right)). In the box plots middle lines represent median, boxes represent the first and third quartiles, lower and upper bars represent the minimum and the maximum and points represent outliers (i.e. points above 1.5 SD). Upper case letters indicate significant main effects between treatments (TukeyHSD, $P \leq 0.05$).

Discussion

Our experimental results suggest that litter type as well as litter amount do influence seedling emergence and growth, whereas conditioned soil did not when combined with litter treatments. It is interesting to note that although ragwort litter reduced the number of conspecific emerging seeds, seedlings actually performed better in ragwort litter showing increased biomass, height and leaf numbers.

Litter type accounted for a much higher portion of the variance than litter amount. In contrast, other studies reported that litter amount was more relevant than litter type (49, 50). This highlights the species specific nature of litter effects (18). One explanation for this pattern could be that ragwort litter is indeed autotoxic and induces self-thinning by negatively influencing the emergence of conspecific seedlings (11).

Seedling emergence was highest in grass litter, lowest in ragwort litter and intermediate in the litter mixture. Germination was highest in medium compared to no or high litter amounts. In contrast, all parameters measuring seedling growth (biomass, height, mean leaf number) were highest in high and/or ragwort litter. The positive effect of grass litter and medium litter amounts on seedling emergence might be due to physical litter effects like preserving a relatively constant temperature and soil moisture (51). In our experiment, the same amount of grass litter covered a larger proportion of the soil surface than ragwort or mixed litter (high amount: grass 90 ± 3%, mixed 85 ± 4% and ragwort 67 ± 8%, low amount: grass: 85 ± 3%, mixed 70 ± 9%, ragwort 37 ± 5%). In accordance with a study by Eckstein and Donath (51), which revealed a rise in soil moisture when litter cover increased, we assume that seedlings experienced more favourable water availability in grass litter and medium litter amounts. In high litter amounts however, negative effects of litter prevailed over positive effects. Laboratory experiments of van der Meijden et al. (16) demonstrated that seed germination of ragwort increased with higher soil humidity. We also observed that ragwort litter decayed faster than

grass litter (personal observation). This is in line with general findings that litter of forb species such as ragwort tends to decay faster than grass litter (52). Schuster et al. (53) found that the mass of ragwort litter remaining after 30 days was only half of the amount of grass (*Alopecurus pratensis*) litter. Since the protective properties of a litter cover against desiccation and temperature extremes increases with litter amount present, seedlings emerging from beneath faster decaying ragwort litter might have experienced less protection than those seedlings emerging beneath grass litter (15, 51). Thus, the negative effect of ragwort litter on ragwort seedlings emergence seems to be a result of fewer physical benefits seedlings experienced from conspecific litter compared to grass litter. In addition, leachate effects may also depend on the specific decomposition rate of different litter types. As leachates released from the litter are declining very rapidly after senescence (54), intensity of seedlings' exposure to leachate depends on the decomposition rate of the litter present. Thus, ragwort seeds and seedlings emerging from ragwort litter, which decays fast (53), were only exposed to the ragwort leachate for a short time. Consequently, we assume that leachate effects on seedling emergence were more pronounced than on seedling growth.

In general, the amelioration of the physical environment by litter, rather than its chemical composition, seems to be the main factor boosting seedling growth in short time scales (21). However, the size of chemical autotoxic effects and therefore their relative importance in relation to physical and mechanical or other biotic effects are species-specific (55). Several studies show the occurrence of autotoxicity of ragwort (36; 11), which suggests that chemical effects are an additional mechanism for lower seedling emergence from beneath ragwort litter. Van de Voorde (36) reported decreasing germination with increasing concentrations of ragwort extract. As ragwort produces many seeds with high germination potential and low dispersal ability (43, 1, 42) autotoxic effects may avoid intraspecific competition in ragwort and thus may lead to lower seedling numbers but increased seedling vitality.

In contrast of Van de Voordes notion of a negative soil-feedback in ragwort (36), we found only weak indications of effects of ragwort conditioned soil on seedling emergence and growth (Table 1). In fact, the only significant effect of conditioned soil occurred on seedling height in controls, i.e. seedlings in conditioned soil grew bigger than in unconditioned soil. This finding is in contrast to findings of van de Voorde (32), who reported autotoxic effects of ragwort on its seedling development. However, on average the observed difference in plant height between ragwort seedlings growing in conditioned versus those growing in unconditioned soil including control and litter addition treatments was only 2 mm (less than 4 % of the average height of about 54.5 mm). Therefore, we expect that this difference is too small to be of ecological relevance. In other studies, looking at several seedling cohorts, those cohorts usually differed several centimeters in height (56–58).

Mixed litter resulted in fewer emerged seedlings than in grass litter, but more emerged seedlings compared to ragwort litter, which suggests that the effects of grass and ragwort litter simply added up to a neutral effect. This supports the mass-ratio hypothesis by Grime (22) for the factors seedling number, biomass per plant and mean leaf number, i.e. mixed litter effects did not deviate positively nor negatively from the expected combined effects. However, for biomass per pot and plant height, effects in mixed litter were higher than could be assumed from the simple addition of grass and ragwort. Thus, ragwort growth profited in single aspects from mixed litter layers. Thus, from a management point of view, leaving litter on ragwort infested sites does slightly facilitate ragwort growth when biomass of ragwort and grass is balanced. However, if the site is rather productive and ragwort is dominating the standing biomass, germination of its seedlings might indeed be limited. When litter cover is too high, negative effects prevail over positive effects. Accordingly, compared to the control treatment, seedling emergence was lowest from beneath 400 g of litter and not reduced from beneath 200 g of litter cover. The reduced seedling emergence of ragwort from beneath 400 g of litter is very likely

linked to the relatively small seed mass of ragwort (0.22 mg; (59)). The smaller the seeds are, the less resources a seed can spend to successfully penetrate through a litter cover (60). In addition, small seeded species, like ragwort, tend to depend on higher light levels for germination than large seeded species (61, 62). Since photosynthetic active radiation (PAR) decreases with increasing litter cover (51) not only will germination be lower, but germinated seedlings will also lack light resources for successful penetration of the litter cover. This is also in accordance with van der Meijden and van der Waals-Kooi (16), who found that emergence in ragwort significantly decreased when seeds were placed beneath a sand cover of more than 4 mm. Furthermore, Facelli et al. (63) showed that ruderal species, such as ragwort, tend to be more sensitive and thus more likely to be negatively affected by high amounts of litter. Therefore, ragwort seedlings might be reduced by a mulching regime on productive sites where litter amounts are medium to high.

In contrast to the findings for seedling emergence, seeds in ragwort litter built up more biomass, grew higher and tended to have more leaves than seedlings in grass litter. Biomass and height are the two variables where we found a significant interaction between litter type and litter amount. This could be explained by a reduction in competition for the few seedlings that emerged in ragwort litter. The interaction was quantitative and weak compared with the effects of litter type and amount alone (Table 1). Seedlings emerging from beneath high amounts of litter and/or ragwort litter built up the highest biomass and got tallest. This is also diametrical to the findings of van de Voorde et al. (36), who reported lower biomass for seedlings in ragwort influenced environments. These conflicting results could be due to different methodology. While van de Voorde et al. (36) harvested seedling biomass after 19 days, seedlings in our study were harvested about 50 days later. While van de Voorde's results were supported by our early observations (seedlings looked smaller and thinner early during early development in ragwort litter), seedlings started to grow bigger after ragwort litter had decomposed. Thus, the longer duration of our experiment compared to the experiment by van de Voorde et al. (35) allowed

us to observe a change in the importance of litter effects in relation to intraspecific density dependent competitive effects (49), which is further supported by the fact that lower seedling numbers from beneath grass litter resulted in seedlings of higher biomass and height. Loydi et al. (49) already identified, that while litter plays a major role for seedling emergence, for instance, via phytotoxins, competition is more important for seedling development later during seedling growth. Significantly increased mean leaf numbers in seedlings in high litter amounts and in ragwort litter compared to plants in grass or mixed litter might be linked to lower intraspecific competition as there were fewer seedling numbers in the ragwort pots or by less physical hampering compared to grass litter (60). However, indirect positive effects of reduced seedling emergence in boosting seedling biomass could be especially pronounced in situations where, as in the present experiment, intraspecific competition prevails and may overlay different environmental constrains under field conditions (13, 64).

Another explanation for superior performance of emerged seedlings in high litter amounts and ragwort litter might be an improved availability of nutrients (12), as more biomass contains more nutrients and ragwort litter decomposed quite fast. This is supported by our observation that even though the number of seedlings in the control pot and in the ragwort litter pot with 200 g/m^2 was about the same and thus competition among seedlings was comparable, biomass of seedlings growing in ragwort litter pots was still higher. However, it is questionable if nutrients from decaying litter are converted to plant available nutrients within 70 days. Effects of different litter amounts on nutrient availability are generally relatively low (49). Another reason for lighter and smaller seedlings in lower amounts of litter could be due to the missing investment of seedlings in higher growth when they have already reached light (65). Siebenkäs et al. (66) compared seedling growth in the same developmental stage as seedlings in our experiment and found that plants tend to grow higher when there is more shade. Thus, seedlings may grow higher in higher litter amounts to avoid its shade and in order to reach light. Renne et al. (2006) found that small differences in height of seedlings early on can produce large

differences in their final biomass (67) indicating that early shading can translate in higher heights, even if shade is gone.

Conclusion

We found both negative and positive effects of ragwort litter on conspecific seedling emergence and growth. While negative effects can be most likely linked to autotoxic chemical and physical ragwort litter effects in the stage of seedling emergence, those seedlings that emerged successfully may have profited from a lower intraspecific competition and may had more resources to invest than seedlings that emerged beneath grass or mixed litter. Due to the ambivalent nature of our results, these findings cannot easily be translated into management recommendations for ragwort-infested sites. However, the effects of mixed litter on ragwort recruitment were either neutral or even promoted ragwort growth. Therefore, when we assume that biomass on mowed ragwort pastures consists roughly of similar amounts of grass and ragwort, we expect that leaving litter containing ragwort biomass on the grassland will have no negative effect on ragwort population density or growth. As already our results for a simple controlled experiment do not lead to a clear hint of the effectiveness of ragwort litter in reducing ragwort population density, we do not expect pronounced effects in an experiment under more natural conditions.

Acknowledgements

We thank Astrid Dempfle for her support in statistical analyses. Furthermore, we thank Hollyn Hartlep for her valuable help in proofreading. The research in this article was funded by the Stiftung-Naturschutz Schleswig-Holstein.

Literature Cited

1. Wardle DA. The ecology of ragwort (*Senecio jacobaea* L.): A review. New Zealand Journal of Ecology 1987 [cited 2016 Feb 25]; 10:67–76.

2. Leiss KA. Management practices for control of ragwort species. Phytochemistry Reviews 2011; 10(1):153–63.

3. McEvoy PB, Cox C, Coombs E. Successful biological control of ragwort, *Senecio jacobaea*, by introduced insects in Oregon. Ecological Applications 1991; 1(4):430–42.

4. Suter M, Lüscher A. Measures for the control of *Senecio aquaticus* in managed grassland. Weed Research 2011 [cited 2016 Apr 27]; 51(6):601–11.

5. Jacobs J, Sing S. Ecology and management of tansy ragwort (*Senecio jacobaea* L.). Montana: Natural Resources Conservation Service; 2009.

6. Siegrist-Maag S, Lüscher A, Suter M. Reaktion von Jakobs-Kreuzkraut auf Schnitt. Agrarforschung (Switzerland) 2008; 15(7):338–43.

7. Kaltner F, Rychlik M, Gareis M, Gottschalk C. Influence of storage on the stability of toxic pyrrolizidine alkaloids and their N-Oxides in peppermint tea, hay, and honey. J Agric Food Chem 2018; 66(20):5221–8. Available from: URL: https://doi.org/10.1021/acs.jafc.7b06036.

8. Neumann H, Lütt S, Schleich-Saidfar C, Rabe I, Walter A, Böhling J et al. Umgang mit dem Jakobs-Kreuzkraut: Meiden - Dulden - Bekämpfen. Flintbek: LLUR; 2009. (LLUR SH / Natur; vol 14) [cited 2016 Dec 12].

9. Huckauf A, Rabe I, Aboling S, Böhling J, Böttner E, Ehlers B et al. Umgang mit dem Jakobs-Kreuzkraut: Meiden - dulden - bekämpfen. 4. überarbeitete Auflage. Flintbek: Landesamt für Landwirtschaft, Umwelt und ländliche Räume des Landes Schleswig-Holstein (LLUR); 2017. (Schriftenreihe LLUR SH. Natur; vol 25) [cited 2018 Mar 19]. Available from: URL: https://www.stiftungsland.de/fileadmin/pdf/JKK/senecio.pdf.

10. Wang R, Yang X, Song Y, Zhang M, Linhu, Su Y et al. Allelopathic potential of Tephrosia vogelii Hook. f.: Laboratory and field evaluation. Allelopathy Journal 2011 [cited 2018 Apr 23]; 28(1):53–62. Available from: URL: https://www.researchgate.net/profile/Ruilong_Wang/publication/287677172_Allelopathic_potential_of_Tephrosia_vogelii_Hook_f_Laboratory_and_field_evaluation/links/58735d0308ae8fce492390aa/Allelopathic-potential-of-Tephrosia-vogelii-Hook-f-Laboratory-and-field-evaluation.pdf.

11. Möhler H, Diekötter T, Herrmann JD, Donath TW. Allelopathic vs. autotoxic potential of a grassland weed—evidence from a seed germination experiment. Plant Ecology & Diversity 2018 [cited 2020 Jul 15]; 11(4):539–49.

12. Facelli JM, Pickett STA. Plant litter: Its dynamics and effects on plant community structure. The Botanical Review 1991 [cited 2018 Apr 23]; 57(1):1–32. Available from: URL: https://link.springer.com/content/pdf/10.1007%2FBF02858763.pdf.

13. Loydi A, Eckstein RL, Otte A, Donath TW, Gómez-Aparicio L. Effects of litter on seedling establishment in natural and semi-natural grasslands: A meta-analysis. J Ecol 2013 [cited 2016 Apr 18]; 101(2):454–64.

14. Xiong S, Nilsson C. The effects of plant litter on vegetation: A meta-analysis. Journal of Ecology 1999 [cited 2018 Apr 23]; (87):984–94.

15. Loydi A, Lohse K, Otte A, Donath TW, Eckstein RL. Distribution and effects of tree leaf litter on vegetation composition and biomass in a forest–grassland ecotone. Journal of Plant Ecology 2014; 7(3):264–75.

16. van der Meijden E, van der Waals-Kooi RE. The population ecology of *Senecio jacobaea* in a sand dune system: I. Reproductive strategy and the biennial habit. Journal of Ecology 1979; 67(1):131–53.

17. Baskin CC, Baskin JM. Seeds: ecology, biogeography, and evolution of dormancy and germination. 2nd ed. Amsterdam: Elsevier; 2014.

18. Bonanomi G, Sicurezza MG, Caporaso S, Esposito A, Mazzoleni S. Phytotoxicity dynamics of decaying plant materials. New Phytol 2006 [cited 2016 Apr 11]; 169(3):571–8.

19. van der Putten WH, Bardgett RD, Bever JD, Bezemer TM, Casper BB, Fukami T et al. Plant-soil feedbacks: The past, the present and future challenges. J Ecol 2013 [cited 2016 Apr 6]; 101(2):265–76.

20. Donath TW, Eckstein RL. Grass and oak litter exert different effects on seedling emergence of herbaceous perennials from grasslands and woodlands. Journal of Ecology 2008; 96(2):272–80. Available from: URL: https://besjournals.onlinelibrary.wiley.com/doi/pdf/10.1111/j.1365-2745.2007.01338.x.

21. Quested H, Eriksson O. Litter species composition influences the performance of seedlings of grassland herbs. Functional Ecology 2006 [cited 2020 Apr 27]; 20:522–32.

22. Grime JP. Benefits of plant diversity to ecosystems: immediate, filter and founder effects. J Ecol 1998 [cited 2020 Apr 27]; 86:902–10.

23. Gartner TB, Cardon ZG. Decomposition dynamics in mixed-species leaf litter. Oikos 2004 [cited 2020 Apr 27]; 104:230–46.

24. Antonovics J, Levin DA. The ecological and genetic consequences of density-dependent regulation in plants. Ann. Rev. Ecol. Syst. 1980 [cited 2020 Apr 27]; 11(11):411–52.

25. Adler PB, Smull D, Beard KH, Choi RT, Furniss T, Kulmatiski A et al. Competition and coexistence in plant communities: Intraspecific competition is stronger than interspecific competition. Ecology Letters 2018; 21(9):1319–29. Available from: URL: https://onlinelibrary.wiley.com/doi/pdf/10.1111/ele.13098.

26. Pleasants JM, Jurik TW. Dispersion of seedlings of the prairie compass plant, *Silphium laciniatum* (Asteraceae). American Journal of Botany 1992; 79:133–7.

27. Roll J, Mitchell RJ, Cabin RJ, Marshall DL. Reproductive success increases with local density of conspecifics in a desert mustard (*Lesquerella fendleri*). Conservation Biology 1997 [cited 2020 Apr 27]; 11(11):738–46.

28. Montesinos D, García-Fayos P, Mateu I. Conflicting selective forces underlying seed dispersal in the endangered plant *Silene diclinis*. Int. J. Plant Sci. 2006 [cited 2020 Apr 27]; 167(1):103–10.

29. McEvoy PB. Seedling dispersion and the persistence of ragwort Senecio jacobaea (Compositae) in a grassland dominated by perennial species. Oikos 1984; Vol. 42(No. 2):138–43.

30. Wardle DA, Ahmed M, Nicholson KS. Allelopathic influence of nodding thistle (*Carduus nutans* L.) seeds on germination and radicle growth of pasture plants. New Zealand Journal of Agricultural Research 1991 [cited 2020 Apr 27]; 34(2):185–91.

31. Reinhart KO, Maestre FT, Callaway RM. Facilitation and inhibition of seedlings of an invasive tree (*Acer platanoides*) by different tree species in a mountain ecosystem. Biol Invasions 2006 [cited 2020 Apr 27]; 8(8):231–40.

32. Singh HP, Batish DR, Kohli RK. Autotoxicity: Concept, organisms, and ecological significance. Crit Rev Plant Sci 1999; 18:757–72.

33. Suzuki RO, Kudoh H, Kachi N. Spatial and temporal variations in mortality of the biennial plant, *Lysimachia rubida*: Effects of intraspecific competition and environmental heterogeneity. J Ecology 2003 [cited 2020 Apr 27]; 91:114–25.

34. Hovstad KA, Ohlson M. Conspecific versus heterospecific litter effects on seedling establishment. Plant Ecol 2009 [cited 2018 Apr 19]; 204(204):33–42. Available from: URL: https://link.springer.com/content/pdf/10.1007%2Fs11258-008-9563-2.pdf.

35. Campanella MV, Bisigato AJ. Conspecific leaf litter and root competition inhibits shrub emergence in the Patagonian steppe. Plant Ecol 2019; 220(10):985–93.

36. van de Voorde TFJ, Ruijten M, van der Putten WH, Bezemer TM. Can the negative plant–soil feedback of *Jacobaea vulgaris* be explained by autotoxicity? Basic and Applied Ecology 2012 [cited 2020 Apr 28]; 13(6):533–41.

37. Bezemer TM, Harvey JA, Kowalchuk GA, Kopershoek H, van der Putten WH. Interplay between *Senecio jacobaea* and plant, soil, and aboveground insect community composition. Ecology 2006; 87(8):2002–13.

38. van de Voorde TFJ, van der Putten WH, Martijn Bezemer T. Intra- and interspecific plant-soil interactions, soil legacies and priority effects during old-field succession. Journal of Ecology 2011; 99(4):945–53.

39. Loydi A, Donath TW, Eckstein RL, Otte A. Non-native species litter reduces germination and growth of resident forbs and grasses: allelopathic, osmotic or mechanical effects? Biol Invasions 2015 [cited 2016 Mar 31]; 17:581–95.

40. Begon M, Townsend CR, Harper JL. Ecology: From Individuals to Ecosystems. 4th ed.: Blackwell Publishing; 2011.

41. Harper JL, Wood WA. Biological Flora of the British Isles: *Senecio jacobaea*. Journal of Ecology 1957; 45(2):617–37.

42. McEvoy PB, Cox CS. Wind dispersal distances in dimorphic achenes of ragwort, *Senecio jacobaea*. Ecology 1987; 68(6):2006–15.

43. Crawley M, Nachapong M. The establishment of seedlings from primary and regrowth seeds of ragwort (*Senecio jacobaea*). Journal of Ecology 1985; 73(1):255–61.

44. Smit HJ, Metzger MJ, Ewert F. Spatial distribution of grassland productivity and land use in Europe. Agricultural Systems 2008 [cited 2020 Apr 27]; 98(98):208–19.

45. ISTA. Chapter 6: Biochemical test for viability: the topographical tetrazolium test. Int. rules seed test. 2015 [cited 2017 Oct 20]; 2015(1):6.

46. van de Voorde TFJ, van der Putten WH, Bezemer TM. The importance of plant-soil interactions, soil nutrients, and plant life history traits for the temporal dynamics of *Jacobaea vulgaris* in a chronosequence of old-fields. Oikos 2012 [cited 2016 Feb 9]; 121(8):1251–62.

47. Schindelin J, Rueden CT, Hiner MC, Eliceiri KW. The ImageJ ecosystem: An open platform for biomedical image analysis. Molecular Reproduction and Development 2015; 82(82):518–29.

48. R: A language and environment for statistical computing. Vienna, Austria: R Foundation for Statistical Computing; 2019. Available from: URL: https://www.R-project.org/.

49. Loydi A, Donath TW, Otte A, Eckstein RL. Negative and positive interactions among plants: Effects of competitors and litter on seedling emergence and growth of forest and grassland species. Plant Biol J 2015 [cited 2016 Apr 25]; 17(3):667–75.

50. Myster RW. Species-specific effects of grass litter mass and type on emergence of three tall grass prairie species. Ecoscience 2006; 13(13):95–9.

51. Eckstein RL, Donath TW. Interactions between litter and water availability affect seedling emergence in four familial pairs of floodplain species. Journal of Ecology 2005 [cited 2016 Apr 21]; 93(4):807–16.

52. Shaw RM, Harte J. Control of litter decomposition in a subalpine meadow–sagebrush steppe ecotone under climate change. Ecological Applications 2001 [cited 2020 Apr 27]; 11(4):1206–23.

53. Schuster MJ, Kreyling J, Berwaers S, Walter J, Boeck HJ de, Dengler J et al. Drought inhibits synergistic interactions of native and exotic litter mixtures during decomposition in temperate grasslands. Plant Soil 2017 [cited 2020 Apr 27]; 415(415):257–68.

54. Sydes C, Grime JP. Effects of tree leaf litter on herbaceous vegetation in deciduous woodland: II. An experimental investigation. The Journal of Ecology 1981; 69(1):249–62. Available from: URL: http://dx.doi.org/10.2307/2259829.

55. Smith-Ramesh LM, Reynolds HL. The next frontier of plant–soil feedback research: unraveling context dependence across biotic and abiotic gradients. Journal of Vegetation Science 2017 [cited 2020 Apr 27]; 28(28):484–94.

56. Seiwa K. Effects of seed size and emergence time on tree seedling establishment: importance of developmental constraints. Oecologia 2000 [cited 2020 Apr 27]; 123(123):208–15.

57. Pausas JG, Ouadah N, Ferran A, Gimeno T, Vallejo R. Fire severity and seedling establishment in Pinus halepensis woodlands, eastern Iberian Peninsula. Plant Ecol 2003 [cited 2020 Apr 27]; 169(169):205–13.

58. Quintana J, Cruz A, Fernández-González F, Moreno JM. Time of germination and establishment success after fire of three obligate seeders in a Mediterranean shrubland of central Spain. Journal of Biogeography 2004 [cited 2020 Apr 27]; 31(31):241–9.

59. Fenner M. Relationships between seed weight, ash content and seedling growth in twenty-four species of Compositae. New Phytol 1983 [cited 2020 Apr 27]; 95(95):697–706.

60. Donath TW, Eckstein RL. Effects of bryophytes and grass litter on seedling emergence vary by vertical seed position and seed size. Plant Ecol 2010 [cited 2020 Apr 27]; 207:257–68.

61. Milberg P, Andersson L, Thompson K. Large-seeded spices are less dependent on light for germination than small-seeded ones. SSR 2000 [cited 2018 Apr 19]; 10(01):99. Available from: URL: https://www.cambridge.org/core/services/aop-cambridge-core/content/view/D6EC82DEDAAFE10F20C1D3A9A2328879/S0960258500000118a.pdf/largeseeded_spices_are_less_dependent_on_light_for_germination_than_smallseeded_ones.pdf.

62. Baskin CC, Baskin JM. Seeds: Ecology, Biogeography, and Evolution of Dormacy and Germination. Lexington: Academic Press; 2014.

63. Facelli JM, Montero CM, Leon RJC. Effect of different disturbance regimen on seminatural grasslands from the subhumid pampa. Flora - Morphology, Distribution, Functional Ecology of Plants 1988; 180:241–9.

64. Cavieres LA, Peñaloza A. Facilitation and interference at the intraspecific level: Recruitment of Kageneckia angustifolia D. Don (Rosaceae) in the montane sclerophyllous woodland of central Chile. Perspectives in Plant Ecology, Evolution and Systematics 2012 [cited 2018 Apr 26]; 14(1):13–9. Available from: URL: https://ac.els-cdn.com/S1433831911000540/1-s2.0-S1433831911000540-main.pdf?_tid=504eb97b-fcf2-4e74-a32d-3bd51b9a3455&acdnat=1524728836_3de92c92b53c85c032201f4918134413.

65. Olson BE, Wallander RT. Effects of invasive forb litter on seed germination, seedling growth and survival. Basic and Applied Ecology 2002; 3(4):309–17. Available from: URL: http://dx.doi.org/10.1078/1439-1791-00127.

66. Siebenkäs A, Schumacher J, Roscher C. Phenotypic plasticity to light and nutrient availability alters functional trait ranking across eight perennial grassland species. AoB PLANTS 2015; 7.

67. Renne IJ, Tracy BF, Colonna IA. Shifts in grassland invasibility: Effects of soil resources, disturbance, composition, and invader size. Ecology 2006; 87(9):2264–77.

Supporting Information

S1 Table. Raw data for seedling emergence and seedling growth according to different treatments.

S2 Table. Results of a three-way ANOVA on the effects of litter type, litter amount and soil on ln biomass per plant.

Fig S1. Biomass per plant - effects of the two-way interaction of litter type and litter amount. In the box plots middle lines represent median, boxes represent the first and third quartiles, lower and upper bars represent the minimum and the maximum and points represent outliers (i.e. points above 1.5 SD). Grey: 200 g/m² litter, black: 400 g/m² litter. Lower case letters indicate significant interactions between treatments (TukeyHSD, $P \leq 0.05$).

 Click here to access/download;Figure;fig1b.TIF

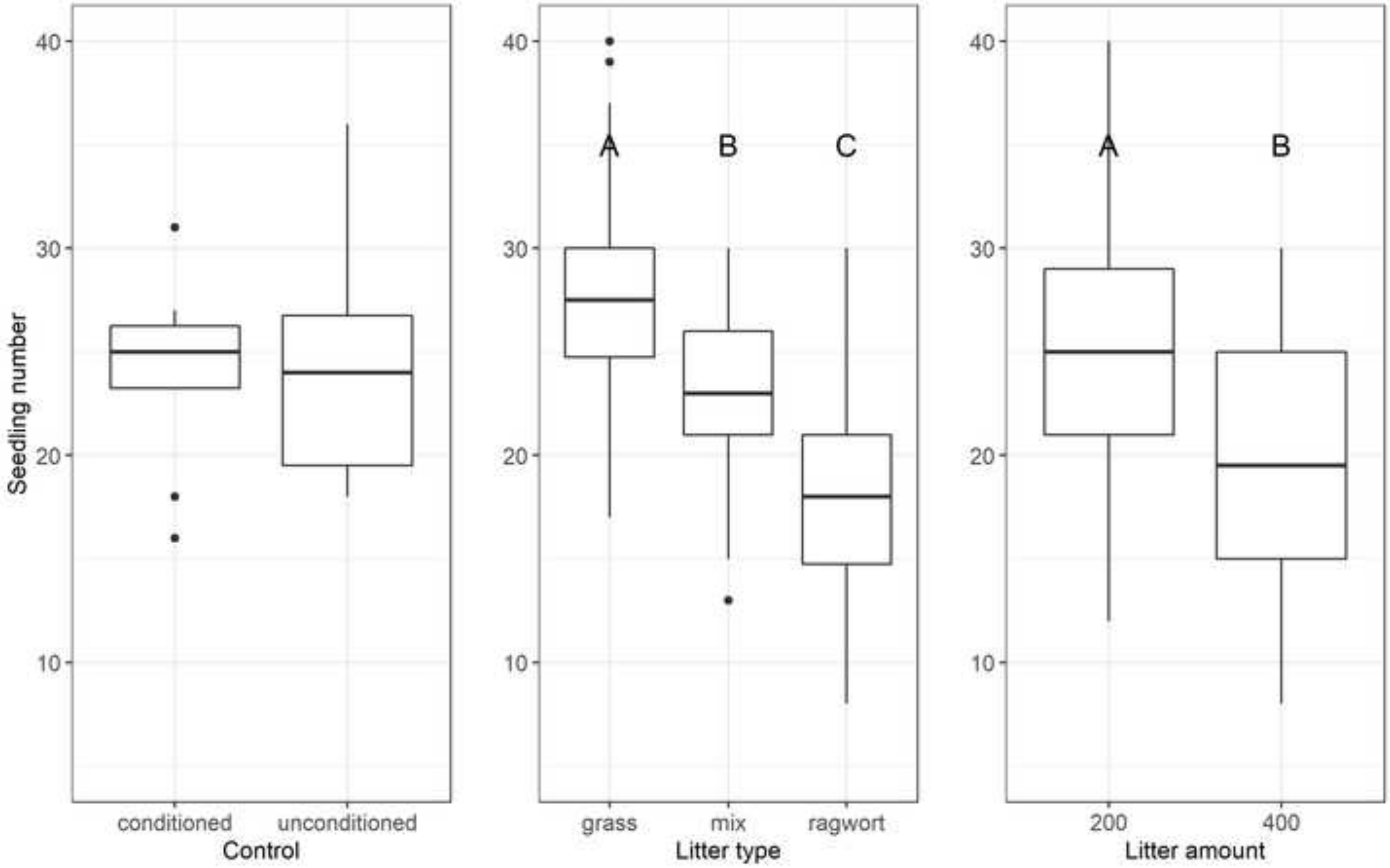

Click here to access/download;Figure;fig2b.tif

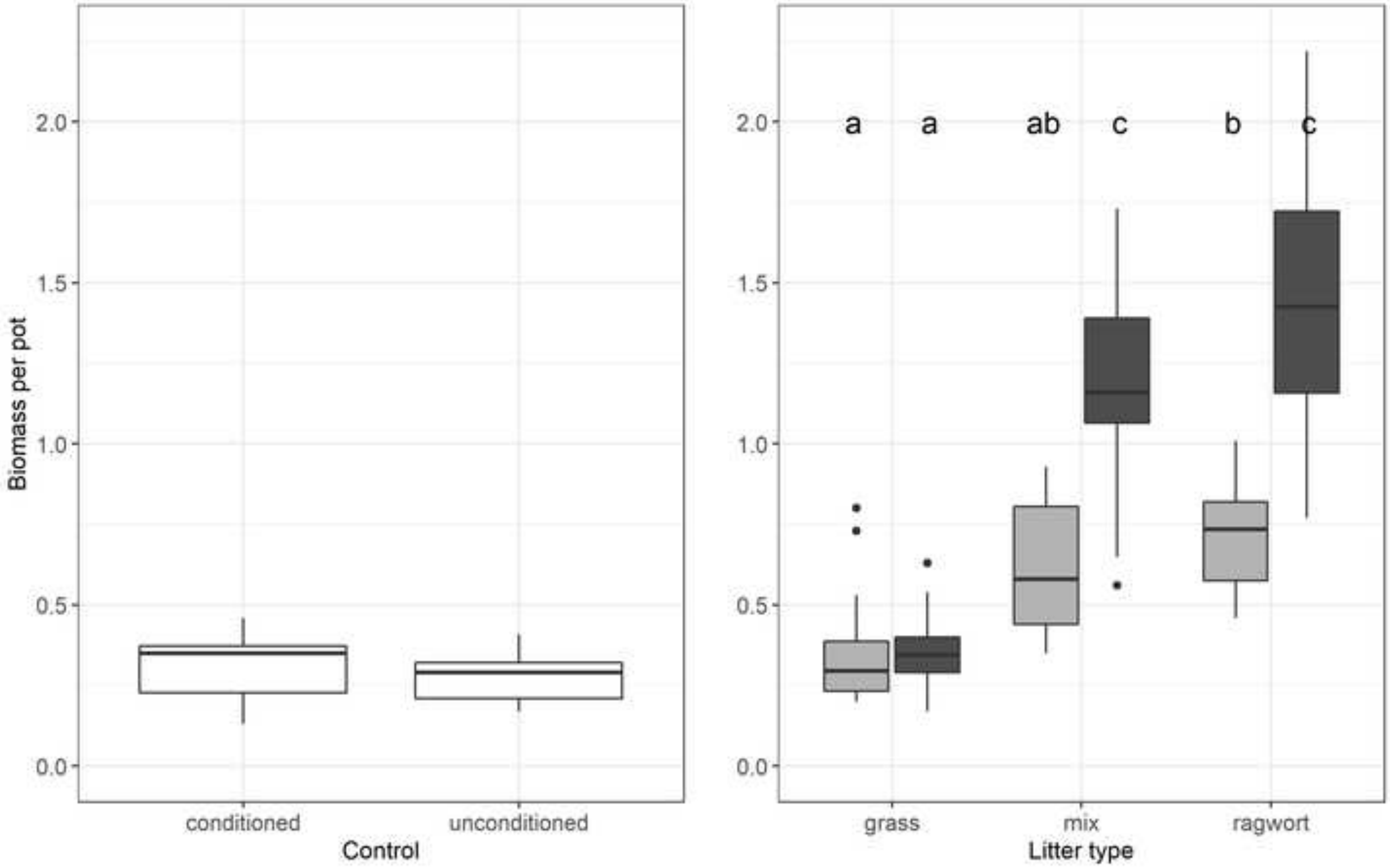

Click here to access/download;Figure;fig3b.tif

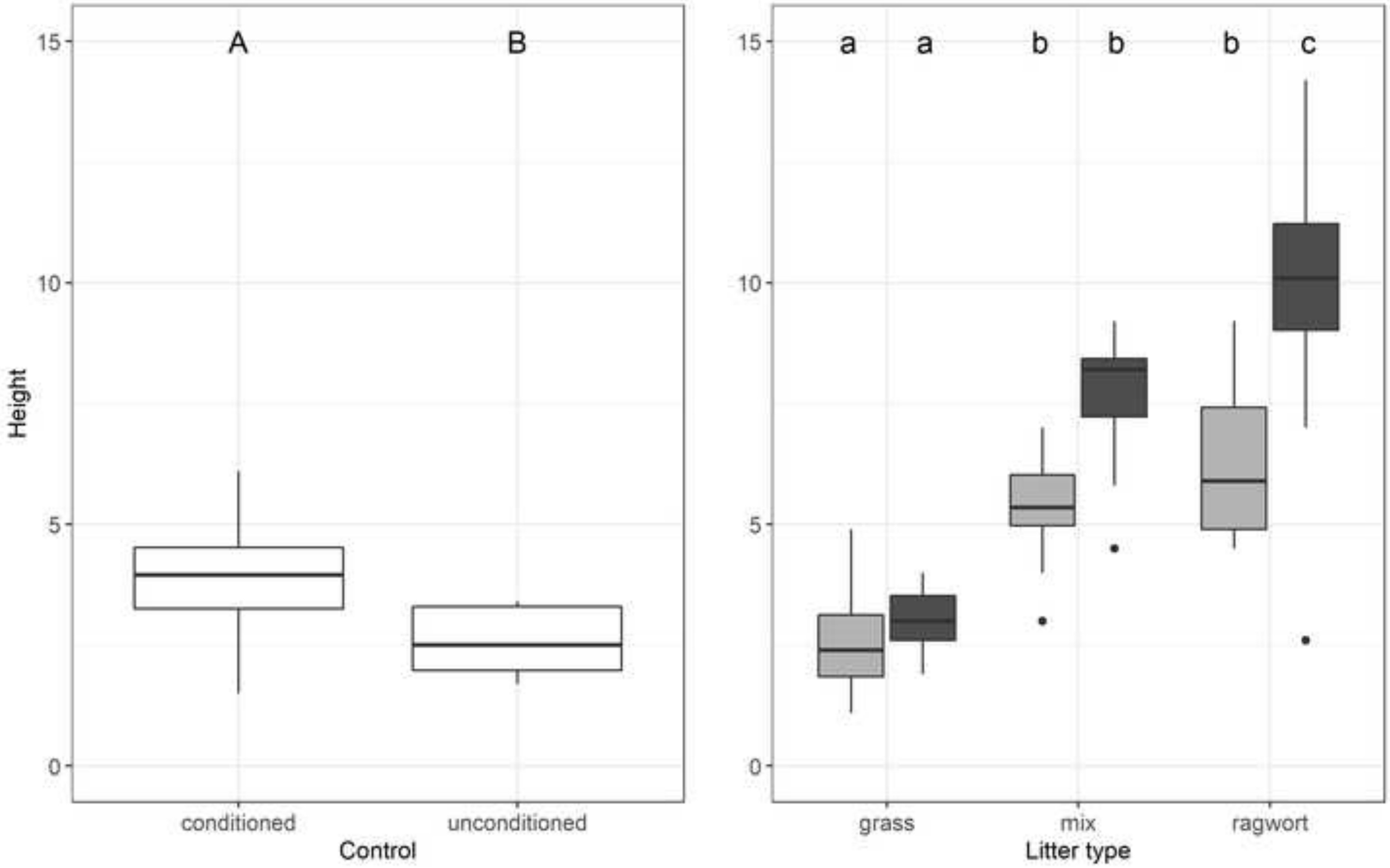

Figure

Click here to access/download;Figure;fig4b.tif

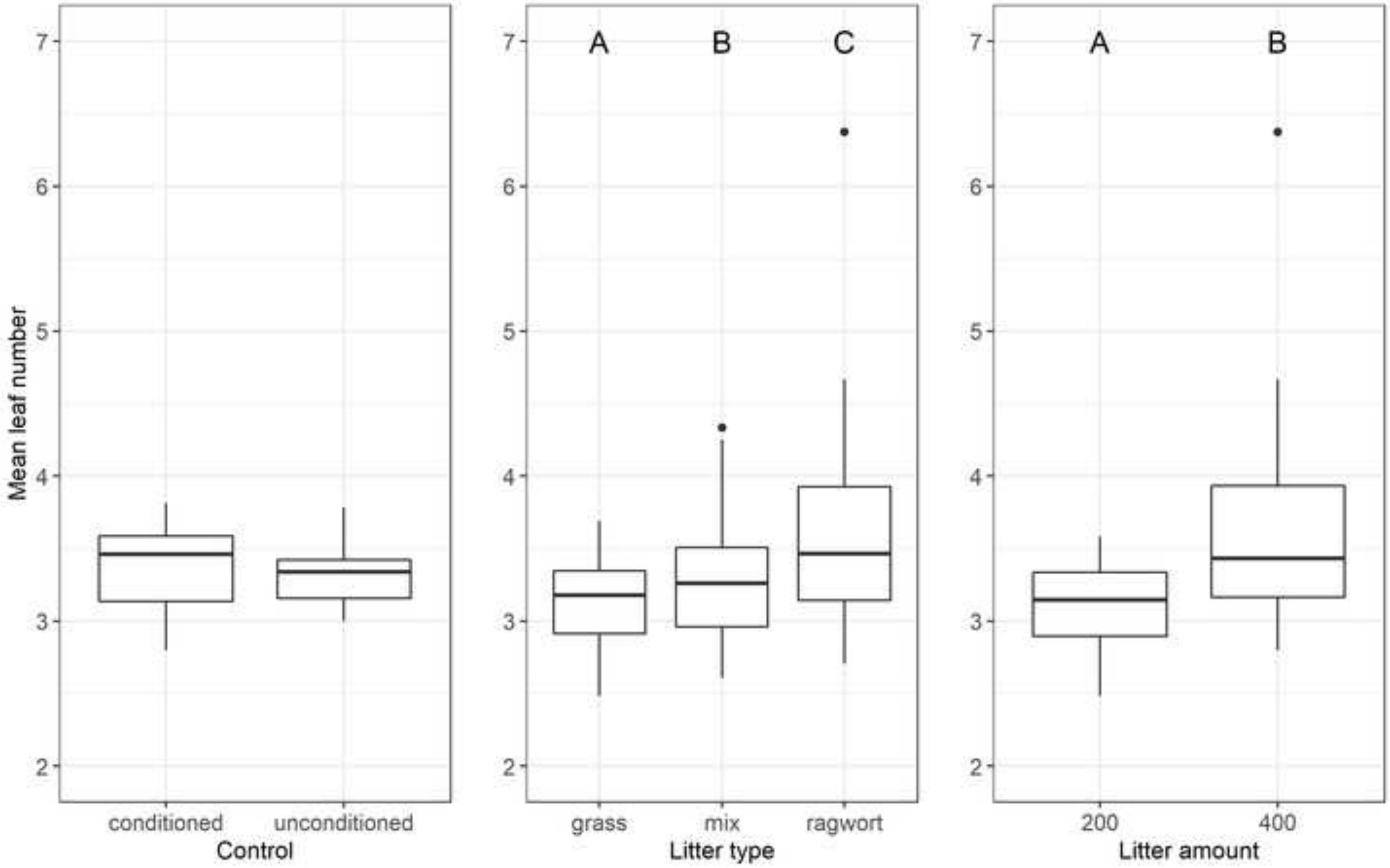

Click here to access/download
Supporting Information
tableS1.csv

Click here to access/download
Supporting Information
suppl_bm_plant.docx

Conspecific and heterospecific litter effects ~~***in ragwort (Jacobaea vulgaris)***~~ *on seedling emergence and growth in ragwort (Jacobaea vulgaris)*

Formatted: Font: 12 pt, Not Bold, Font color: Auto, English (United Kingdom)

Formatted: Affiliation

Formatted: Font: 12 pt, Not Bold, Font color: Auto, English (United Kingdom)

Formatted: Font: 12 pt, Not Bold, Font color: Auto, English (United Kingdom)

Henrike Möhler[1] *, Tim Diekötter[1], Geeltje Bauer[1], Tobias W. Donath[1]

[1]*Department of Landscape Ecology, Institute for Natural Resource Conservation, Kiel University, Germany*

Henrike Möhler hmoehler@ecology.uni-kiel.de +49-431-880-1007

ORCiD 0000-0001-8070-3889

Tim Diekötter tdiekoetter@ecology.uni-kiel.de +49-431-880-1212

ORCiD 0000-0003-4838-793X

Geeltje M. Bauer geeltje.bauer@googlemail.com

Tobias W. Donath tdonath@ecology.uni-kiel.de +049-431-880 1198

ORCiD 0000-0003-1874-1096

*Corresponding author

hmoehler@ecology.uni-kiel.de ~~(HM)~~

Field Code Changed

Conspecific and heterospecific litter effects on seedling emergence and ~~establishment~~growth in ragwort (*Jacobaea vulgaris* ~~Gaertn.)~~)

Abstract

Jacobaea vulgaris Gaertn. or common ragwort is a widespread ~~poisonous~~noxious grassland weed that is subject to different regulation measures worldwide. Seedling emergence and ~~establishment~~growth are the most crucial stages for most plants during their life cycle. Therefore, heterospecific or conspecific litter as well as soil-mediated effects may be of relevance for ragwort control. Our study examines the effects of conspecific and heterospecific litter as well as ragwort conditioned soil on seedling emergence and ~~establishment.~~growth.

We conducted pot experiments to estimate the influence of soil conditioning (with, without ragwort), litter type (grass, ragwort, grass-ragwort-mix) and amount (~~2~~200 g/~~dm², 4~~m^2, 400 g/~~dm~~m^2) on *J. vulgaris* recruitment. As response parameters, we assessed seedling number, biomass, height and number of seedling leaves.

We found that~~, 2~~ 200 g/~~dm~~m^2 grass litter led to higher seedling numbers, while litter composed of *J. vulgaris* reduced seedling emergence. Litter amounts of ~~4~~400 g/~~dm~~m^2 had negative effects on the number of seedlings~~,~~ regardless of the litter type.~~ Significant effects of the soil conditioned by ragwort on seedling emergence were negligible.~~ Results for biomass, plant height and leaf number showed opposing patterns to seedling numbers. Seedlings in pots treated with high litter amounts and seedlings in ragwort litter became heavier, grew higher and had more leaves. Significant effects of the soil conditioned by ragwort on seedling emergence and growth were negligible.

The study confirms that the amount and composition of litter ~~are~~ strongly ~~affecting~~affect seedling emergence and ~~establishment~~growth of *J. vulgaris*. Moreover, while conspecific litter and high litter amounts negatively affected early seedling development in ragwort, those

seedlings that survived accumulated more biomass and got taller than seedlings grown in heterospecific or less dense litter. Therefore, ragwort litter has negative effects in ragwort germination, but positive effects in ragwort growth.

Keywords: ~~*Jacobaea vulgaris*~~*Senecio jacobaea*, mulching, litter, autotoxicity, ragwort, phytotoxicity

Formatted: Font: Italic

Introduction

Jacobaea vulgaris Gaertn. or common ragwort is a widespread ~~poisonous~~noxious grassland weed that is native ~~in~~to Europe, Asia, and Siberia and invasive in North America, New Zealand, and Australia. ~~As~~Since the pyrrolizidine alkaloids of the plant pose a health risk to cattle that consume this plant (1), ~~it is challenging farming when it occurs~~ its occurrence in high densities~~.~~ ~~Thus,~~ is a challenge for grassland management. Consequently, control of common ragwort is prescribed in ~~some countries~~ the UK: ‘‘Weeds Act 1959’’ and ~~is as such included in their legislation (2)~~‘‘Ragwort Control Act 2003’’, Ireland: ‘‘Noxious Weed Act 1936’’, New Zealand: ‘‘Noxious Plant Act 1978’’, Australia: ‘‘Weed Management Act 1999’’, and Friesland, province of the Netherlands ‘‘Verordening Jakobskruiskruid 2007’’(2). ~~Consequently~~Therefore, several studies have aimed ~~at trying~~ to find effective and efficient ways ~~how~~ to manage this grassland weed ~~(2–5).~~(2–5). Some of these studies ~~come~~came to the conclusion that specific cutting regimes are a promising approach (6). However, the toxicity of ragwort biomass precludes ~~the~~its use as hay or silage and ~~a~~the high number of long~~-~~-living seeds rules out composting ~~the~~ragwort biomass~~.~~ (7, 8). Consequently, the biomass is often disposed of as special waste, which is costly ~~(7).~~(9). Yet, the occurrence of conspecific allelopathic effects ~~(8, 9)~~(10, 11) suggests that leaving ragwort litter on ~~the~~ mown grassland could be an additional component in managing ~~the~~ragwort abundance ~~of ragwort~~. As a first step to evaluate the validity of this approach, we experimentally tested this management option.

~~Litter influences micro-environmental conditions, is important for nutrient cycling and can have prominent effects on seedling establishment and germination via mechanical and chemical effects (10, 11). Litter is affecting the physical environment by lowering light availability, changing the water and temperature regime and can act as physical barrier (11). Litter may also exert chemical effects by changing the availability of nutrients or releasing phytotoxins (9, 12,~~

13). Through these mechanisms litter potentially influences community organization via delaying and reducing or facilitating seedling emergence or seedling establishment (11, 14).

Albeit a meta-study on litter effects showed an overall negative effect of litter on seedlings (15), another meta-analysis demonstrated that litter can be also beneficial for seedling establishment especially under dry conditions (e.g. dry grasslands or dry periods) or at low to medium litter amounts (11). Positive effects were due to higher soil humidity and a lower amplitude of diurnal temperature fluctuations beneath a low litter cover (12). In addition to the amount of litter, if seedling establishment is facilitated or hampered, also crucially depends on litter type (16). A thick litter layer may cause limited access to light for seedlings and poses a physical barrier for their growth. Grass litter, for example, might be less hampering than large-leafed forb litter due to a lower amount of phytotoxins and an easier to penetrate structure (13, 16). Under natural conditions, though, litter mostly is not monospecific, e.g. in grasslands it consists of a mixture of forb and grass litter. The effects of litter from plant communities are driven by the species-specific litter properties (17) and may either be purely additive as hypothesized in the mass-ratio hypothesis (18) or non-additive as litter types may interact. In this context Gartner and Cardon (19) found that more than half of the litter mixtures they reviewed behaved in a non-additive way concerning factors like mass loss, nutrient transfer or decomposer composition.

It is intuitive to hypothesize that conspecific litter is facilitating the establishment of conspecific seeds. Yet, as intraspecific competition is assumed to be more intense, than interspecific competition (20) also inhibiting effects may be expected. While many studies have shown facilitating effects from adults on conspecific seedlings (21–23), particularly in weed species (24–26), there are also studies demonstrating the opposite (27, 28). Some did not find evidence of unidirectional conspecific facilitation (29). In line with the latter, studies on ragwort showed autotoxic potential of ragwort litter and soil from ragwort stands (30; 9). Also, a negative plant-soil feedback has been demonstrated for ragwort, which is probably driven by differences in

the soil community (31, 32). Under these circumstances, negative effects of ragwort litter and ragwort conditioned soil may either be additive, i.e. result in stronger inhibitory effects than they would do individually, or the combined effect is not additive, i.e. combined effects are lower or higher than the sum of both effect sizes. However, the influence of litter during seedling emergence might be weaker than during seedling establishment, since at the latter stage impacts of competition are of higher importance than direct litter effects (33). Thus, if litter reduces seedling density through self-thinning surviving seedlings might be in advantage later on, as intraspecific competition is reduced.

Litter influences micro-environmental conditions, is important for nutrient cycling and can have prominent effects on seedling germination and growth via mechanical and chemical effects (12, 13). Litter has been shown to have both positive and negative effects on seedling germination and establishment (13, 14). Positive effects were due to higher soil humidity and a lower amplitude of diurnal temperature fluctuations beneath a low litter cover (15). Still the size of litter effects on seedling germination and establishment do also depend on the amount of litter present. If litter layer is too high, it reduces the amount of light that reaches the seeds, which reaching seeds, where seeds of ragwort depend on a certain light amount to germinate (16). In addition, thick litter layers that preserve a relative high humidity can facilitate the spread of mould that may cause decay of seeds (17). Van der Mejden and van der Waals-Koi showed that ragwort seedlings need light for germination (16). If not enough light is reaching the seeds, dormancy will be induced. On the other side they also demonstrated that germination was facilitated under light sand cover compared to no cover. They found that seeds need a certain moisture to germinate. Short periods of frost or drought can induce dormancy even if favourable conditions are restored again. After germination seedlings can be hampered in their growth or even die if litter amounts are too high. Thus Van der Meijden and van der Waals-Koi found significantly less seedlings in pots with high vegetation cover (16). Litter may also exert

chemical effects by nutrient immobilization, depletion of oxygen in the soil or toxicity of carbon dioxide produced by decomposers or by releasing phytotoxins (11, 15, 18). Through these mechanisms litter potentially influences community organization via delaying and reducing or facilitating seedling emergence or seedling growth (13, 19).

Litter type is another important factor that can influence whether seed germination and seedling establishment is facilitated or hampered (20). It changes conditions for germination and growth as chemical as well as physical structure depend on the characteristics of species (20). Grass litter, for example, might be less hampering than large-leafed forb litter due to a lower amount of phytotoxins and an easier to penetrate structure (18, 20). Under natural conditions, however, litter is not usually monospecific, e.g. in grasslands it consists of a mixture of forb and grass litter. The effects of litter from plant communities are driven by species-specific litter properties (21) and may either be purely additive as hypothesized in the mass-ratio-hypothesis (22) or non-additive, as litter types may interact. In this context, Gartner and Cardon (23) found that more than half of the litter mixtures they reviewed behaved in a non-additive way concerning factors such as mass loss, nutrient transfer or decomposer composition.

It is intuitive to hypothesize that conspecific litter facilitates the establishment of conspecific seeds. Yet, as intraspecific competition was found to be more intense than interspecific competition (24, 25), inhibiting effects may also be expected. While many studies have shown facilitating effects of adult plants on conspecific seedlings (26–28), particularly in weed species (29–31), there are also studies demonstrating the opposite (32, 33). Some authors did not find evidence of unidirectional conspecific facilitation (34, 35). In line with the latter, studies on ragwort showed the negative autotoxic potential of ragwort litter and soil close from ragwort plants (36; 11). Also, Bezemer et al. 2006 and Van de Voorde et al. 2011 found a negative intraspecific plant-soil-feedback for ragwort, which they assumed is probably driven by pathogens that accumulate in the soil community (37, 38). Under these circumstances, negative

effects of ragwort litter and ragwort conditioned soil on ragwort performance (e.g. lower biomass) may either be additive, i.e. result in stronger inhibitory effects than they would do individually, or the combined effect is not additive, i.e. combined effects are lower or higher than the sum of both effect sizes. However, the influence of litter during seedling emergence might be stronger than during seedling growth, since at the latter stage impacts of competition are of higher importance than direct litter effects (39). Thus, if litter reduces seedling density through self-thinning, surviving seedlings might have an advantage later on, as intraspecific competition would be reduced (40).

The present study is to our knowledge the first to test the influence of the combined effects of litter type, amount and ~~conditioned~~ soil conditioning on seedling emergence and ~~establishment~~growth in ragwort. To this end, we set up a common garden experiment, where we assessed the conspecific and heterospecific effects of litter on the number of emerged ragwort seedlings, their biomass, height, leaf number and specific leaf area. We posed the following questions:

I. Does litter type influence the emergence and ~~establishment~~growth of ragwort seedlings?

Assuming conspecific negative effects, we expect lower numbers of ragwort seedlings to emerge from beneath conspecific litter compared to pure grass litter or a mixture of grass and ragwort litter. We expect ~~congruent~~similar patterns to occur in seedling ~~establishment~~growth after emergence.

II. Does litter amount influence seedling emergence and ~~establishment~~growth of ragwort and does this depend on litter type?

We expect negative litter effects to increase with a higher litter cover and to be stronger with increasing litter amount. We also expect the size of litter effects to increase with the portion of ragwort litter in the litter cover. Thus, we expect ~~an~~the interaction of litter type and amount ~~in that seedling's~~on seedling emergence and ~~establishment is~~

~~supposed~~growth to be lowest in high amounts of ragwort litter~~.~~. Seedling performance is hypothesized to be higher ~~in~~under medium ragwort amounts and ~~even higher in~~highest under low amounts of grass litter.

III. Does ragwort-conditioned soil influence conspecific seedling emergence and ~~establishment?~~growth? If so, do these effects interact with the litter effects?

We hypothesized an inhibitory effect of ragwort-conditioned soil on its own emergence and ~~establishment~~growth as negative plant-soil feedback for this species has been observed. We expect these effects to be additive for ragwort litter.

Material and methods

Study species

Jacobaea vulgaris L. syn. *Senecio jacobaea* Gaertn. or common ragwort is a herbaceous monocarpic plant from the Asteraceae family ~~(34).~~(41). It is a hemicryptophyte, forming a rosette in the first year and flowering in the second year (~~34).~~41). It prefers open patches and disturbed sites. Thus, it occurs on ruderal sites, along roadsides, pastures and grassland ecosystems in most temperate regions of the world. It is often regarded as a noxious weed due to its content of toxic pyrrolizidine alkaloids ~~(2).~~(2). Ragwort shows ecological characteristics of a pioneer species, in particular its numerous light seeds and fast growth enable ragwort to spread rapidly in open areas (1). Each plant produces several thousand seeds per year (1). ~~Seeds are mainly dispersed by wind over a distance of usually less than 15 m (35).~~Seeds are mainly dispersed by wind over a distance of usually less than 15 m (42). Seed survival (up to 20 years) and germination rates (about 80%) are high (~~36).~~43).

Experimental design

We studied the effects of *litter amount* (*k* = 2; 2 (low) vs. 4 (high) g/dm^2), *litter type* (*k* = 3; ragwort litter, grass litter, 1:1 mix of ragwort and grass litter) and *conditioned soil* (*k* = 2; soil conditioned with ragwort vs. unconditioned soil that is soil **not** conditioned by ragwort) on seedling emergence and growth. The litter amounts applied correspond to the range of average productivity of extensively managed grasslands in Northern Germany (44). Loydi et al. ~~4 (high) g/dm^2), *litter type* (*k* = 3; ragwort litter, grass litter, mix of ragwort and grass litter) and *soil conditioned* (*k* = 2; soil conditioned with ragwort vs. unconditioned soil) on seedling emergence and establishment. High litter densities refer to average regional productivity of grasslands in Northern Germany (37) and thus represent average litter quantities that ragwort seeds are exposed to at heavily ragwort-infested sites.~~(11) found positive litter effects to occur at these litter amounts. Thus, the litter amounts applied represent litter quantities that ragwort seeds are exposed to naturally and that seem to affect germination.

~~Seeds of *J. vulgaris* were hand-collected from at least 20 plant individuals on each of eight extensively managed pastures across Schleswig-Holstein from August to September 2015 and mixed for the experiment. These seeds were dry stored in darkness at room temperature until sowing on 21st April 2016. An initial viability test with a 1% tetrazolium chloride solution (38) on additional seed batches showed that most seeds (> 80%) were viable. Litter of *J. vulgaris* and conditioned and unconditioned soil was collected from rosettes on a ragwort-infested extensive cattle pasture near Preetz, Schleswig-Holstein in April 2016. We assumed soil affected by ragwort, i.e. conditioned, if at least four ragwort-plants were growing within 0.5 m^2. Unconditioned soil was taken from the center of an area (diameter 10 m) where no ragwort was growing. Litter was collected, cleaned and dried for two days at room temperature and for 24 hours at 40 °C. Litter levels of 2 g/dm^2 and 4 g/dm^2 were chosen, since~~ Seeds of *J. vulgaris* were hand-collected from at least 20 plant individuals on each of eight extensively managed pastures across Schleswig-Holstein from August to September 2015 and mixed for the

experiment. These seeds were dry stored in darkness at room temperature until sowing on 21st April 2016. An initial viability test with a 1% tetrazolium chloride solution (45) on additional seed batches showed that most seeds (> 80%) were viable. *J. vulgaris* litter and conditioned and unconditioned soil was collected from rosettes on a ragwort-infested extensive cattle pasture near Preetz, Germany (54.227175, 10.242809) in April 2016. We assumed soil to be affected by ragwort, i.e. conditioned, if at least four ragwort-plants were growing within 0.5 m². We refer to unconditioned soil when soil was taken from the center of an area (diameter 10 m) where no ragwort was growing. Litter was collected, cleaned and dried for two days at room temperature and for 24 hours at 40 °C to constant weight. This way we ensured that chemicals potentially affecting germination were not changed. For grass litter, we used dried plant material from a grassland mainly consisting of sweet grass (Poaceae) species.

We set up a completely~~Loydi et al. (11) found positive litter effects to occur at these litter amounts. As grass litter, we used dried plant material from a grassland mainly consisting of sweet grass (Poaceae) species.~~

~~We used a~~ randomized experimental design. We prepared pots of 1 dm^3 volume (c. 10 × 10 × 10 cm) for each litter amount × litter type × soil conditioned (12 combinations) replicated eight times. To ~~account~~assess for seedlings emerging from the soil taken from ragwort stands we additionally included 20 control pots, ten with ragwort conditioned soil and ten filled with unconditioned soil. In none of these pots ragwort seedlings emerged. Half of the pots ~~was~~were filled with a 6:1 mixture of commercial potting soil (Fruhstorfer Erde®, Type P, Industrie-Erdenwerke Archut GmbH, Germany) and ragwort-conditioned soil or unconditioned soil, respectively. We followed the protocol of van de Voorde et al. ~~(39)~~(46) concerning inoculation and mixing of soil, as it led to significant differences between conditioned and unconditioned soil in their experiment.

In late April 2016, we sowed 50 seeds in each experimental pot~~.~~ and added litter on top of it. Pots were placed randomly in a common garden in Kiel, Germany (~~N54° 20.81167 14, E10° 6.345185~~54.346883, 10.106829), surrounded by another row of pots (to reduce desiccation) and protected by a light metal grid to avoid litter being blown away. Pots were kept constantly moist by regular watering.

Germination started ~~mid of May 2016. Seedlings that emerged above the litter cover were counted weekly until the tenth week after the experiment started. On 30 June, we counted total number of leaves per plant and measured plant height of the highest plant in the pot. We determined specific leaf area for five pots per combination by cutting the youngest fully developed leaves of three plants. These were scanned and leaf area in mm² measured by the programme IMAGEJ (40).~~in mid May 2016. Seedlings that emerged above the litter cover were counted weekly until the tenth week after the experiment started. After the tenth week, we counted the total number of leaves per plant and measured the height of the highest plant in the pot. We determined specific leaf area for five pots per combination by cutting the youngest fully developed leaves of three plants. These were scanned and leaf area in mm² measured by the programme IMAGEJ (47). Afterwards leaves were dried at 60 °C and weighed to the closest milligram. The same was done with total aboveground biomass.

Data analysis

~~We tested differences in number of seedlings emerged and measures of seedling establishment represented by: total biomass per plant, plant height, leaf number and specific leaf area by calculating natural logarithmic response ratios (LnRR) to estimate the responses to the litter types and amounts and the soil type. LnRR were standardized with the mean of the control treatment of our two control groups (unconditioned soil or no-litter treatments). Therefore, they were well suited to test for differences between the other factors. LnRR was calculated as proposed by Koorem et al. (41):~~

~~LnRR = Ln (measured parameter value of the treated sample/mean value of the corresponding control)~~

~~The effect of litter or soil was considered significant when the confidence interval did not overlap with zero (i.e. control and treatments are significantly different). To compare the LnRR among treatments, we used a factorial ANOVA to analyze the effect of litter type, litter amount and soil type, and their interactions. Graphical residual analysis revealed that the data met assumptions of analysis of variance, i.e. normal distribution and variance homogeneity. We used the Tukey-HSD-Test as Post-hoc-Test. As a measure for the relative contribution of each factor and their interactions to the total variability of the response parameters of interest, we used the ratio of the sum of squares of the factor or interaction of interest to the total sum of squares (i.e. for all factors, their interactions and the error). In order to ensure readability, we refrain from prefixing the response variables with LnRR in the following text. All analyses were done using R version 3.6.1 (42).~~

We tested for differences in number of emerged seedlings and measurements of seedling growth represented by: total biomass per pot and per plant, plant height, leaf number and specific leaf area. We used a three-factorial ANOVA to analyze the effect of litter type, litter amount and soil type, and their interactions. Residuals were examined visually to assess validity of assumptions for analysis of variance, i.e. normal distribution and variance homogeneity. Data for biomass per pot and biomass per plant had to be ln transformed to meet assumptions of analysis of variance. Subsequent to significant ANOVA results, post-hoc Tukey-HSD test was employed to test for individual differences. In addition, we used contrasts to specifically test whether the effect of mixed litter on response variables was mid-way between that of grass and ragwort alone (additive effect of pure type litters in the mixture). Since not general litter effects but specific effects of the different litter types on seedling emergence and growth of ragwort

were the focus in our study, we did not include the controls in our model but included them for visual comparison in the figures. In addition, we analyzed the controls for soil effects as well as for differences to the litter types across litter amount using contrasts. As a measure for the relative contribution of each factor and their interactions to the total variability of the response parameters of interest, we used the ratio of the sum of squares of the factor or interaction of interest to the total sum of squares (i.e. for all factors, their interactions and error). All analyses were done using R version 3.6.1 (48).

Results

In general, our results showed significant main effects of litter type and litter amount on all response variables~~, i.e.~~: seedling number, biomass per plant and pot, plant height ~~as well as on~~ and mean leaf number. In contrast, the soil factor ~~soil, i.e.~~ (conditioned vs. unconditioned~~,~~ ~~influenced only~~) was not significant for any response variables under the litter treatments. Only the case of the separate analysis of controls soil revealed a significant effect on plant height ~~significantly.~~. Our analyses revealed ~~only one~~two significant ~~interaction~~interactions between main factors~~, i.e. in~~. In the case of biomass per plant and pot and seedling height we found a significant two-way *litter type* × *litter amount* interaction. Effects of the treatments on specific leaf area were not significant (results not shown).

Seedling emergence

~~While~~We found that while both, litter type and litter amount~~ both~~, affected seedling emergence significantly, the amount of explained variance was more than twice as high for litter type than litter amount (~~37.88~~38.22 % vs. ~~17.01~~16.58 %; Table 1). ~~Seedling number~~Number of emerged seedlings differed significantly between all three litter types (Fig. 1). ~~but only grass and ragwort litter effects~~ In addition, contrast analyses of the control against litter types revealed that the litter mixture had no significant effect on seedling number compared to the control (p = 0.30),

Formatted: Font: +Body (Calibri), 11 pt, Not Italic, Font color: Auto

while in the control seedling number differed significantly from the control (95% CI does not include zero; Fig 1). In grass and ragwort litter (p = 0.016 and p ≤ 0.0001, respectively). From beneath grass litter, seedling emergence was one third higher (28 ± 1; mean ± SE) than in significantly raised by about 50% compared to ragwort (18 ±litter (18.1 ± 1.0 vs. 27.6 ± 0.8), while emergence from beneath mixed litter was intermediate (22.8 ± 0.8) (Fig. 1). Litter effects were additive, i.e. the effect size of mixed grass- 1). Interestingly, ragwort litter had a negative effect on seedling number across both did not deviate significantly from the expected mean effect of pure grass and ragwort litter (p = 0.99). Across litter types higher litter amounts, while the opposite was true in case of pure grass litter. Across different litter types an increased litter cover, i.e. 4 of 400 g/dm^2, led to am^2, significantly reduced seedling number, both in comparison to the control and the numbers by about one fifth, compared to low litter cover of 2200 g/$dm^2$$m^2$ (24.2 ± 1.0 vs. 20± 1) (Fig 1).

Table 1. Results of a three-way ANOVA on the effects of litter type, litter amount and soil on LnRR of seedling number and measures of seedling establishmentgrowth (biomass per plant, plant height and mean leaf number).

		LnRR Seedling number				LnRRLn Biomass per plantpot				LnRR Height				LnRR meanMean leaf number			
**Source of variation**	df	MSQ	F	*P*	vc	MSQ	F	*P*	vc	MSQ	F	*P*	vc	MSQ	F	*P*	vc
**Litter type**	2	**0.6003726.8**	**37.57838.107**	**≤ 0.0001**	**37.8838.22**	**19.75610.824**	**190.19795.940**	**≤ 0.0001**	**62.4255.23**	**9.844234.63**	113.71398.618	**≤ 0.0001**	**57.8856.51**	**0.11731.602**	**8.2067.868**	**≤ 0.0001**	**11.9464**
**Litter amount**	1	**0.5393630.4**	**33.755053**	**≤ 0.0001**	**17.0116.58**	**12.355**	**118.94755.415**	**≤ 0.0001**	**19.5213.82**	**2.459105.84**	28.40044.486	**≤ 0.0001**	**7.2312.74**	**0.43305.505**	**30.29627.037**	**≤ 0.0001**	**22.0420.02**
**Soil**	1	0.025442.7	1.5892.237	0.211138	0.801.12	0.008	0.075072	0.785427	0.0118	**3.9470**.07	**45.599**0.030	**≤** 0.**000**1864	**11.60**0.01	0.0005108	0.037532	0.847468	0.0340
**Litter type x litter amount**	2	0.021152.3	1.321371	0.272259	1.3338	**1.252**	**12.0551.127**	**≤ 0.0001**	**3.965.75**	0.1472**1.84**	1.703**9.181**	**≤ 0.**188**0001**	0.865**.26**	0.0305542	2.137663	0.124076	3.1094
**Litter type x soil**	2	0.008621.3	0.536558	0.587575	0.5456	0.043	0.410125	0.665333	0.1464	0.01514	0.172059	0.842943	0.0904	0.0057104	0.398512	0.673601	0.5875
**Litter amount x soil**	1	1.0.0004	0.027055	0.871816	0.0103	0.004	0.042001	0.839924	0.0100	0.05533.15	0.63711.326	0.427253	0.1640	0.0014012	0.098059	0.755809	0.0704
**Litter type x litter amount x soil**	2	0.00031	0.0181	0.982004	0.0200	0.049	0.475038	0.623336	0.1519	0.11633.99	1.341679	0.267192	0.6896	0.0109139	0.761684	0.471507	1.1101
**Error**	84	1602.0.0160			42.4012	0.104			13.8024.18	0.08722.38			21.4924.07	0.0143204			61.1362.18

d.f. = degrees of freedom; MS = mean sum of squares; vc (%) = relative contribution of individual factors and their interactions to total variation.

Significant effects (*P* < 0.05) are given in bold.

Fig 1. ~~LnRR of seedling~~ Seedling number: effects of soil conditioning on the control, litter type~~, i.e.~~ (grass, mix, ragwort (left~~)~~)) and different litter amounts~~, i.e. 2~~ (200 g/~~dm~~m² litter and ~~4~~400 g/~~dm~~m² (right~~). Shown are the means ± 95% CI. Letters~~)). In the box plots middle lines represent median, boxes represent the first and third quartiles, lower and upper bars represent the minimum and the maximum and points represent outliers (i.e. points above 1.5 SD). Upper case letters indicate significant ~~differences~~main effects between treatments (TukeyHSD, P ≤ 0.05). ~~Effects of treatments were considered significant (i.e. different from the controls) when 95% CI did not overlap with zero.~~

Seedling ~~establishment~~growth

~~Effects of litter type and amount on biomass per plant were also significant but diametric to the results for seedling emergence (Fig 2).~~ More than half of the variance ~~within~~in biomass per ~~plant~~pot was explained by litter type, whereas litter amount only explained around one ~~fifth~~tenth of the variance (Table 1). The significant interaction ~~between litter type and amount was also significant, but rather weak as it only~~ , which accounted only for ~~around 4~~6% of the ~~explained~~ variance~~. The interaction~~, was ~~quantitative in nature (Fig 2) as only the size but not the sign of the~~caused by increased biomass with increasing litter amount when litter contained ragwort compared to no effect ~~changed with~~of amount in grass litter ~~type or litter amount, respectively. This means that the pattern of low~~(Table 1; Fig. 2). Across litter amounts ~~leading to higher seedling mass occurred in all litter types but was especially pronounced~~biomass per pot was more than doubled for seedlings that grew in ragwort litter compared to ~~mixed litter and grass litter albeit seedling biomass was not significantly different from the control~~ those in ~~the~~ grass litter ~~treatment.~~

While (1.1 ± 0.1 g vs. 0.4 ± 0.1 g) (Fig. 2). Interestingly, seedling biomass per plant differed significantly between all pot in the litter mixture (0.9 ± 0.1 g) lay between that of seedlings growing in pure grass or ragwort litter but in contrast to seedling emergence, the litter type effects were not additive, i.e. seedling biomass per pot in the mixed litter treatment lay significantly above the mean of seedling biomass in grass and ragwort litter ($p = 0.001$, Fig. 2). Across litter types, biomass per plant and per pot increased with increasing litter amount applied (0.56 ± 0.03 g in low litter vs. 1.00 ± 0.08 g in high litter). Contrast analyses of the control against the three litter types, only mixed and ragwort litter treatment led to significantly more revealed no significant difference of biomass per plant than inpot between the control (95% CI does not include zero;and grass litter ($p = 0.40$) but significant differences in biomass between control and litter mixture as well as ragwort litter (both $p \leq 0.0001$). Seedling biomass per plant showed almost the same response patterns but the litter effect was additive ($p = 0.88$; Supporting information Fig S1).

Fig 2). Highest biomass amounts occurred in the treatment with high ragwort litter amounts.

Formatted: Font: Bold

Fig 2. LnRR. Biomass per pot - effects of biomass per plant – effects ofsoil conditioning on the control and the two-way interaction of litter type and litter amount. Shown are the means ± 95% CI.In the box plots middle lines represent median, boxes represent the first and third quartiles, lower and upper bars represent the minimum and the maximum and points represent outliers (i.e. points above 1.5 SD). Grey: 2200 g/dmm² litter, black: 4400 g/dmm² litter. LettersLower case letters indicate significant differencesinteractions between treatments

(TukeyHSD, P≤ 0.05). ~~Effects of treatments were considered significant (i.e. different from the controls) when 95% CI did not overlap with zero.~~

Seedling height was largest for seedlings growing in high amounts of ragwort litter (Fig. 3). Across amounts seedlings in ragwort litter grew almost three times as tall as in grass litter (8.1 ± 2.8 cm vs. 2.8 ± 0.9 cm) and about one third taller compared to the litter mixture (6.5 ± 0.3 cm). Plant height was significantly influenced by the main factors litter type and litter amount ~~(Fig 3). The pattern is the same as for biomass as such that all litter types differed and seedlings in mixed and ragwort litter grew highest.~~as well by the interaction of these factors (Fig 3). More than half of the variance was explained by litter type (Table 1). ~~Litter~~Again litter type effects were not additive, i.e. seedling height per pot in the mixed litter treatment lay significantly above the mean of seedling height in grass and ragwort litter (p = 0.001, Fig. 3). Contrast analyses of the control against the three litter types revealed no significant difference of height between the control and grass litter (p = 0.39) but significant differences between control and litter mixture as well as ragwort litter (both p ≤ 0.0001). In contrast, litter amount had a much smaller~~ but also~~, still significant effect~~:~~, i.e. higher litter amounts led to taller seedlings~~.~~ (Fig. 3). Seedling height is the only response variable ~~that ~~where the control was significantly influenced by soil type~~. Ragwort~~, i.e. ragwort conditioned soil led to significantly ~~lower~~taller seedlings (3.9 ± 0.5 cm vs. 2.6 ± 0.3 cm).

Fig 3. Seedling height - effects of ~~seedlings. There was no~~**soil conditioning on the control and the two-way interaction** ~~between the different factors. In all treatments, seedling height was different from the control. Only seedling height in grass litter was significantly lower compared to seedlings growing in the control pots. All other seedlings grew bigger.~~**of litter type and litter amount.** In the box plots middle lines represent median, boxes represent the first and third quartiles, lower and upper bars represent the minimum and the maximum and

Formatted: Font: Bold

Formatted: Font: Bold

Formatted: Font: Bold

points represent outliers (i.e. points above 1.5 SD). Grey: 200 g/m² litter, black: 400 g/m² litter. Lower case letters indicate significant interactions between treatments (TukeyHSD, P≤ 0.05).

Fig 3. LnRR of seedling height - effects of litter type, i.e. grass, mix, ragwort (left), different litter amounts, i.e. 2 g/dm² litter, 4 g/dm² litter (middle) and soil, i.e. ragwort conditioned, unconditioned (right). Shown are the means ± 95% CI. Letters indicate significant differences between treatments (TukeyHSD, P≤ 0.05). Effects of treatments were considered significant (i.e. different from the controls) when 95% CI did not overlap with zero.

Mean leaf number The mean leaf number of plants growing in ragwort litter was highest compared to in plants growing in grass litter and the combination of both litter types (ragwort 3.59 ± 0.12, grass 3.15 ± 0.05, mixture 3.31 ± 0.08). Analyses of variance revealed that mean leaf number was significantly affected by litter type and amount (but not their interaction (Table 1; Fig 4). The variance explained variance forby litter amount was twice as high as inthat explained by litter type. (Table 1). Thus, mean leaf number is the only measured variable that seems to be stronger influenced from litter amount than litter type. Leaf number of plants in grass litter was significantly lower than in the control treatment, whereas leaf number in ragwort was higher, albeit not significantly, and leaf number of seedlings growing in mixed litter was comparable to the control. Higher litter amounts led to significantly higher number of leaves compared to the control treatment, while the opposite was true for the low litter cover. Effects of the treatments on specific leaf area were not significant (results not shownEffects of litter type were additive, i.e. the leaf number in the litter mixture did not differ significantly from the expected mean effect of pure grass and ragwort litter (p = 0.49, Fig. 4). Plants growing in high litter amounts (400 g/m²) had significantly more leaves per plant than plants growing in the low litter amounts (200 g/m²). Contrast analyses revealed no significant difference in leaf number between the plants growing in the control and in any of the three litter types (p > 0.05).

Fig 4. Mean (± 95% CI) LnRR of mean leaf number under soil conditioning in the control, different litter types: (grass, mix, ragwort (left))) and different litter amounts: 2 g/dm^2 litter, 4 g/dm^2 litter (right). Shown are the means ± 95% CI. Letters **(200 g/m^2 litter, 400 g/m^2 litter (right)).** In the box plots middle lines represent median, boxes represent the first and third quartiles, lower and upper bars represent the minimum and the maximum and points represent outliers (i.e. points above 1.5 SD). Upper case letters indicate significant differencesmain effects between treatments (TukeyHSD, P≤ 0.05). Effects of treatments were considered significant (i.e. different from the controls) when 95% CI did not overlap with zero.

Discussion

Our experimental results suggest that litter type as well as litter amount do influence seedling emergence and establishment.growth, whereas conditioned soil did not when combined with litter treatments. It is interesting to note that although ragwort litter reduced the number of conspecific emerging seeds, seedlings were actually performingperformed better in ragwort litter, i.e. successful ragwort seedlings developed higher showing increased biomass, height and leaf numbers.

Litter type

While both, litter type and litter amount, influenced seedling emergence significantly, litter type accounted for a much higher portion of the variance than litter amount. In contrast, other studies reported that litter amount was more relevant than litter type (43, 44). This highlights the species specific nature of litter effects (13) and is a hint that ragwort litter is indeed autotoxic and negatively influences emergence of conspecific seedlings.

Seedling emergence was highest in grass litter, lowest in ragwort litter and intermediate in the litter mixture. The positive effect of grass litter might be due to physical litter effects like

preserving a relatively constant temperature and soil moisture (45). In our experiment, the same amount of grass litter was covering a larger proportion of the soil surface than ragwort or mixed litter (high amount: grass 90 ± 3% (mean ± SE), mixed 85 ± 4% and ragwort 67 ± 8%, low amount: grass: 85 ± 3%, mixed 70 ± 9%, ragwort 37 ± 5%). In accordance to the study of Eckstein and Donath (45), which revealed a rise in soil moisture when litter cover increased, we assume that seedlings experienced more favourable water availability in the grass litter. Laboratory experiments of van der Meijden et al. (46) demonstrated that seed germination of ragwort increased with higher soil humidity. We also observed that ragwort litter was decaying faster than grass litter (personal observation). This is in line with general findings that litter of forb species as ragwort, tends to decay faster than grass litter (47). Schuster et al. (48) found that the mass of ragwort litter remaining after 30 days was only half of the amount of grass litter. Since the protective properties of a litter cover against desiccation and temperature extremes increases with litter amount present, seedlings emerging from beneath faster decaying ragwort litter might have experienced less protection than those seedlings emerging from beneath grass litter (12, 45). Thus, the negative effect of ragwort litter on ragwort seedlings seems to be a result of fewer physical benefits seedlings experienced from conspecific litter compared to grass litter.

In general, the amelioration of the physical environment by litter, rather than its chemical composition, seems to be the main factor boosting seedling performance (17). However, the size of chemical autotoxic effects and therefore their relative importance in relation to physical and mechanical or other biotic effects are species-specific (49). Several studies show the occurrence of autotoxicity of ragwort (30; 9), which suggests that chemical effects are an additional mechanism for lower seedling emergence from beneath ragwort litter. Inline, van de Voorde (30) reported decreasing germination with increasing concentrations of ragwort extract. As ragwort produces many seeds with high germination potential and low dispersal ability (36,

~~1, 35) autotoxic effects may avoid intraspecific competition in ragwort and thus may lead to lower seedling numbers but increased seedling vitality.~~

Litter type accounted for a much higher portion of the variance than litter amount. In contrast, other studies reported that litter amount was more relevant than litter type (49, 50). This highlights the species specific nature of litter effects (18). One explanation for this pattern could be that ragwort litter is indeed autotoxic and induces self-thinning by negatively influencing the emergence of conspecific seedlings (11).

Seedling emergence was highest in grass litter, lowest in ragwort litter and intermediate in the litter mixture. Germination was highest in medium compared to no or high litter amounts. In contrast, all parameters measuring seedling growth (biomass, height, mean leaf number) were highest in high and/or ragwort litter. The positive effect of grass litter and medium litter amounts on seedling emergence might be due to physical litter effects like preserving a relatively constant temperature and soil moisture (51). In our experiment, the same amount of grass litter covered a larger proportion of the soil surface than ragwort or mixed litter (high amount: grass 90 ± 3%, mixed 85 ± 4% and ragwort 67 ± 8%, low amount: grass: 85 ± 3%, mixed 70 ± 9%, ragwort 37 ± 5%). In accordance with a study by Eckstein and Donath (51), which revealed a rise in soil moisture when litter cover increased, we assume that seedlings experienced more favourable water availability in grass litter and medium litter amounts. In high litter amounts however, negative effects of litter prevailed over positive effects. Laboratory experiments of van der Meijden et al. (16) demonstrated that seed germination of ragwort increased with higher soil humidity. We also observed that ragwort litter decayed faster than grass litter (personal observation). This is in line with general findings that litter of forb species such as ragwort tends to decay faster than grass litter (52). Schuster et al. (53) found that the mass of ragwort litter remaining after 30 days was only half of the amount of grass (*Alopecurus pratensis*) litter. Since the protective properties of a litter cover against desiccation

and temperature extremes increases with litter amount present, seedlings emerging from beneath faster decaying ragwort litter might have experienced less protection than those seedlings emerging beneath grass litter (15, 51). Thus, the negative effect of ragwort litter on ragwort seedlings emergence seems to be a result of fewer physical benefits seedlings experienced from conspecific litter compared to grass litter. In addition, leachate effects may also depend on the specific decomposition rate of different litter types. As leachates released from the litter are declining very rapidly after senescence (54), intensity of seedlings' exposure to leachate depends on the decomposition rate of the litter present. Thus, ragwort seeds and seedlings emerging from ragwort litter, which decays fast (53), were only exposed to the ragwort leachate for a short time. Consequently, we assume that leachate effects on seedling emergence were more pronounced than on seedling growth.

In general, the amelioration of the physical environment by litter, rather than its chemical composition, seems to be the main factor boosting seedling growth in short time scales (21). However, the size of chemical autotoxic effects and therefore their relative importance in relation to physical and mechanical or other biotic effects are species-specific (55). Several studies show the occurrence of autotoxicity of ragwort (36; 11), which suggests that chemical effects are an additional mechanism for lower seedling emergence from beneath ragwort litter. Van de Voorde (36) reported decreasing germination with increasing concentrations of ragwort extract. As ragwort produces many seeds with high germination potential and low dispersal ability (43, 1, 42) autotoxic effects may avoid intraspecific competition in ragwort and thus may lead to lower seedling numbers but increased seedling vitality.

In contrast of Van de Voordes notion of a negative soil-feedback in ragwort (36), we found only weak indications of effects of ragwort conditioned soil on seedling emergence and growth (Table 1). In fact, the only significant effect of conditioned soil occurred on seedling height in controls, i.e. seedlings in conditioned soil grew bigger than in unconditioned soil. This finding

is in contrast to findings of van de Voorde (32), who reported autotoxic effects of ragwort on its seedling development. However, on average the observed difference in plant height between ragwort seedlings growing in conditioned versus those growing in unconditioned soil including control and litter addition treatments was only 2 mm (less than 4 % of the average height of about 54.5 mm). Therefore, we expect that this difference is too small to be of ecological relevance. Mixed litter resulted in fewer emerged seedlings than in grass litter, but more emerged seedlings compared to ragwort litter, which suggests that effects of grass and ragwort litter simply added up to a neutral effect. This is supporting the mass-ratio hypothesis by Grime (18), i.e. mixed litter effects did not deviate positively nor negatively from the expected combined effects. In our study, the effect of mixed litter was not different from the control. Thus, from a management point of view, leaving litter on ragwort infested sites does not reduce or facilitate ragwort establishment.

Opposing to the findings for seedling emergence, seeds in ragwort litter built up more biomass, grew higher and tended to have more leaves than seedlings in grass litter. Biomass is the only variable where we found a significant interaction between litter type and litter amount. This interaction was quantitative and weak compared with the effects of litter type and amount alone (Table 1).

Seedlings emerging from beneath high amounts of ragwort litter built up the highest biomass. This is also diametrical to the findings of van de Voorde et al. (30), who reported lower biomass for seedlings in ragwort influenced environments. These conflicting results could be due to a different methodology. While van de Voorde et al. (30) harvested seedling biomass after 19 days, seedlings in our study were harvested about 50 days later. While van de Voorde's observation is supported by our personal impression, i.e. seedlings looked smaller and thinner early during development, they started to grow bigger after the ragwort litter had decomposed. Therefore, we might have observed a simple effect of density-dependence and intraspecific competition, which is further supported by the fact that lower seedling numbers from beneath

grass litter resulted in seedlings of higher biomass. Already Loydi et al. (43) pointed out, that while litter plays a major role for seedling emergence for instance via phytotoxins, competition is more important for seedling development later during seedling establishment. However, indirect positive effects of reduced seedling emergence in boosting seedling biomass could be especially pronounced in situations where, like in the present experiment, intraspecific competition prevails and may overlain by different environmental constrains under field conditions (11, 50).

Another explanation for superior performance of emerged seedlings in ragwort litter might be an improved availability of nutrients (10), as ragwort litter decomposed quite fast. This is supported by our observation that even though the number of seedlings in the control pot and in the ragwort litter pot with 2 g/dm² was about the same and thus competition seedlings experienced was comparable, biomass of seedlings growing in ragwort litter pots was still higher.

Regarding leaf number, only seedlings in ragwort litter had significantly more leaves than plants in grass or mixed litter. This again might be linked to lower intraspecific competition as there were lower seedling numbers in the ragwort pots or by less physical hampering compared to grass litter (51).

Litter amount

We can affirm that ragwort emergence is influenced by litter amount, albeit less strongly than by litter type. Seedling emergence was lowest from beneath high litter amounts and higher from beneath low litter amounts, although not significantly different from the control. Positive effects could be due to physical effects such as constant moisture and temperature regime discussed above. Still, when litter cover is too high negative effects prevail over positive effects. Accordingly, compared to the control treatment, seedling emergence was lowest from beneath 4 g of litter and not reduced from beneath 2 g of litter cover. In case of ragwort, the reduced

seedling emergence from beneath 4 g of litter is very likely linked to the relatively small seed mass of ragwort (0.22 mg; (52)). The smaller the seeds are, the less resources a seed can spent to successfully penetrate through a litter cover (53). In addition, small seeded species, like ragwort, tend to depend on higher light levels for germination than large seeded species (54, 55). Since photosynthetic active radiation (PAR) decreases with increasing litter cover (45) not only germination will be lower, but seedlings germinated will also lack light resources for successful penetration of the litter cover. This is also in accordance with van der Meijden and van der Waals-Kooi (46), who found that emergence in ragwort significantly decreased when seeds were placed beneath a sand cover of more than 4 mm. Furthermore, Facelli et al. (56) showed that ruderal species, such as ragwort, are potentially negatively impacted by high amounts of litter. Therefore, ragwort seedlings might be reduced by a mulching regime on productive sites were litter amounts are medium to high.

The effects of litter amount on the establishment of seedlings was quite the opposite of the effects on seedling emergence. Thus, seedlings in dense litter got heavier (at least in mixed and ragwort litter), grew higher, and tended to have more leaves than seedlings in lower amounts of litter. This might be again a direct effect of lowered seedling densities in the first place and thus less competition. Another reason for smaller seedlings in lower amounts of litter could be due to the missing investment of seedlings in higher growth when they already reached light (51). Siebenkäs et al. (57) compared seedling growth for seedlings in the same developmental stage as seedlings in our experiment and found that plants tend to grow higher, when there is more shade. Thus, seedlings may grow higher in higher litter amounts to avoid its shade and in order to reach light. Another explanation for heavier and higher seedlings with more leaves could be higher nutrient content related to higher amounts of litter (33).

Conditioned vs. unconditioned soil

We expected potentially most inhibitory effects of ragwort litter on itself on sites that are infested with ragwort for a longer time. However, effects on soil on seedling emergence and establishment were very weak (Table 1). In fact, the only significant effect of conditioned soil occurred on seedling height, i.e. seedlings in unconditioned soil grew bigger than in conditioned soil. In general, this finding is in line with findings of van de Voorde (30), who also reported autotoxic effects of ragwort on its seedling development. However, on average the observed difference in plant height between ragwort seedlings growing in conditioned versus those growing in unconditioned soil was only 2 mm (less than 4% of the average height of about 54.5 mm).In other studies, looking at several seedling cohorts, those cohorts usually differed several centimeters in height (56–58).

Mixed litter resulted in fewer emerged seedlings than in grass litter, but more emerged seedlings compared to ragwort litter, which suggests that the effects of grass and ragwort litter simply added up to a neutral effect. This supports the mass-ratio hypothesis by Grime (22) for the factors seedling number, biomass per plant and mean leaf number, i.e. mixed litter effects did not deviate positively nor negatively from the expected combined effects. However, for biomass per pot and plant height, effects in mixed litter were higher than could be assumed from the simple addition of grass and ragwort. Thus, ragwort growth profited in single aspects from mixed litter layers. Thus, from a management point of view, leaving litter on ragwort infested sites does slightly facilitate ragwort growth when biomass of ragwort and grass is balanced. However, if the site is rather productive and ragwort is dominating the standing biomass, germination of its seedlings might indeed be limited. When litter cover is too high, negative effects prevail over positive effects. Accordingly, compared to the control treatment, seedling emergence was lowest from beneath 400 g of litter and not reduced from beneath 200 g of litter cover. The reduced seedling emergence of ragwort from beneath 400 g of litter is very likely

linked to the relatively small seed mass of ragwort (0.22 mg; (59)). The smaller the seeds are, the less resources a seed can spend to successfully penetrate through a litter cover (60). In addition, small seeded species, like ragwort, tend to depend on higher light levels for germination than large seeded species (61, 62). Since photosynthetic active radiation (PAR) decreases with increasing litter cover (51) not only will germination be lower, but germinated seedlings will also lack light resources for successful penetration of the litter cover. This is also in accordance with van der Meijden and van der Waals-Kooi (16), who found that emergence in ragwort significantly decreased when seeds were placed beneath a sand cover of more than 4 mm. Furthermore, Facelli et al. (63) showed that ruderal species, such as ragwort, tend to be more sensitive and thus more likely to be negatively affected by high amounts of litter. Therefore, ragwort seedlings might be reduced by a mulching regime on productive sites where litter amounts are medium to high.

In contrast to the findings for seedling emergence, seeds in ragwort litter built up more biomass, grew higher and tended to have more leaves than seedlings in grass litter. Biomass and height are the two variables where we found a significant interaction between litter type and litter amount. This could be explained by a reduction in competition for the few seedlings that emerged in ragwort litter. The interaction was quantitative and weak compared with the effects of litter type and amount alone (Table 1). Seedlings emerging from beneath high amounts of litter and/or ragwort litter built up the highest biomass and got tallest. This is also diametrical to the findings of van de Voorde et al. (36), who reported lower biomass for seedlings in ragwort influenced environments. These conflicting results could be due to different methodology. While van de Voorde et al. (36) harvested seedling biomass after 19 days, seedlings in our study were harvested about 50 days later. While van de Voorde's results were supported by our early observations (seedlings looked smaller and thinner early during early development in ragwort litter), seedlings started to grow bigger after ragwort litter had decomposed. Thus, the longer duration of our experiment compared to the experiment by van de Voorde et al. (35) allowed

us to observe a change in the importance of litter effects in relation to intraspecific density dependent competitive effects (49), which is further supported by the fact that lower seedling numbers from beneath grass litter resulted in seedlings of higher biomass and height. Loydi et al. (49) already identified, that while litter plays a major role for seedling emergence, for instance, via phytotoxins, competition is more important for seedling development later during seedling growth. Significantly increased mean leaf numbers in seedlings in high litter amounts and in ragwort litter compared to plants in grass or mixed litter might be linked to lower intraspecific competition as there were fewer seedling numbers in the ragwort pots or by less physical hampering compared to grass litter (60). However, indirect positive effects of reduced seedling emergence in boosting seedling biomass could be especially pronounced in situations where, as in the present experiment, intraspecific competition prevails and may overlay different environmental constrains under field conditions (13, 64).

Another explanation for superior performance of emerged seedlings in high litter amounts and ragwort litter might be an improved availability of nutrients (12), as more biomass contains more nutrients and ragwort litter decomposed quite fast. This is supported by our observation that even though the number of seedlings in the control pot and in the ragwort litter pot with 200 g/m² was about the same and thus competition among seedlings was comparable, biomass of seedlings growing in ragwort litter pots was still higher. However, it is questionable if nutrients from decaying litter are converted to plant available nutrients within 70 days. Effects of different litter amounts on nutrient availability are generally relatively low (49). Another reason for lighter and smaller seedlings in lower amounts of litter could be due to the missing investment of seedlings in higher growth when they have already reached light (65). Siebenkäs et al. (66) compared seedling growth in the same developmental stage as seedlings in our experiment and found that plants tend to grow higher when there is more shade. Thus, seedlings may grow higher in higher litter amounts to avoid its shade and in order to reach light. Renne et al. (2006) found that small differences in height of seedlings early on can produce large

differences in their final biomass (67) indicating that early shading can translate in higher heights, even if shade is gone.

Therefore, we expect that this difference is too small to be of ecological relevance. In other studies, looking at several seedling cohorts, those cohorts usually differed several centimeters in height (58–60).

Conclusion

We found both negative and positive effects of ragwort litter on conspecific seedling emergence and establishment.growth. While negative effects can be most likely linked to autotoxic chemical and physical ragwort litter effects in the stage of seedling emergence, those seedlings that emerged successfully may have profited from a lower intraspecific competition and may had more resources to invest than seedlings that emerged from beneath grass or mixed litter or they profited from higher nutrient amounts in ragwort litter. Due to the ambivalent nature of our results, these findings cannot easily be translated into management recommendations for ragwort-infested sites. However, the effects of mixed litter on ragwort recruitment did not differ from the control treatment.were either neutral or even promoted ragwort growth. Therefore, when we assume that biomass on mowed ragwort pastures consists roughly of similar amounts of grass and ragwort, we expect that leaving litter containing ragwort biomass on the grassland will have no regulating influence on recruitment of ragwort seedlings. negative effect on ragwort population density or growth. As already our results for a simple controlled experiment do not lead to a clear hint of the effectiveness of ragwort litter in reducing ragwort population density, we do not expect pronounced effects in an experiment under more natural conditions.

References

1. Wardle DA. The ecology of ragwort (Senecio jacobaea L.): A Review. New Zealand Journal of Ecology 1987:67–76.

2. Leiss KA. Management practices for control of ragwort species. Phytochem Rev 2011; 10(1):153–63.

3. McEvoy PB, Cox C, Coombs E. Successful biological control of ragwort, Senecio jacobaea, by introduced insects in Oregon. Ecological Applications 1991; 1(4):430–42.

4. Suter M, Lüscher A. Measures for the control of Senecio aquaticus in managed grassland. Weed Research 2011; 51(6):601–11.

5. Jacobs J, Sing S. Ecology and Management of Tansy Ragwort (Senecio jacobaea L.). NRCS Montana Technical Notes-Invasive Species-MT-24 2009.

6. Siegrist-Maag S, Lüscher A, Suter M. Reaktion von Jakobs-Kreuzkraut auf Schnitt. Agrarforschung (Switzerland) 2008; 15(7):338–43.

7. Huckauf A, Rabe I, Aboling S, Böhling J, Böttner E, Ehlers B et al. Umgang mit dem Jakobs-Kreuzkraut: Meiden - dulden - bekämpfen. 4. überarbeitete Auflage. Flintbek: Landesamt für Landwirtschaft, Umwelt und ländliche Räume des Landes Schleswig-Holstein (LLUR); 2017. (Schriftenreihe LLUR SH. Natur; vol 25). Available from: URL: https://www.stiftungsland.de/fileadmin/pdf/JKK/senecio.pdf.

8. Wang R, Yang X, Song Y, Zhang M, Linhu, Su Y et al. Allelopathic potential of Tephrosia vogelii Hook. f.: Laboratory and field evaluation. Allelopathy Journal 2011; 28(1):53–62. Available from: URL: https://www.researchgate.net/profile/Ruilong_Wang/publication/287677172_Allelopathic_potential_of_Tephrosia_vogelii_Hook_f_Laboratory_and_field_evaluation/links/58735d0308ae8fce492390aa/Allelopathic-potential-of-Tephrosia-vogelii-Hook-f-Laboratory-and-field-evaluation.pdf.

9. Möhler H, Diekötter T, Herrmann JD, Donath TW. Allelopathic vs. autotoxic potential of a grassland weed—evidence from a seed germination experiment. Plant Ecology & Diversity 2018; 11(4):539–49.

10. Facelli JM, Pickett STA. Plant litter: Its dynamics and effects on plant community structure. The Botanical Review 1991; 57(1):1–32. Available from: URL: https://link.springer.com/content/pdf/10.1007%2FBF02858763.pdf.

11. Loydi A, Eckstein RL, Otte A, Donath TW, Gómez-Aparicio L. Effects of litter on seedling establishment in natural and semi-natural grasslands: A meta-analysis. J Ecol 2013; 101(2):454–64.

12. Loydi A, Lohse K, Otte A, Donath TW, Eckstein RL. Distribution and effects of tree leaf litter on vegetation composition and biomass in a forest–grassland ecotone. Journal of Plant Ecology 2014; 7(3):264–75.

13. Bonanomi G, Sicurezza MG, Caporaso S, Esposito A, Mazzoleni S. Phytotoxicity dynamics of decaying plant materials. New Phytol 2006; 169(3):571–8.

14. van der Putten, Wim H., Bardgett RD, Bever JD, Bezemer TM, Casper BB, Fukami T et al. Plant-soil feedbacks: The past, the present and future challenges. J Ecol 2013; 101(2):265–76.

15. Xiong S, Nilsson C. The effects of plant litter on vegetation: A meta-analysis. Journal of Ecology 1999; (87):984–94.

16. Donath TW, Eckstein RL. Grass and oak litter exert different effects on seedling emergence of herbaceous perennials from grasslands and woodlands. J Ecology 2008; 96(2):272–80.

17. Quested H, Eriksson O. Litter species composition influences the performance of seedlings of grassland herbs. Functional Ecology 2006; (20):522–32.

18. Grime JP. Benefits of plant diversity to ecosystems: immediate, filter and founder effects. J Ecol 1998; (86):902–10.

19. Gartner TB, Cardon ZG. Decomposition dynamics in mixed-species leaf litter. Oikos 2004; (104):230–46.

20. Antonovics J, Levin DA. The Ecological and Genetic Consequences of Density-Dependent Regulation in Plants. Ann. Rev. Ecol. Syst. 1980; (11):411–52.

21. Pleasants J M, Jurik T W. Dispersion of seedlings of the prairie compass plant, Silphium laciniatum (Asteraceae). American Journal of Botany 1992; (79):133–7.

22. Roll J, Mitchell RJ, Cabin RJ, Marshall DL. Reproductive Success Increases with Local Density of Conspecifics in a Desert Mustard (Lesquerella fendleri). Conservation Biology 1997; (11):738–46.

23. Montesinos D, García-Fayos P, Mateu I. Conflicting selective forces underlying seed dispersal in the endangered plant Silene diclinis. Int. J. Plant Sci. 2006; 167(1):103–10.

24. McEvoy PB. Seedling dispersion and the persistence of ragwort Senecio jacobaea (Compositae) in a grassland dominated by perennial species. Oikos 1984; Vol. 42(No. 2):138–43.

25. Wardle DA, Ahmed M, Nicholson KS. Allelopathic influence of nodding thistle (Carduus nutans L.) seeds on germination and radicle growth of pasture plants. New Zealand Journal of Agricultural Research 1991; 34(2):185–91.

26. Reinhart KO, Maestre FT, Callaway RM. Facilitation and inhibition of seedlings of an invasive tree (Acer platanoides) by different tree species in a mountain ecosystem. Biol Invasions 2006; (8):231–40.

27. Singh HP, Batish DR, Kohli RK. Autotoxicity: Concept, organisms, and ecological significance. Crit Rev Plant Sci 1999; (18):757–72.

28. Suzuki RO, Kudoh H, Kachi N. Spatial and temporal variations in mortality of the biennial plant, Lysimachia rubida: effects of intraspecific competition and environmental heterogeneity. J Ecology 2003; (91):114–25.

29. Hovstad KA, Ohlson M. Conspecific versus heterospecific litter effects on seedling establishment. Plant Ecol 2009; 204(204):33–42. Available from: URL: https://link.springer.com/content/pdf/10.1007%2Fs11258-008-9563-2.pdf.

30. van de Voorde, Tess F.J., Ruijten M, van der Putten, Wim H., Bezemer TM. Can the negative plant–soil feedback of Jacobaea vulgaris be explained by autotoxicity? Basic and Applied Ecology 2012; 13(6):533–41.

31. Bezemer TM, Harvey JA, Kowalchuk GA, Kopershoek H, van der Putten, Wim H. Interplay between Senecio jacobaea and Plant, Soil, and Aboveground Insect Community Composition. Ecology 2006:2002–13.

32. van de Voorde, Tess F. J., van der Putten, Wim H., Martijn Bezemer T. Intra- and interspecific plant-soil interactions, soil legacies and priority effects during old-field succession. Journal of Ecology 2011; 99(4):945–53.

33. Loydi, A. Donath, T. W. Eckstein, R. L. Otte, A. Non-native species litter reduces germination and growth of resident forbs and grasses: allelopathic, osmotic or mechanical effects? 2014 .

34. Harper JL, Wood WA. Biological Flora of the British Isles: Senecio jacobaea. Journal of Ecology 1957; 45(2):617–37.

35. McEvoy PB, Cox CS. Wind Dispersal Distances in Dimorphic Achenes of Ragwort, Senecio Jacobaea. Ecology 1987; 68(6):2006–15.

36. Crawley N. The Establishment of Seedlings from Primary and Regrowth Seeds of Ragwort (Senecio Jacobaea). Journal of Ecology 1985; 73(1):255–61.

37. Smit HJ, Metzger MJ, Ewert F. Spatial distribution of grassland productivity and land use in Europe. Agricultural Systems 2008; (98):208–19.

38. ISTA. Chapter 6: Biochemical test for viability: the topographical tetrazolium test. Int. rules seed test. 2015; 2015(1):6.

39. van de Voorde, Tess F. J., van der Putten, Wim H., Bezemer TM. The importance of plant-soil interactions, soil nutrients, and plant life history traits for the temporal dynamics of Jacobaea vulgaris in a chronosequence of old-fields. Oikos 2012; 121(8):1251–62.

40. Schindelin J, Rueden C T, Hiner M C, Eliceiri KW. The ImageJ ecosystem: An open platform for biomedical image analysis. Molecular Reproduction and Development 2015; (82):518–29.

41. Koorem K, Price JN, Moora M. Species-specific effects of woody litter on seedling emergence and growth of herbaceous plants. PLoS One 2011; 6(10):e26505. Available from: URL: http://journals.plos.org/plosone/article/file?id=10.1371/journal.pone.0026505&type=printable.

42. R: A language and environment for statistical computing. Vienna, Austria: R Foundation for; 2019. Available from: URL: https://www.R-project.org/.

43. Loydi A, Donath TW, Otte A, Eckstein RL. Negative and positive interactions among plants: Effects of competitors and litter on seedling emergence and growth of forest and grassland species. Plant Biol J 2015; 17(3):667–75.

44. Myster RW. Species-specific effects of grass litter mass and type on emergence of three tall grass prairie species. Ecoscience 2006; (13):95–9.

45. Eckstein RL, Donath TW. Interactions between litter and water availability affect seedling emergence in four familial pairs of floodplain species. Journal of Ecology 2005; 93(4):807–16.

46. van der Meijden E, van der Waals-Kooi, R. E. The Population Ecology of Senecio Jacobaea in a Sand Dune System: I. Reproductive Strategy and the Biennial Habit. Journal of Ecology 1979; 67(1):131–53.

47. Shaw RM, Harte J. Control of litter decomposition in a subalpine meadow–sagebrush steppe ecotone under climate change. Ecological Applications 2001; 11(4):1206–23.

48. Schuster MJ, Kreyling J, Berwaers S, Walter J, Boeck HJ de, Dengler J et al. Drought inhibits synergistic interactions of native and exotic litter mixtures during decomposition in temperate grasslands. Plant Soil 2017; (415):257–68.

49. Smith-Ramesh LM, Reynolds HL. The next frontier of plant–soil feedback research: unraveling context dependence across biotic and abiotic gradients. Journal of Vegetation Science 2017; (28):484–94.

50. Cavieres LA, Peñaloza A. Facilitation and interference at the intraspecific level: Recruitment of Kageneckia angustifolia D. Don (Rosaceae) in the montane sclerophyllous woodland of central Chile. Perspectives in Plant Ecology, Evolution and Systematics 2012; 14(1):13–9. Available from: URL:

https://ac.els-cdn.com/S1433831911000540/1-s2.0-S1433831911000540-main.pdf?_tid=504eb97b-fcf2-4e74-a32d-3bd51b9a3455&acdnat=1524728836_3de92c92b53c85c032201f4918134413.

51. Olson BE, Wallander RT. Effects of invasive forb litter on seed germination, seedling growth and survival. Basic and Applied Ecology 2002; 3(4):309–17. Available from: URL: https://ac.els-cdn.com/S1439179104700882/1-s2.0-S1439179104700882-main.pdf?_tid=1fcfe502-3db1-4ea1-8218-75099da6c252&acdnat=1524583667_44dede2fb9ae6a6adab43a7e509ac07d.

52. Fenner M. Relationships between seed weight, ash content and seedling growth in twenty-four species of Compositae. New Phytol 1983; (95):697–706.

53. Donath TW, Eckstein RL. Effects of bryophytes and grass litter on seedling emergence vary by vertical seed position and seed size. Plant Ecol 2010; (207):257–68.

54. Milberg P, Andersson L, Thompson K. Large-seeded spices are less dependent on light for germination than small-seeded ones. SSR 2000; 10(01):99. Available from: URL: https://www.cambridge.org/core/services/aop-cambridge-core/content/view/D6EC82DEDAAFE10F20C1D3A9A2328879/S0960258500000118a.pdf/largeseeded_spices_are_less_dependent_on_light_for_germination_than_smallseeded_ones.pdf.

55. Baskin CC, Baskin JM. Seeds: Ecology, Biogeography, and Evolution of Dormacy and Germination. Lexington: Academic Press; 2014.

56. Facelli JM, Montero CM, Leon R. Effect of different disturbance regimen on seminatural grasslands from the subhumid pampa. Flora - Morphology, Distribution, Functional Ecology of Plants 1988; (180):241–9.

57. Siebenkäs A, Schumacher J, Roscher C. Phenotypic plasticity to light and nutrient availability alters functional trait ranking across eight perennial grassland species. AoB Plants 2015; 7. Available from: URL: https://watermark.silverchair.com/plv029.pdf?token=AQECAHi208BE49Ooan9kkhW_Ercy7Dm3ZL_9Cf3qfKAc485ysgAAAbswggG3BgkqhkiG9w0BBwagggGoMIIBpAIBADCCAZ0GCSqGSIb3DQEHATAeBglghkgBZQMEAS4wEQQM2jFuXu_BEIq63rbbAgEQgIIBblqXOQs0WLQr398Ioj6Lq5NBvIL6i6Ov2dHtQb8B1no6uKgq0lmSDgTGVLdI-AbNlb8D2pjghvdZ_4wEcOGMPWB3xDsWcXnI2zlKInkXUvpipk7ODNpsUX_VY-4IJ5ln87uH2NPi53vrRCcPBxH70FmTAk8l-q0qrdE_5F9jHTgSdnjMjSikXysP1wvigdxiUm0QMjXUnueHzXeIE3GNyIjygSStnxLrn43gbYuyEdbQ1O65PrawU5L7-IfUgpu1SGXyfTGzW66A-ACjo2Uvg2GR0FmGvJ0ovOTjnTa7NymiQuF1QAlJ9YStjNdgCFklZbvpY9XK9wgXmrOwR2RI9qbqIHS_g8ZHLIKJ2TNggHoSonNyoJZLdYadJrUy7OeRNTHAwHUImj4dIiQ3rBmER-Wz8qCABafRrSbGO5mFJI9sf93cNIThTy7lA5AMU4bmKMIEKl4vEa7EY0mopASvev4VQSg-1yt0uOyhjLP1Lg.

58. Seiwa K. Effects of seed size and emergence time on tree seedling establishment: importance of developmental constraints. Oecologia 2000; (123):208–15.

59. Pausas JG, Ouadah N, Ferran A, Gimeno T, Vallejo R. Fire severity and seedling establishment in Pinus halepensis woodlands, eastern Iberian Peninsula. Plant Ecol 2003; (169):205–13.

60. Quintana J, Cruz A, Fernández-González F, Moreno JM. Time of germination and establishment success after fire of three obligate seeders in a Mediterranean shrubland of central Spain. Journal of Biogeography 2004; (31):241–9.

Acknowledgements

We thank Astrid Dempfle for her support in statistical analyses. Furthermore, we thank Hollyn Hartlep for her valuable help in proofreading. The research in this article was funded by the Stiftung-Naturschutz Schleswig-Holstein.

Literature Cited

1. Wardle DA. The ecology of ragwort (*Senecio jacobaea* L.): A review. New Zealand Journal of Ecology 1987 [cited 2016 Feb 25]; 10:67–76.

2. Leiss KA. Management practices for control of ragwort species. Phytochemistry Reviews 2011; 10(1):153–63.

3. McEvoy PB, Cox C, Coombs E. Successful biological control of ragwort, *Senecio jacobaea*, by introduced insects in Oregon. Ecological Applications 1991; 1(4):430–42.

4. Suter M, Lüscher A. Measures for the control of *Senecio aquaticus* in managed grassland. Weed Research 2011 [cited 2016 Apr 27]; 51(6):601–11.

5. Jacobs J, Sing S. Ecology and management of tansy ragwort (*Senecio jacobaea* L.). Montana: Natural Resources Conservation Service; 2009.

6. Siegrist-Maag S, Lüscher A, Suter M. Reaktion von Jakobs-Kreuzkraut auf Schnitt. Agrarforschung (Switzerland) 2008; 15(7):338–43.

7. Kaltner F, Rychlik M, Gareis M, Gottschalk C. Influence of storage on the stability of toxic pyrrolizidine alkaloids and their N-Oxides in peppermint tea, hay, and honey. J Agric Food Chem 2018; 66(20):5221–8. Available from: URL: https://doi.org/10.1021/acs.jafc.7b06036.

8. Neumann H, Lütt S, Schleich-Saidfar C, Rabe I, Walter A, Böhling J et al. Umgang mit dem Jakobs-Kreuzkraut: Meiden - Dulden - Bekämpfen. Flintbek: LLUR; 2009. (LLUR SH / Natur; vol 14) [cited 2016 Dec 12].

9. Huckauf A, Rabe I, Aboling S, Böhling J, Böttner E, Ehlers B et al. Umgang mit dem Jakobs-Kreuzkraut: Meiden - dulden - bekämpfen. 4. überarbeitete Auflage. Flintbek: Landesamt für Landwirtschaft, Umwelt und ländliche Räume des Landes Schleswig-Holstein (LLUR); 2017. (Schriftenreihe LLUR SH. Natur; vol 25) [cited 2018 Mar 19]. Available from: URL: https://www.stiftungsland.de/fileadmin/pdf/JKK/senecio.pdf.

10. Wang R, Yang X, Song Y, Zhang M, Linhu, Su Y et al. Allelopathic potential of Tephrosia vogelii Hook. f.: Laboratory and field evaluation. Allelopathy Journal 2011 [cited 2018 Apr 23]; 28(1):53–62. Available from: URL: https://www.researchgate.net/profile/Ruilong_Wang/publication/287677172_Allelopathic_potential_of_Tephrosia_vogelii_Hook_f_Laboratory_and_field_evaluation/links/58735d0308ae8fce492390aa/Allelopathic-potential-of-Tephrosia-vogelii-Hook-f-Laboratory-and-field-evaluation.pdf.

11. Möhler H, Diekötter T, Herrmann JD, Donath TW. Allelopathic vs. autotoxic potential of a grassland weed—evidence from a seed germination experiment. Plant Ecology & Diversity 2018 [cited 2020 Jul 15]; 11(4):539–49.

Formatted: German (Germany)

Formatted: German (Germany)

12. Facelli JM, Pickett STA. Plant litter: Its dynamics and effects on plant community structure. The Botanical Review 1991 [cited 2018 Apr 23]; 57(1):1–32. Available from: URL: https://link.springer.com/content/pdf/10.1007%2FBF02858763.pdf.

13. Loydi A, Eckstein RL, Otte A, Donath TW, Gómez-Aparicio L. Effects of litter on seedling establishment in natural and semi-natural grasslands: A meta-analysis. J Ecol 2013 [cited 2016 Apr 18]; 101(2):454–64.

14. Xiong S, Nilsson C. The effects of plant litter on vegetation: A meta-analysis. Journal of Ecology 1999 [cited 2018 Apr 23]; (87):984–94.

15. Loydi A, Lohse K, Otte A, Donath TW, Eckstein RL. Distribution and effects of tree leaf litter on vegetation composition and biomass in a forest–grassland ecotone. Journal of Plant Ecology 2014; 7(3):264–75.

16. van der Meijden E, van der Waals-Kooi RE. The population ecology of *Senecio jacobaea* in a sand dune system: I. Reproductive strategy and the biennial habit. Journal of Ecology 1979; 67(1):131–53.

17. Baskin CC, Baskin JM. Seeds: ecology, biogeography, and evolution of dormancy and germination. 2nd ed. Amsterdam: Elsevier; 2014.

18. Bonanomi G, Sicurezza MG, Caporaso S, Esposito A, Mazzoleni S. Phytotoxicity dynamics of decaying plant materials. New Phytol 2006 [cited 2016 Apr 11]; 169(3):571–8.

19. van der Putten WH, Bardgett RD, Bever JD, Bezemer TM, Casper BB, Fukami T et al. Plant-soil feedbacks: The past, the present and future challenges. J Ecol 2013 [cited 2016 Apr 6]; 101(2):265–76.

20. Donath TW, Eckstein RL. Grass and oak litter exert different effects on seedling emergence of herbaceous perennials from grasslands and woodlands. Journal of Ecology 2008; 96(2):272–80. Available from: URL: https://besjournals.onlinelibrary.wiley.com/doi/pdf/10.1111/j.1365-2745.2007.01338.x.

21. Quested H, Eriksson O. Litter species composition influences the performance of seedlings of grassland herbs. Functional Ecology 2006 [cited 2020 Apr 27]; 20:522–32.

22. Grime JP. Benefits of plant diversity to ecosystems: immediate, filter and founder effects. J Ecol 1998 [cited 2020 Apr 27]; 86:902–10.

23. Gartner TB, Cardon ZG. Decomposition dynamics in mixed-species leaf litter. Oikos 2004 [cited 2020 Apr 27]; 104:230–46.

24. Antonovics J, Levin DA. The ecological and genetic consequences of density-dependent regulation in plants. Ann. Rev. Ecol. Syst. 1980 [cited 2020 Apr 27]; 11(11):411–52.

25. Adler PB, Smull D, Beard KH, Choi RT, Furniss T, Kulmatiski A et al. Competition and coexistence in plant communities: Intraspecific competition is stronger than interspecific competition. Ecology Letters 2018; 21(9):1319–29. Available from: URL: https://onlinelibrary.wiley.com/doi/pdf/10.1111/ele.13098.

26. Pleasants JM, Jurik TW. Dispersion of seedlings of the prairie compass plant, *Silphium laciniatum* (Asteraceae). American Journal of Botany 1992; 79:133–7.

27. Roll J, Mitchell RJ, Cabin RJ, Marshall DL. Reproductive success increases with local density of conspecifics in a desert mustard (*Lesquerella fendleri*). Conservation Biology 1997 [cited 2020 Apr 27]; 11(11):738–46.

28. Montesinos D, García-Fayos P, Mateu I. Conflicting selective forces underlying seed dispersal in the endangered plant *Silene diclinis*. Int. J. Plant Sci. 2006 [cited 2020 Apr 27]; 167(1):103–10.

29. McEvoy PB. Seedling dispersion and the persistence of ragwort Senecio jacobaea (Compositae) in a grassland dominated by perennial species. Oikos 1984; Vol. 42(No. 2):138–43.

30. Wardle DA, Ahmed M, Nicholson KS. Allelopathic influence of nodding thistle (*Carduus nutans* L.) seeds on germination and radicle growth of pasture plants. New Zealand Journal of Agricultural Research 1991 [cited 2020 Apr 27]; 34(2):185–91.

31. Reinhart KO, Maestre FT, Callaway RM. Facilitation and inhibition of seedlings of an invasive tree (*Acer platanoides*) by different tree species in a mountain ecosystem. Biol Invasions 2006 [cited 2020 Apr 27]; 8(8):231–40.

32. Singh HP, Batish DR, Kohli RK. Autotoxicity: Concept, organisms, and ecological significance. Crit Rev Plant Sci 1999; 18:757–72.

33. Suzuki RO, Kudoh H, Kachi N. Spatial and temporal variations in mortality of the biennial plant, *Lysimachia rubida*: Effects of intraspecific competition and environmental heterogeneity. J Ecology 2003 [cited 2020 Apr 27]; 91:114–25.

34. Hovstad KA, Ohlson M. Conspecific versus heterospecific litter effects on seedling establishment. Plant Ecol 2009 [cited 2018 Apr 19]; 204(204):33–42. Available from: URL: https://link.springer.com/content/pdf/10.1007%2Fs11258-008-9563-2.pdf.

35. Campanella MV, Bisigato AJ. Conspecific leaf litter and root competition inhibits shrub emergence in the Patagonian steppe. Plant Ecol 2019; 220(10):985–93.

36. van de Voorde TFJ, Ruijten M, van der Putten WH, Bezemer TM. Can the negative plant–soil feedback of *Jacobaea vulgaris* be explained by autotoxicity? Basic and Applied Ecology 2012 [cited 2020 Apr 28]; 13(6):533–41.

37. Bezemer TM, Harvey JA, Kowalchuk GA, Kopershoek H, van der Putten WH. Interplay between *Senecio jacobaea* and plant, soil, and aboveground insect community composition. Ecology 2006; 87(8):2002–13.

38. van de Voorde TFJ, van der Putten WH, Martijn Bezemer T. Intra- and interspecific plant-soil interactions, soil legacies and priority effects during old-field succession. Journal of Ecology 2011; 99(4):945–53.

39. Loydi A, Donath TW, Eckstein RL, Otte A. Non-native species litter reduces germination and growth of resident forbs and grasses: allelopathic, osmotic or mechanical effects? Biol Invasions 2015 [cited 2016 Mar 31]; 17:581–95.

40. Begon M, Townsend CR, Harper JL. Ecology: From Individuals to Ecosystems. 4th ed.: Blackwell Publishing; 2011.

41. Harper JL, Wood WA. Biological Flora of the British Isles: *Senecio jacobaea*. Journal of Ecology 1957; 45(2):617–37.

42. McEvoy PB, Cox CS. Wind dispersal distances in dimorphic achenes of ragwort, *Senecio jacobaea*. Ecology 1987; 68(6):2006–15.

43. Crawley M, Nachapong M. The establishment of seedlings from primary and regrowth seeds of ragwort (*Senecio jacobaea*). Journal of Ecology 1985; 73(1):255–61.

44. Smit HJ, Metzger MJ, Ewert F. Spatial distribution of grassland productivity and land use in Europe. Agricultural Systems 2008 [cited 2020 Apr 27]; 98(98):208–19.

45. ISTA. Chapter 6: Biochemical test for viability: the topographical tetrazolium test. Int. rules seed test. 2015 [cited 2017 Oct 20]; 2015(1):6.

46. van de Voorde TFJ, van der Putten WH, Bezemer TM. The importance of plant-soil interactions, soil nutrients, and plant life history traits for the temporal dynamics of *Jacobaea vulgaris* in a chronosequence of old-fields. Oikos 2012 [cited 2016 Feb 9]; 121(8):1251–62.

47. Schindelin J, Rueden CT, Hiner MC, Eliceiri KW. The ImageJ ecosystem: An open platform for biomedical image analysis. Molecular Reproduction and Development 2015; 82(82):518–29.

48. R: A language and environment for statistical computing. Vienna, Austria: R Foundation for Statistical Computing; 2019. Available from: URL: https://www.R-project.org/.

49. Loydi A, Donath TW, Otte A, Eckstein RL. Negative and positive interactions among plants: Effects of competitors and litter on seedling emergence and growth of forest and grassland species. Plant Biol J 2015 [cited 2016 Apr 25]; 17(3):667–75.

50. Myster RW. Species-specific effects of grass litter mass and type on emergence of three tall grass prairie species. Ecoscience 2006; 13(13):95–9.

51. Eckstein RL, Donath TW. Interactions between litter and water availability affect seedling emergence in four familial pairs of floodplain species. Journal of Ecology 2005 [cited 2016 Apr 21]; 93(4):807–16.

52. Shaw RM, Harte J. Control of litter decomposition in a subalpine meadow–sagebrush steppe ecotone under climate change. Ecological Applications 2001 [cited 2020 Apr 27]; 11(4):1206–23.

53. Schuster MJ, Kreyling J, Berwaers S, Walter J, Boeck HJ de, Dengler J et al. Drought inhibits synergistic interactions of native and exotic litter mixtures during decomposition in temperate grasslands. Plant Soil 2017 [cited 2020 Apr 27]; 415(415):257–68.

54. Sydes C, Grime JP. Effects of tree leaf litter on herbaceous vegetation in deciduous woodland: II. An experimental investigation. The Journal of Ecology 1981; 69(1):249–62. Available from: URL: http://dx.doi.org/10.2307/2259829.

55. Smith-Ramesh LM, Reynolds HL. The next frontier of plant–soil feedback research: unraveling context dependence across biotic and abiotic gradients. Journal of Vegetation Science 2017 [cited 2020 Apr 27]; 28(28):484–94.

56. Seiwa K. Effects of seed size and emergence time on tree seedling establishment: importance of developmental constraints. Oecologia 2000 [cited 2020 Apr 27]; 123(123):208–15.

57. Pausas JG, Ouadah N, Ferran A, Gimeno T, Vallejo R. Fire severity and seedling establishment in Pinus halepensis woodlands, eastern Iberian Peninsula. Plant Ecol 2003 [cited 2020 Apr 27]; 169(169):205–13.

58. Quintana J, Cruz A, Fernández-González F, Moreno JM. Time of germination and establishment success after fire of three obligate seeders in a Mediterranean shrubland of central Spain. Journal of Biogeography 2004 [cited 2020 Apr 27]; 31(31):241–9.

59. Fenner M. Relationships between seed weight, ash content and seedling growth in twenty-four species of Compositae. New Phytol 1983 [cited 2020 Apr 27]; 95(95):697–706.

60. Donath TW, Eckstein RL. Effects of bryophytes and grass litter on seedling emergence vary by vertical seed position and seed size. Plant Ecol 2010 [cited 2020 Apr 27]; 207:257–68.

61. Milberg P, Andersson L, Thompson K. Large-seeded spices are less dependent on light for germination than small-seeded ones. SSR 2000 [cited 2018 Apr 19]; 10(01):99. Available from: URL:

https://www.cambridge.org/core/services/aop-cambridge-core/content/view/D6EC82DEDAAFE10F20C1D3A9A2328879/S0960258500000118a.pdf/largeseeded_spices_are_less_dependent_on_light_for_germination_than_smallseeded_ones.pdf.

62. Baskin CC, Baskin JM. Seeds: Ecology, Biogeography, and Evolution of Dormacy and Germination. Lexington: Academic Press; 2014.

63. Facelli JM, Montero CM, Leon RJC. Effect of different disturbance regimen on seminatural grasslands from the subhumid pampa. Flora - Morphology, Distribution, Functional Ecology of Plants 1988; 180:241–9.

64. Cavieres LA, Peñaloza A. Facilitation and interference at the intraspecific level: Recruitment of Kageneckia angustifolia D. Don (Rosaceae) in the montane sclerophyllous woodland of central Chile. Perspectives in Plant Ecology, Evolution and Systematics 2012 [cited 2018 Apr 26]; 14(1):13–9. Available from: URL: https://ac.els-cdn.com/S1433831911000540/1-s2.0-S1433831911000540-main.pdf?_tid=504eb97b-fcf2-4e74-a32d-3bd51b9a3455&acdnat=1524728836_3de92c92b53c85c032201f4918134413.

65. Olson BE, Wallander RT. Effects of invasive forb litter on seed germination, seedling growth and survival. Basic and Applied Ecology 2002; 3(4):309–17. Available from: URL: http://dx.doi.org/10.1078/1439-1791-00127.

66. Siebenkäs A, Schumacher J, Roscher C. Phenotypic plasticity to light and nutrient availability alters functional trait ranking across eight perennial grassland species. AoB PLANTS 2015; 7.

67. Renne IJ, Tracy BF, Colonna IA. Shifts in grassland invasibility: Effects of soil resources, disturbance, composition, and invader size. Ecology 2006; 87(9):2264–77.

Supporting Information

S1 Table. Raw data for seedling emergence and seedling ~~establishment~~growth according to different treatments.

S2 Table. Results of a three-way ANOVA on the effects of litter type, litter amount and soil on ln biomass per plant.

Fig S1. Biomass per plant - effects of the two-way interaction of litter type and litter amount. In the box plots middle lines represent median, boxes represent the first and third quartiles, lower and upper bars represent the minimum and the maximum and points represent

outliers (i.e. points above 1.5 SD). Grey: 200 g/m^2 litter, black: 400 g/m^2 litter. Lower case letters indicate significant interactions between treatments (TukeyHSD, $P \leq 0.05$).
